# Immune and stromal scoring system associated with tumor microenvironment and prognosis: a gene-based multi-cancer analysis

**DOI:** 10.1186/s12967-021-03002-1

**Published:** 2021-08-03

**Authors:** Zihang Zeng, Jiali Li, Jianguo Zhang, Yangyi Li, Xingyu Liu, Jiarui Chen, Zhengrong Huang, Qiuji Wu, Yan Gong, Conghua Xie

**Affiliations:** 1 Department of Radiation and Medical Oncology, Zhongnan Hospital of Wuhan University, Wuhan, China; 2 Department of Biological Repositories, Zhongnan Hospital of Wuhan University, Wuhan, China; 3 Tumor Precision Diagnosis and Treatment Technology and Translational Medicine, Hubei Engineering Research Center, Zhongnan Hospital of Wuhan University, Wuhan, China; 4 Hubei Key Laboratory of Tumor Biological Behaviors, Zhongnan Hospital of Wuhan University, Wuhan, China; 5 Hubei Cancer Clinical Study Center, Zhongnan Hospital of Wuhan University, Wuhan, China

**Keywords:** Tumor microenvironment, Multi-task learning, Scoring system, Prognosis, Immunotherapy

## Abstract

**Background:**

Tumor microenvironment (TME) is associated with tumor progression and prognosis. Previous studies provided tools to estimate immune and stromal cell infiltration in TME. However, there is still a lack of single index to reflect both immune and stromal status associated with prognosis and immunotherapy responses.

**Methods:**

A novel immune and stromal scoring system named ISTMEscore was developed. A total of 15 datasets were used to train and validate this system, containing 2965 samples from lung adenocarcinoma, skin cutaneous melanoma and head and neck squamous cell carcinoma.

**Results:**

The patients with high immune and low stromal scores (HL) were associated with low ratio of T cell co-inhibitory/stimulatory molecules and low levels of angiogenesis markers, while the patients with low immune and high stromal scores (LH) had the opposite characteristics. The HL patients had immune-centered networks, while the patients with low immune and low stromal scores (LL) had desert-like networks. Moreover, copy number alteration burden was decreased in the HL patients. For the clinical characteristics, our TME classification was an independent prognostic factor. In the 5 cohorts with immunotherapy, the LH patients were linked to the lowest response rate.

**Conclusions:**

ISTMEscore system could reflect the TME status and predict the prognosis. Compared to previous TME scores, our ISTMEscore was superior in the prediction of prognosis and immunotherapy response.

**Supplementary Information:**

The online version contains supplementary material available at 10.1186/s12967-021-03002-1.

## Background

With the discovery of immune checkpoint molecules, immunotherapy becomes more promising strategy for cancer patients to elicit clinical responses durably [1]. It has been approved for the treatment of lung adenocarcinoma (LUAD), skin cutaneous melanoma (SKCM), and head and neck squamous cell carcinoma (HNSC). These tumors have high PD-L1 and CD8A expression levels [2]. Nevertheless, immunotherapy only benefits a minor subset of patients for long-term survival [3]. Identification of potentially therapeutic indexes linked to tumor prognosis and immunotherapy responses will remarkedly contributed to precision medicine.

Tumor microenvironment (TME) consists of immune and non-immune stromal components, both of which were reported to be closely associated with oncogenesis and malignant behaviors of tumors [4]. The existing evidences demonstrate that abundant immune components in TME are positively associated with immunotherapy responses [5]. To date, a range of algorithms have been developed to estimate the immune and stromal cell infiltration including CIBERSORT, TIMER, ESTIMATE and MCPcounter [6–9]. These tools perform well in the estimation of TME cells, but not in the prediction of tumor prognosis and immunotherapy responses. Although ESTIMATE provides the immune and stromal infiltration scores [8], LUAD, SKCM and HNSC were not included in the training datasets. There is still a lack of single index to reflect both immune and stromal activation signals associated with prognosis and immunotherapy responses.

Here we developed a novel immune and stromal scoring system named ISTMEscore, which followed a unique design: (1) Isolate TME signals associated with prognosis from bulk gene expression data; (2) Extract specific gene signatures from the above TME signals; (3) Calculate ISTME scores with single-sample gene set enrichment analysis (ssGSEA) algorithm [10]. In addition, we collected 15 datasets with 2965 patients to train and validate our ISTMEscore system, and depicted the landscapes of immune and stromal cell infiltration, transcriptome, genome, prognosis and immunotherapy responses in patients with different ISTME scores. Finally, we compared ISTMEscore with previous TME indexes on prediction of TME status and cancer prognosis.

## Methods

### Data collection and processing

#### Transcriptomic cohorts

We collected 15 transcriptomic cohorts for LUAD, SKCM and HNSC with clinical annotations from The Cancer Genome Atlas (TCGA) [11], Gene Expression Omnibus (GEO) [12–17] and PubMed [5]. Their basic information was presented in Additional file 9: Table S1. Samples without prognostic information were excluded. The normalized data of 3 TCGA cohorts (TCGA LUAD, TCGA SKCM and TCGA HNSC) were downloaded from Firehose RSEM files [18]. The microarray data from GEO were processed with RMA standardization, except that NanoString nCounter data were processed using Housekeeper genes [19]. All transcriptomic data were normalized by Z-score and min–max for further analysis [20]. R sva package was used for batch effect correction [21].

#### Mutation and copy number alteration data

The TCGA somatic mutations were called by Mutect2 [22]. Only non-silent mutations were included in this study. Copy number alteration (CNA) of the TCGA cohorts was detected by Affymetrix SNP 6.0 array and GISTIC2 after germline subtraction [23].

#### Immunotherapy cohorts

A total of 5 independent transcriptomic cohorts containing patients with immune checkpoint inhibitor (ICI) treatment were used to validate the ISTMEscore system [5, 24–27]. The detailed information of these immunotherapy cohorts was presented in Additional file 10: Table S2. The immunotherapy dataset 1 [5] included 28 melanoma patients before and after anti-PD-1/CTLA4 therapies. The immunotherapy dataset 2 (GSE91061) [24] included 65 melanoma patients before and after anti-PD-1 therapies. The immunotherapy dataset 3 (GSE93157) [25] included 35 NSCLC, 5 HNSC and 25 melanoma patients before anti-PD-1 therapy. The immunotherapy dataset 4 (GSE67501) [26] included 11 renal cell carcinoma patients before anti-PD-1 therapy. The immunotherapy dataset 5 (GSE35640) [27] included 56 melanoma patients before anti-MAGE-A3 therapy.

### Development of novel immune and stromal scores

#### Step 1: Extraction of low dimensional feature associated with TME signals via non-negative matrix factorization

Non-negative matrix factorization (NMF) was an unsupervised algorithm for low dimensional feature extraction, which was performed by R NMF package based on brunet methods [28]. The specific formula was as follows:

$$\mathrm{V}{\in {R}_{+}}^{i*j};\mathrm{w}{\in {R}_{+}}^{i*k};\mathrm{H}{\in {R}_{+}}^{k*j}$$

$${\mathrm{V}}_{i*j}\approx {\mathrm{W}}_{i*k}*{\mathrm{H}}_{k*j}$$

where ${\mathrm{V}}_{i*j}$ is gene expression matrix with i gene and j sample, ${\mathrm{W}}_{i*k}$ represents basis matrix with i gene and k the low dimensional features (LDF). ${\mathrm{H}}_{k*j}$ is the coefficient matrix considered as a low dimensional matrix of ${\mathrm{V}}_{i*j}$. The k means the number of NMF clusters. The j sample and i gene can be divided into the k clusters, respectively:

$${\mathrm{sample}\_\mathrm{cluster}}_{j}=k, \quad when\; {\mathrm{H}}_{k*j}=Max({\mathrm{H}}_{j})$$

$${\mathrm{gene}\_\mathrm{cluster}}_{i}=k, \quad when\; {\mathrm{W}}_{i*k}=Max({\mathrm{W}}_{i})$$

The number of NMF clusters (k) was determined by hierarchical clustering of gene expression matrix. Normally, $\mathrm{k}*\left(\mathrm{i}+\mathrm{j}\right)<i*j$, thus high dimensional genes in transcriptome data (i gene) were transformed into low dimensional eigenvalues (k feature).

In this study, we first defined k as 11 through hierarchical clustering of ${\mathrm{V}}_{i*j}$ from the training datasets. Next, enrichment analysis was performed based on genes with high NMF weights (top 100) to identify clusters associated with immune and stromal signals. In addition, we extracted eigenvalues (${\mathrm{H}}_{k}$) from each cluster by NMF, and used univariate Cox regression to identify prognosis-related clusters. Finally, we identified one immune- (${k}_{immu}$-th) and one stromal-related clusters (${k}_{stro}$-th), both of which were associated with immune/stromal signals and the overall survival (OS).

#### Step 2: Identification of TME-related signatures with the ℓ2,1-norm multitask learning linear model

Multitask learning (MTL) was an ensemble approach, which can train multiple tasks at the same time. By introducing ℓ2,1 regularization term into cost function, the output of regression coefficient matrix was sparse. The cost function of the MTL model was based on least squares loss:

$$\sum_{t=1}^{m}||{W}_{t}^{T}{X}_{t}-{Y}_{t}|{|}_{F}^{2}+{\rho }_{1}{||W||}_{\mathrm{2,1}}+{\rho }_{L2}{|\left|W\right||}_{F}^{2}$$

$${||W||}_{\mathrm{2,1}}=\sum_{i=1}^{n}\sqrt{\sum_{j=1}^{m}{W}_{i,j}^{2}}$$

$${|\left|W\right||}_{F}=\sqrt{\sum_{i=1}^{n}\sum_{j=1}^{m}{W}_{i,j}^{2}}$$

where ${W}_{t}$ denotes the coefficient matrix of multi-factor linear regression model in the task t, ${X}_{t}$ is the gene expression matrix (${\mathrm{V}}_{i*j}$ in NMF) of the task t, ${Y}_{t}$ is sample label (immune or stromal scores), ${||W||}_{\mathrm{2,1}}$ denotes ℓ2,1-norm term, and ${|\left|W\right||}_{F}$ denotes Frobenius-norm term. The accelerated gradient methods were used to minimize the cost function. Compared to Lasso or Ridge, the ℓ2,1-norm regularization resulted in grouped sparsity across sub-tasks. Thus, we selected the small subset of genes with the effective information in the input ${\mathrm{V}}_{i*j}$ matrix. The MTL code was based on MATLAB from the previous studies [29].

In this study, there were 2 sub-tasks (t = 2), and ${Y}_{t}$ was defined as follow:

$${Y}_{t}=\left[{Y}_{1},{Y}_{2}\right]=\left[{H}_{{k}_{immu}},{H}_{{k}_{stro}}\right]$$

where ${H}_{{k}_{immu}}$ is ${k}_{immu}$-th column of coefficient matrix of NMF, and ${H}_{{k}_{stro}}$ is ${k}_{stro}$-th column of coefficient matrix of NMF. Moreover, addition of ℓ2,1 regularization term contributed to avoid impact of multicollinearity among genes. Through ℓ2,1-norm MTL, we identified the genes associated with TME-related LDF (criterion: the coefficient of each gene in coefficient matrix ${W}_{t}$ $\ne$ 0).

#### Step 3: Optimization of the gene list through different gene expression analysis and consensus clustering

Consensus clustering (number of clusters = 2) for MTL genes was performed to divide the TCGA training datasets into 2 clusters through NMFConsensus (GenePattern module) [30]. Different gene expression (DGE) analysis was then used to identify differentially expressed genes in these 2 consensus clusters by R limma package [31]. Benjamini-Hochberg (BH) method was used for multiple hypothesis adjustment of DGE [32]. To verify whether the consensus clustering was related to TME, enrichment analysis of the genes with top 1,000 positive and top 1,000 negative logFC was performed. According to results of enrichment analysis, we defined the 108 genes from MTL list with P < 0.05 and logFC > 0 as the immune-related genes and the 58 genes with P < 0.05 and logFC < 0 as the stromal-related genes. DGE had 2 effects: (1) Remove the genes not significantly expressed in the consensus clusters; (2) Distinguish immune- and stromal-related genes by the direction of logFC.

#### Step 4: Quantification of novel immune and stromal scores through ssGSEA

The ssGSEA was used to construct novel immune and stromal scores based on immune- and stromal-related genes using the R GSVA package [10]. Compare with the generalized linear model, the normalized ssGSEA score was the enrichment score based on gene rank, and was therefore not sensitive to different platforms (microarray or RNA-seq) or incomplete information of a few genes.

### ISTMEscore R package

We provided an R package named ISTMEscore to calculate our immune and stromal scores, and to estimate TME classification (GitHub: https://github.com/ZengZihang/ISTMEscore).

### Cell-receptor-ligand communication networks

The connections of cells, receptors and ligands were retrieved from the FANTOM5 resource [33]. The edges of interaction networks in each TME subtype were determined by Thorsson’s method [34]. Core subnetworks were determined by the Molecular Complex Detection tool in Cytoscape [35].

### Cell infiltration scores

Cell infiltration was estimated by the MCPcounter [9], containing T cells, CD8+ T cells, cytotoxic lymphocytes, NK cells, B lineages, monocytic lineages, myeloid dendritic cells, neutrophils, endothelial cells and fibroblasts. Estimation of stromal and immune cells in malignant tumors using expression (ESTIMATE) [8] scores provided the purity of immune and stromal components through ssGSEA.

### Propensity score matching analysis

Propensity score matching (PSM) through 1:1 nearest neighbor matching with caliper was performed to adjust clinical confounders. Sample pairs with similar propensity scores (calipers = 0.05) were matched one-to-one in different groups to make the baseline levels consistent. PSM was performed by R MatchIt package [36].

### Gene enrichment analysis

Gene set enrichment and over-representation analysis were performed by R clusterProfiler packages based on Gene Ontology (GO) corpus [37].

### Decision curve analysis

Decision curve analysis (DCA) evaluated the clinical net benefit of risk prediction model using the R rmda package [38].

### Statistical analysis

All statistical analysis was implemented by R software 3.6.1. R survival package was used to perform Cox proportional hazards regression, Kaplan–Meier analysis and calculation of concordance index (C-index) [39]. R stats package was used to perform Pearson, Spearman correlation and chi square test. The receiver operating characteristic (ROC) curve analysis was performed by R pROC package [40]. P value less than 0.05 was considered as statistically significant. All the P values were two-sided. The significant threshold of false discovery rate (FDR) was 0.05 in enrichment analysis.

### Additional analysis

To explore the possible pathways of the stromal-related genes, we performed additional analysis (Additional file 18). The relevant parts were included in the supplementary materials.

## Results

### Feature extraction of TME signals and construction of ISTMEscore

**Fig. 1:**
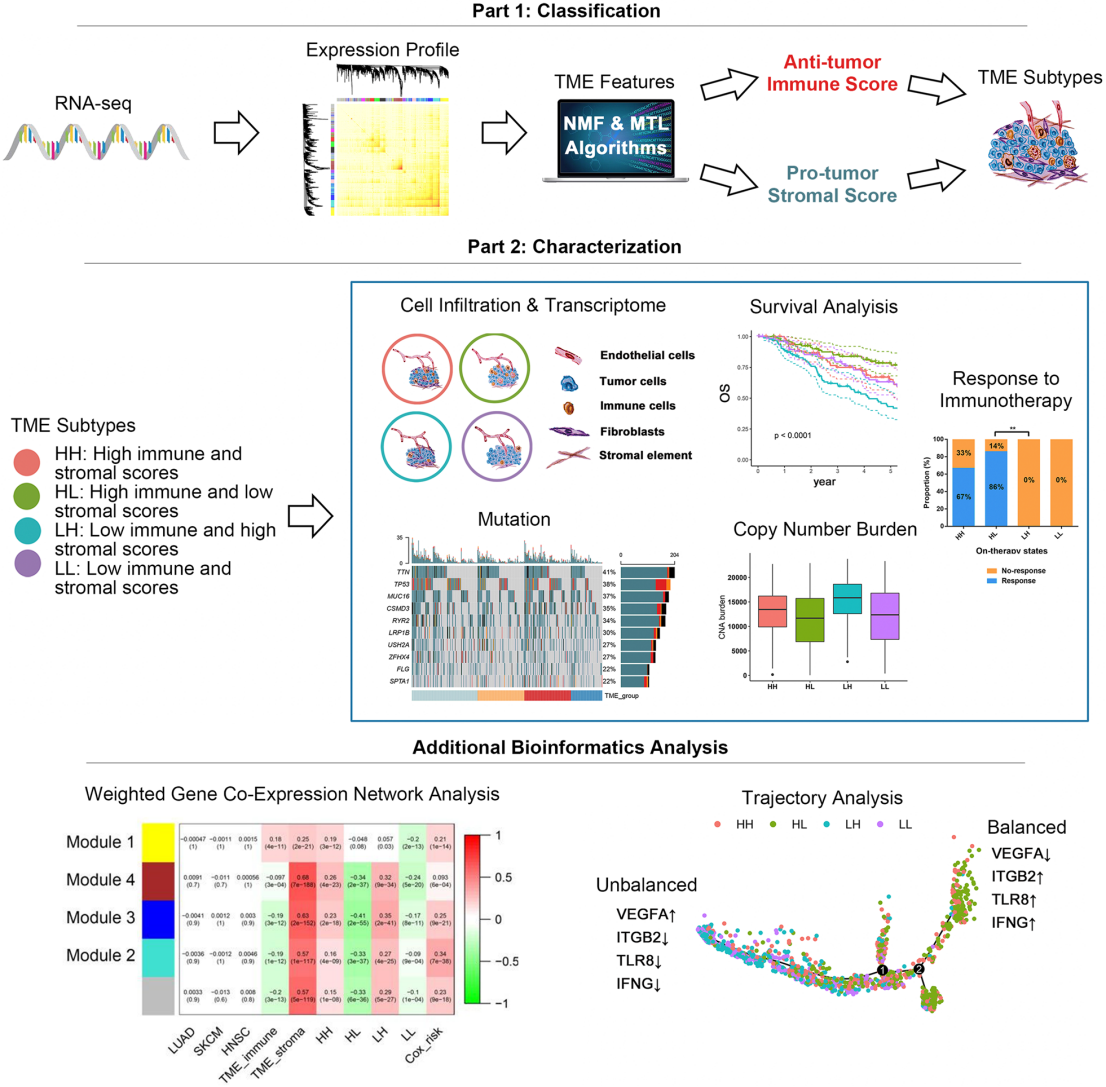
The design of this study. TME, tumor microenvironment

**Fig. 2:**
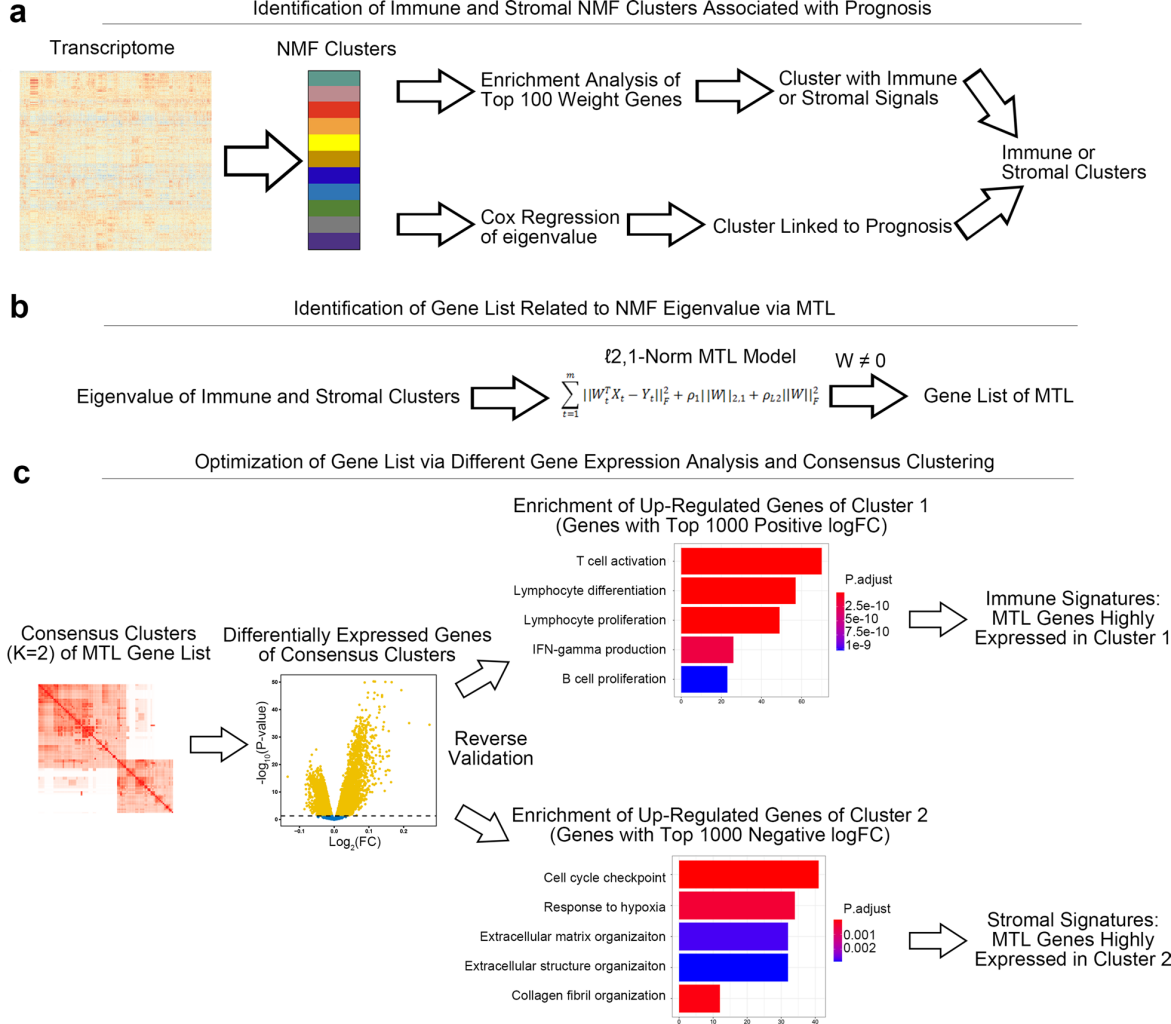
The road map of identification of immune and stromal signatures. **a** Identification of immune and stromal NMF clusters associated with prognosis. **b** Identification of gene list related to NMF eigenvalue via MTL. **c** Optimization of gene list via different gene expression analysis and consensus clustering. NMF, Non-negative matrix factorization; MTL, multitask learning

**Fig. 3:**
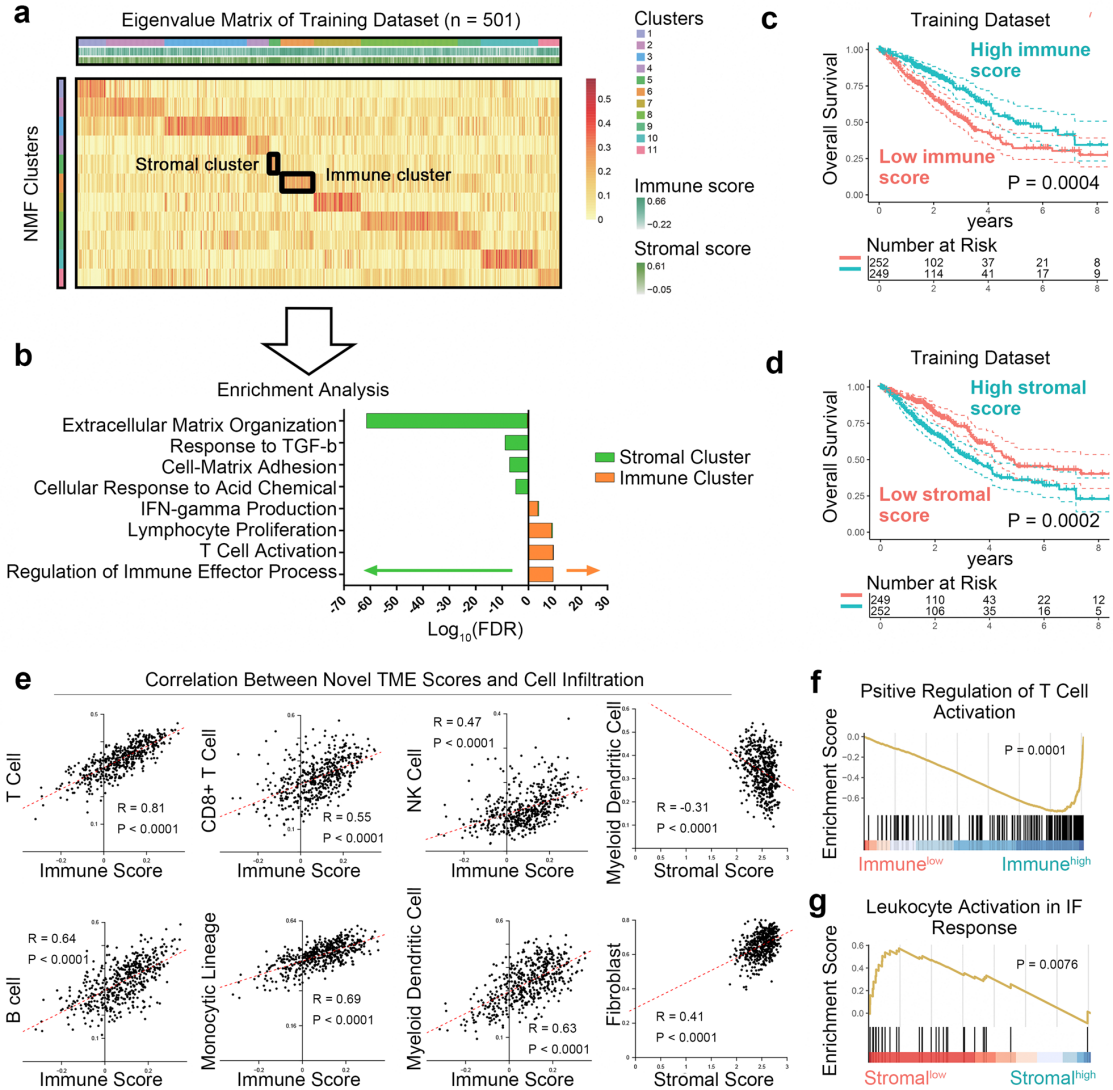
Characteristics of our immune and stromal scores. **a** Eigenvalue matrix in training dataset TCGA LUAD (n = 501). **b** GO enrichment analysis of top 100 weight genes of immune and stromal related clusters. All FDR < 0.05. **c** The immune score was linked to favorable OS in training dataset (Log-rank test). **d** The stromal scores were linked to unfavorable OS in the training datasets (Log-rank test). **e** Correlation Between Novel TME Scores and cell infiltration (Spearman's correlation). **f** GSEA of differently expressed genes in patients with high-low immune scores (Up 50% vs. Low 50%). **g** GSEA of differently expressed genes in patients with high-low stromal scores (Up 50% vs. Low 50%). NMF, Non-negative matrix factorization; TME, tumor microenvironment; IF, inflammation; GSEA, gene set enrichment analysis; OS, overall survival; FDR, false discover rate

The designs of our studies were demonstrated in Figs. 1, 2 and Additional file 1: Figure S1. To isolated the TME-related signals from mixed tumor tissues, we first divided RNA-seq data of the training cohort (TCGA LUAD) [11] into 11 heterogeneous clusters based on NMF (Fig. 3A). Enrichment analysis of clusters' high NMF weight genes (top 100) was performed to identify clusters with immune or stromal signals. Only one cluster was linked with T cell activation and IFN-γ production (Additional file 11: Table S3, Fig. 3B). We extracted the LDF of this cluster by NMF. Univariate Cox regression indicated that the LDF was a favorable prognostic factor (Cox-P < 0.0001). Therefore, this cluster was defined as "immune activation cluster". On the other hand, extracellular matrix organization, cell–matrix adhesion and TGF-β signal were enriched in another cluster (Additional file 12: Table S4, Fig. 3B), and its LDF was identified as an adverse prognostic factor (Cox-P < 0.0001), suggesting this cluster was a "stromal activation cluster". Enrichment results of other 9 clusters were displayed in Additional file 13: Table S5.

We next identified gene signatures from the immune and stromal clusters using our novel workflow (Fig. 2, see “Methods”—“Development of novel immune and stromal scores” for details). A total of 166 genes were identified as TME-related signatures, containing 108 immune- (e.g. CD40LG, CD8A, IFNG, PTPRC, CXCL10) and 58 stromal-related genes (e.g. MMP13, FN1, COL1A1, COL1A2, COL11A1, COL3A1, VEGFA, Additional file 14: Table S6). Novel immune and stromal scores were calculated by immune- and stromal-related signatures using ssGSEA.

### ISTMEscore was linked to prognostic, cellular and molecular characteristics

The association of ISTMEscore with prognostic, cellular and molecular patterns were then investigated. Kaplan–Meier curves indicated that our immune scores were significantly associated with better OS, while stromal scores were associated with poorer OS in the training datasets (Fig. 3C, D). We next characterized the association between ISTMEscore and TME cell infiltration (estimated by MCPcounter) in the TCGA LUAD training dataset [11]. All 6 immune cells (T cells, CD8+ T cells, B cells, NK cells, monocytic lineage and myeloid dendritic cells) were positively correlated with the immune scores (Spearman correlation P < 0.0001, Fig. 3E), and the stromal scores were positively correlated with fibroblasts and negatively correlated with myeloid dendritic cells (Spearman correlation P < 0.0001).

Differentially expressed gene analysis was then performed in patients with high-low immune and stromal scores (Up 50% vs. Low 50%). The GSEA of differentially expressed genes suggested that T cell activation was highly enriched in the patients with high immune scores, while leukocyte activation was less enriched in the patients with high stromal scores (Fig. 3F, G).

The correlation between T cell co-stimulatory/inhibitory molecules and our scores were subsequently analyzed. The stromal scores were negatively correlated with CD28 and CD40LG, and positively correlated with PD-1 (Additional file 2: Figure S2), suggesting that the stromal scores were positively associated with exhaustive immunity.

### Identification of TME subtypes by ISTMEscore

To explore the roles of immune and stromal activation in tumors, patients were divided into 4 TME subtypes according to the median values of ISTME scores. The patients with high immune and stromal scores were considered as the “HH type”, and high immune and low stromal scores were identified as the “HL type”. The “LH type” patients had low immune and high stromal scores, and the patients with low immune and stromal scores were defined as the “LL type”.

### Comprehensive analysis of multi-dimensional landscape of the 4 TME subtypes in LUAD, SKCM and HNSC

#### The landscape of TME cell infiltration in different TME subtypes

**Fig. 4:**
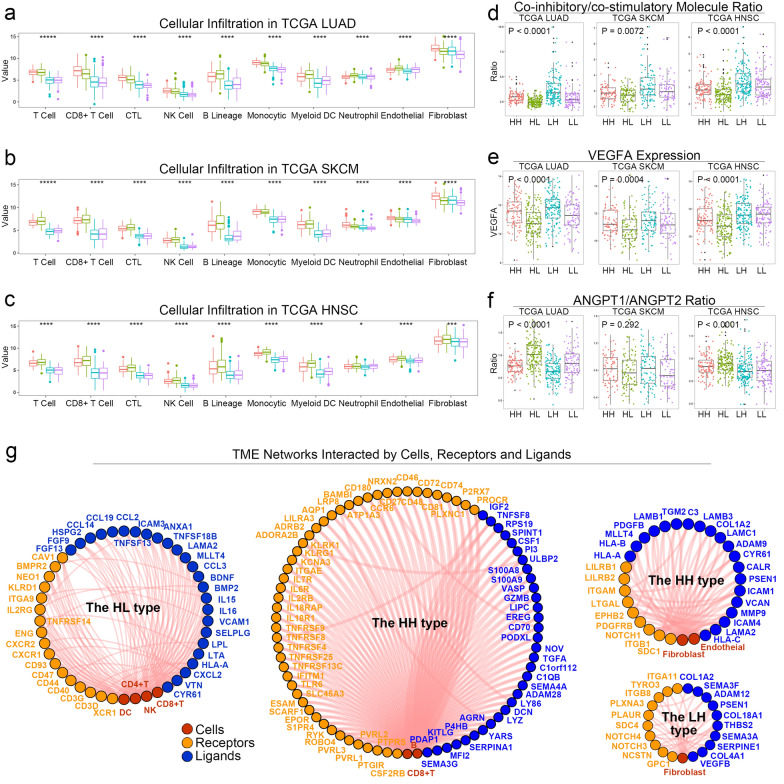
The TME subtype was associated with TME cellular infiltration, functional markers and communication networks. **a** The patterns of cellular infiltration of TME subtypes in TCGA LUAD (n = 501). **b** The patterns of cellular infiltration of TME subtypes in TCGA SKCM (n = 352). **c** The patterns of cellular infiltration of TME subtypes in TCGA HNSC (n = 514). **d** The ratios of co-inhibitory/co-stimulatory molecules (PD-1 + CTLA4 + LAG3 + TIM-3) / (CD28 + CD40LG) of TME subtypes in TCGA LUAD (n = 501). **e** The ratios of co-inhibitory/co-stimulatory molecules of TME subtypes in TCGA SKCM (n = 352). **f** The ratios of co-inhibitory/co-stimulatory molecules of TME subtypes in TCGA HNSC (n = 514). **g** TME communication networks of different TME subtypes in the training datasets. LUAD, lung adenocarcinoma; SKCM, skin cutaneous melanoma; HNSC, head and neck squamous cell carcinoma; TME, tumor microenvironment; HH, immune^high^ stromal^high^; HL, immune^high^ stromal^low^; LH, immune^low^ stromal^high^; LL, immune^low^ stromal^low^

To depict the TME immune landscape of the 4 subtypes, the infiltration of immune cells was calculated using MCPcounter. In the TCGA LUAD, SKCM and HNSC cohorts, the HH and HL patients had significantly high levels of immune cell infiltration, including T cells, CD8+ T cells, cytotoxic lymphocytes, NK cells, B lineages, monocytic lineages and myeloid dendritic cells (Fig. 4A–C). We further assessed the cellular patterns of TME subtypes in other independent cohorts, containing GSE11969 (NSCLC, n = 149) [12], GSE68465 (LUAD, n = 442) [13], GSE68571 (LUAD, n = 86) [14], GSE37745 (NSCLC, n = 196) [15], GSE50081 (NSCLC, n = 172) [16] and GSE65904 (SKCM, n = 214) [17]. Consistent with the outcomes in the TCGA cohorts, the HH and HL patients also had high immune cell infiltration (Additional file 3: Figure S3), except GSE68571, due to the lack of mapping genes.

#### TME subtype was associated with the functional markers of T cells and angiogenesis

Downregulation of co-stimulatory molecules (CD28, CD40LG) and upregulation of co-inhibitory molecules (PD-1, CTLA4, LAG3, TIM-3) were detected in the exhaustive T cells. In the TCGA LUAD dataset, the ratio of co-inhibitory/co-stimulatory molecules (PD-1 + CTLA4 + LAG3 + TIM-3)/(CD28 + CD40LG) was significantly different in the 4 TME subtypes (analysis of variance, ANOVA, P < 0.0001, Fig. 4D). Contrary to the LH patients, the HL patients had the lowest ratio. Similar patterns were also found in the TCGA SKCM (ANOVA, P = 0.0072) and TCGA HNSC datasets (ANOVA, P < 0.0001).

In addition, angiogenic markers, VEGFA levels and ANGPT1/ANGPT2 ratios had significantly different levels among TME subtypes in the TCGA LUAD and TCGA HNSC datasets (ANOVA, P < 0.0001, Fig. 4E, F). The HL patients had low levels of VEGFA and high ANGPT1/ANGPT2 ratios, suggesting inhibited angiogenesis. VEGFA levels were associated with TME subtypes in the TCGA SKCM dataset (ANOVA, P = 0.0004).

#### TME subtype was associated with distinct TME networks composed of cells, receptors and ligands

In TME, signal transmission between cells was mediated by secreted proteins and direct communications. The TME communication networks were established to characterize cell interactions, as well as receptors and ligands, with Thorsson's method in the training datasets (Fig. 4G) [34]. The TME networks of the HH type composed of CD8+ T cells, B cells, NK cells and fibroblasts as the center of the subnetworks mediated by integrins and chemokines. The immune cell-centered network was identified in the HL type. The fibroblast-centered networks were observed in the LH type. However, no network was identified in the LL type, suggesting its desert-like TME.

#### TME subtype was linked to clinicopathological characteristics

According to the above findings, our TME subtypes could represent TME patterns and were associated with immune regulation in LUAD, SKCM and HNSC. The correlations between TME subtypes and clinicopathological characteristics were next analyzed. In the TCGA LUAD dataset, the LH type was concentrated on young patients at pathologic N2–3 and stage III–IV (Table 1), which were related to higher malignancy and poorer survival. In the TCGA HNSC dataset, the LH type was significantly enriched in male patients at pathologic T3–4, which was contrary to HL. In the TCGA SKCM dataset, TME subtype was independent of clinicopathological characteristics.

**Table 1:** The clinical characteristics of the 4 subgroups in TCGA LUAD, TCGA SKCM and TCGA HNSC

Variable	TCGA LUAD					TCGA SKCM					TCGA HNSC				
Subtypes	HH	HL	LH	LL	*P*	HH	HL	LH	LL	*P*	HH	HL	LH	LL	*P*
Gender (%)/Chi-squared test					0.35					0.14					0.0016**
Male	41 (43.2)	66 (42.6)	75 (48.4)	51 (53.1)		47 (58.0)	53 (55.8)	66 (69.5)	55 (67.9)		76 (75.2)	102 (65.4)	131 (84.0)	70 (69.3)	
Female	54 (56.8)	89 (57.4)	80 (51.6)	45 (46.9)		34 (42.0)	42 (44.2)	29 (30.5)	26 (32.1)		25 (24.8)	54 (34.6)	25 (16.0)	31 (30.7)	
Median age (IQR)/ANOVA	66.5 (57.7–72.2)	69.4 (62.7–75.0)	63.3 (56.2–71.6)	65.3 (60.5–72.0)	< 0.0001****	56.7 (46.4–64.8)	56.3 (45.0–71.6)	56.1 (49.0–68.7)	60.0 (46.4–70.9)	0.71	60.77 (53.51–68.99)	61.11 (55.03–69.86)	60.85 (53.03–67.88)	61.72 (53.04–69.02)	0.72
Pathologic T (%)/Chi-squared test					0.16					0.068					0.018*
T0–2	86 (90.5)	139 (90.8)	129 (83.8)	81 (84.4)		38 (58.5)	39 (52.0)	35 (45.5)	24 (36.4)		37 (41.1)	64 (48.5)	41 (30.0)	40 (43.0)	
T3–4	9 (9.5)	14 (9.2)	25 (16.2)	15 (15.6)		27 (41.5)	36 (48.0)	42 (54.5)	42 (63.6)		53 (58.9)	68 (51.5)	96 (70.0)	53 (57.0)	
Pathologic N (%)/Chi-squared test					< 0.0001****					0.3					0.42
N0–1	81 (85.3)	139 (93.9)	115 (75.2)	83 (88.3)		50 (68.5)	66 (77.6)	59 (68.6)	55 (78.6)		40 (49.4)	71 (60.2)	77 (58.8)	51 (60.0)	
N2–3	14 (14.7)	9 (6.1)	38 (24.8)	11 (11.7)		23 (31.5)	19 (22.4)	27 (31.4)	15 (21.4)		41 (50.6)	47 (39.8)	54 (41.2)	34 (40.0)	
Pathologic M (%)/Fisher's test					0.3					0.27					0.16
M0	69 (94.5)	95 (96.0)	105 (92.1)	63 (88.7)		70 (93.3)	86 (96.6)	87 (94.6)	64 (88.9)		40 (100)	56 (100)	57 (100)	29 (96.7)	
M1	4 (5.5)	4 (4.0)	9 (7.9)	8 (11.3)		5 (6.7)	3 (3.4)	5 (5.4)	8 (11.1)		0	0	0	1 (3.3)	
Pathologic stage (%)/Chi-squared test					< 0.0001****					0.38					0.16
Stage I	47 (49.5)	106 (69.3)	64 (41.3)	54 (56.3)		19 (25.7)	24 (28.9)	18 (19.8)	14 (19.4)		2 (2.2)	11 (8.5)	5 (3.7)	9 (9.9)	
Stage II	28 (29.5)	31 (20.3)	43 (27.7)	19 (19.8)		12 (16.2)	19 (22.9)	29 (31.9)	23 (31.9)		13 (14.6)	28 (21.7)	17 (12.6)	15 (16.5)	
Stage III	16 (16.8)	11 (7.2)	39 (25.2)	14 (14.6)		38 (51.4)	37 (44.6)	39 (42.9)	28 (38.9)		18 (20.2)	20 (15.5)	24 (17.8)	17 (18.7)	
Stage IV	4 (4.2)	5 (3.3)	9 (5.8)	9 (9.4)		5 (6.8)	3 (3.6)	5 (5.5)	7 (9.7)		56 (62.9)	70 (54.3)	89 (65.9)	50 (54.9)	
Survival rate (%)/log-rank					< 0.0001****					< 0.0001****					0.39
3-year OS	60.7	78.4	45.5	69.2		75	85.1	60.8	79.4		56.2	65.6	52.7	54.4	
5-year OS	41.2	55	30.2	40.1		65.1	78.4	43.2	63.3		46.2	50.3	47	47.4	

*LUAD* lung adenocarcinoma, *SKCM* skin cutaneous melanoma, *HNSC* head and neck squamous cell carcinoma, *NS* not significant

* < 0.05; ** < 0.01; *** < 0.001; **** < 0.0001

#### TME subtype was an independent prognostic factor

**Fig. 5:**
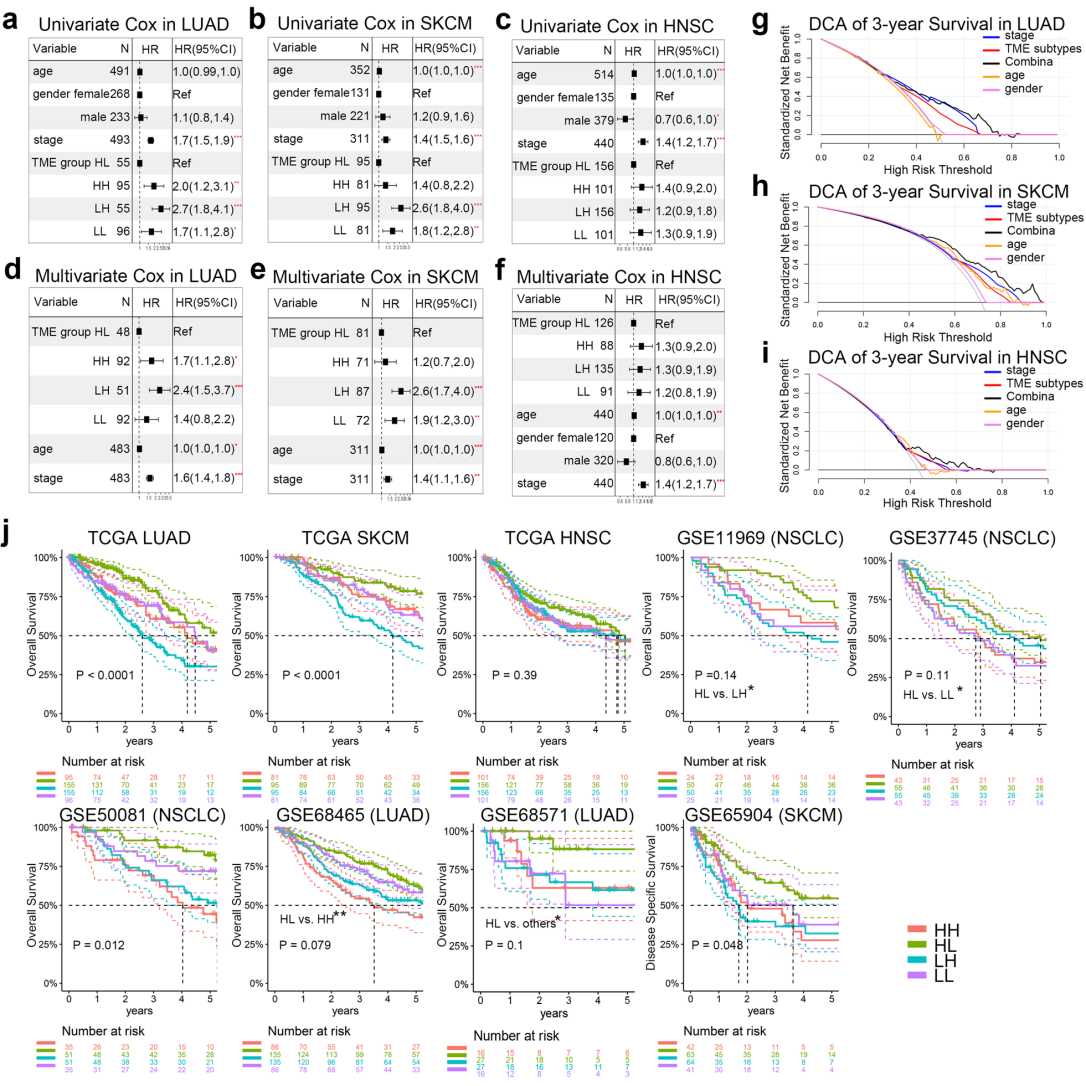
The TME subtype was an independent prognosis factor in LUAD, SKCM and HNSC. **a** Univariate Cox regression of the 4 prognostic features (age, gender, Pathologic stage and TME subtype) in TCGA LUAD. **b** Univariate Cox regression of the 4 prognostic features (age, gender, pathologic stage and TME subtype) in TCGA SKCM. **c** Univariate Cox regression of the 4 prognostic features (age, gender, pathologic stage and TME subtype) in TCGA HNSC. **d** Multivariate Cox regression of prognostic features in TCGA LUAD. **e** Multivariate Cox regression of prognostic features in TCGA SKCM. **f** Multivariate Cox regression of prognostic features in TCGA HNSC. **g** DCA of 3-year prediction in TCGA LUAD. **h** DCA of 3-year prediction in TCGA SKCM. **i** DCA of 3-year prediction in TCGA HNSC. **j** The Kaplan–Meier curves of the 4 subtypes in the training and validation cohorts, including TCGA cohorts, GSE68465 (LUAD, n = 442), GSE68571 (LUAD, n = 86), GSE50081 (NSCLC, n = 172), GSE37745 (NSCLC, n = 196), GSE11969 (NSCLC, n = 149), GSE65904 (SKCM, n = 214). DCA, decision curve analysis; LUAD, lung adenocarcinoma; SKCM, skin cutaneous melanoma; HNSC, head and neck squamous cell carcinoma; TME, tumor microenvironment; HH, immune^high^ stromal^high^; HL, immune^high^ stromal^low^; LH, immune^low^ stromal^high^; LL, immune^low^ stromal^low^

The prognostic features (age, gender, pathologic stage and TME subtype) were identified by clinical value and univariate Cox regression (Fig. 5A–C). Pathological T, N and M have collinearity with pathologic stage, thus we only included the pathologic stages in the Cox model. In the TCGA LUAD dataset, multivariate Cox regression analysis indicated that age (HR = 1.02, P = 0.03), pathologic stage (HR = 1.58, P < 0.0001) and TME subtype (HL vs. LH, HR = 0.42, P < 0.0001) were independently prognostic factors (Fig. 5D). In the TCGA SKCM dataset, age (HR = 1.02, P = 0.0006), pathologic stage (HR = 1.36, P = 0.002) and TME subgroup (HL vs. LH, HR = 0.39, P < 0.0001) were independently prognostic factors (Fig. 5E). In the TCGA HNSC dataset, age (HR = 1.02, P = 0.006), gender (male vs. female, HR = 0.76, P = 0.1), pathologic stage (HR = 1.45, P < 0.001) and TME subgroup (HL vs. HH, HR = 0.74, P = 0.168) were independently prognostic factors (Fig. 5F). Combining the 4 prognostic factors using Cox model, DCA revealed that the inclusion of TME subtype had higher potential clinical utility (Fig. 5G–I).

In the Kaplan–Meier curves (Fig. 5J), the HL patients had the best OS in the TCGA LUAD (logrank-P < 0.0001), SKCM (logrank-P < 0.0001) and HNSC (logrank-P = 0.39) datasets. To adjust the impacts of prognostic covariates (age, gender and pathologic stage) on prognosis of TME subtypes, we performed PSM to baseline correction. In the TCGA LUAD dataset, the HL type had significantly favorable OS, while the LH type had unfavorable OS (logrank-P = 0.043, Additional file 4: Figure S4A&B) after PSM (For all subtypes: age, ANOVA, P = 0.986; gender, Chi-squared test, P = 0.418; stage, Chi-squared test, P = 0.228). In the TCGA SKCM dataset, the survival curve was similar to that of the TCGA LUAD dataset (logrank-P = 0.00061, Additional file 4: Figure S4C&D) after PSM (For all subtypes: age, ANOVA, P = 0.882; gender, Chi-squared test, P = 0.43; stage, Chi-squared test, P = 0.796). In the TCGA HNSC dataset, survival curve also showed difference (logrank-P = 0.096, HL vs. LH: P = 0.03, Additional file 4: Figure S4E&F) after PSM (For all subtypes: age, ANOVA, P = 0.622; gender, Chi-squared test, P = 0.931; stage, Chi-squared test, P = 0.471).

To validate our TME subtypes in the other 6 independent datasets [12–17], we performed Kaplan–Meier analysis in each dataset. The HL patients were associated with significantly favorable prognosis in all validation datasets (Fig. 5J).

#### TME subtype was associated with mutation profile and copy number alteration

**Fig. 6:**
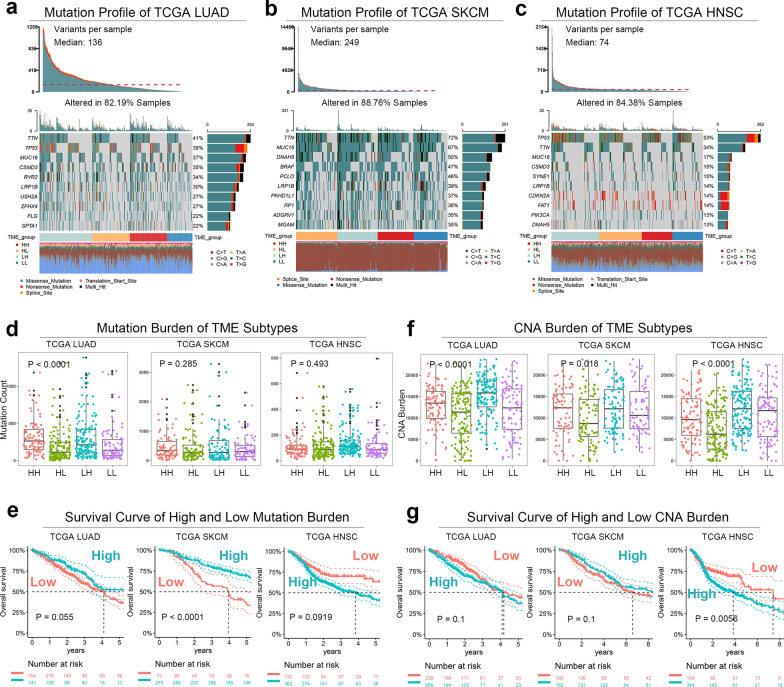
The TME subtype was associated with mutation profile and copy number alteration. **a** Mutation profile of TCGA LUAD. **b** Mutation profile of TCGA SKCM. **c** Mutation profile of TCGA HNSC. **d** Mutation count of different TME subtypes in the 3 TCGA cohorts. **e** The Kaplan–Meier curves of patients with high-low mutation count in the 3 TCGA cohorts. **f** Copy number alteration count burden of different TME subtypes in the 3 TCGA cohorts. **g** The Kaplan–Meier curves of patients with high-low copy number alteration count burden in the 3 TCGA cohorts. LUAD, lung adenocarcinoma; SKCM, skin cutaneous melanoma; HNSC, head and neck squamous cell carcinoma; TME, tumor microenvironment; HH, immune^high^ stromal^high^; HL, immune^high^ stromal^low^; LH, immune^low^ stromal^high^; LL, immune^low^ stromal^low^

To determine whether TME subtypes were driven by gene mutations, we analyzed the somatic mutation profile in the 3 cancers from TCGA (Fig. 6A–C). Gene mutations in the 4 TME subtypes were compared using Fisher's test (Additional file 5: Figure S5A–F). However, there was no duplicated differentially mutated genes in the 3 cancers. The mutation frequencies of traditional driving genes were then compared (Additional file 6: Figure S6A–I). In LUAD and HNSC, the mutation frequency of TP53 was significantly downregulated in the HL type, and the mutation sites of TP53 were concentrated at DNA binding domains (Additional file 7: Figure S7A&B). In SKCM, there were no differently mutated driver genes in the 4 TME subtypes.

Despite the controversy, tumor mutation burden (TMB) was the novel biomarker for ICI therapy according to CheckMate-032 (SCLC) and CheckMate-026 (NSCLC) [41, 42], and its roles in prognosis of patients without ICIs remained unclear. The non-silent mutation counts in the exon region represent relative values of TMB [43]. In this study, the HH and LH types were associated with high non-silent mutation count (ANOVA P < 0.0001, Fig. 6D), which was linked to better OS (logrank-P = 0.055, Fig. 6E) in the TCGA LUAD dataset. In the TCGA SKCM and HNSC datasets, the TME subtype had no significant mutation count that was also linked to prognosis.

The association between CNA burden and prognosis was still unclear. Here, we defined CNA count burden (CNACB) as the total gene counts with CNA in each sample. In the 3 TCGA cohorts, the HL type had lowest CNACB (Fig. 6F), which was an unfavorable prognostic factor in HNSC (logrank-P = 0.0056) and LUAD (logrank-P = 0.1), but a favorable factor in SKCM (logrank-P = 0.1, Fig. 6G).

### TME subtype was related to ICI responses

**Fig. 7:**
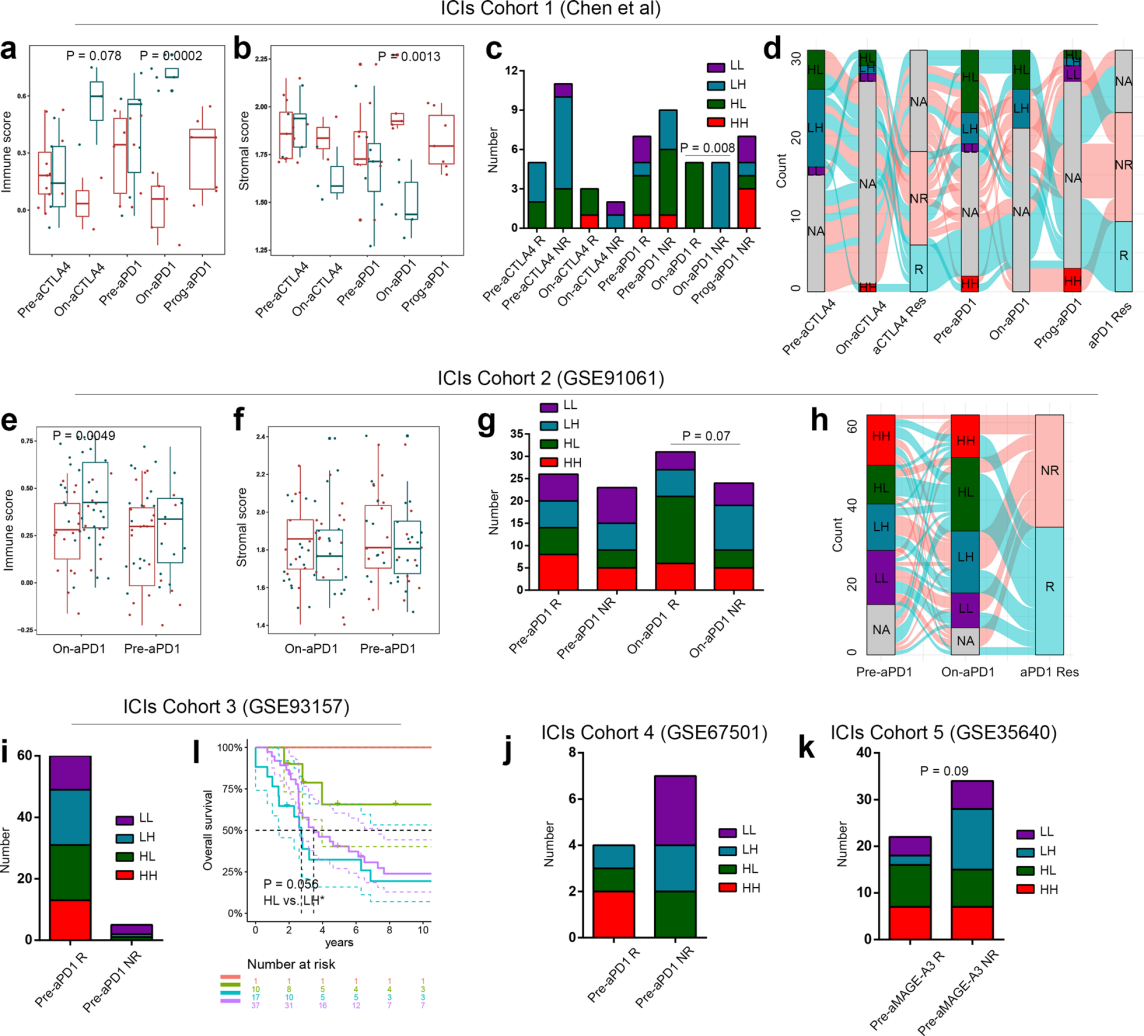
TME subtype could predict response to ICIs. **a** The levels of immune score in different biopsy times (Chen et al.). **b** The stromal scores in different biopsy times (Chen et al*.*). **c** ICI responses of different TME subtypes (Chen et al*.*). **d** Sankey diagram revealed the subtype changes of patients at different time points (Chen et al*.*). **e** The immune scores in different biopsy times (GSE91061). **f** The stromal scores in different biopsy times (GSE91061). **g** ICI responses of different TME subtypes (GSE91061). **h** Sankey diagram revealed the subtype changes of patients at different time points (GSE91061). **i** ICI responses of different TME subtypes (GSE93157). **j** ICI responses of different TME subtypes (GSE67501). **k** ICI responses of different TME subtypes (GSE35640). **l** TME subtype was linked to PFS of patients with ICI treatment in GSE93157. ICI, immune checkpoint inhibitor; LUAD, lung adenocarcinoma; SKCM, skin cutaneous melanoma; HNSC, head and neck squamous cell carcinoma; TME, tumor microenvironment; HH, immune^high^ stromal^high^; HL, immune^high^ stromal^low^; LH, immune^low^ stromal^high^; LL, immune^low^ stromal^low^

Five independent transcriptomic cohorts containing patients with immunotherapy were used to validate the predictive effects of our TME subtype on ICI responses. In the immunotherapy dataset 1 (Chen et al*.*) of 53 melanoma cases [5], there were 5 biopsy timepoints: pre-anti-CTLA4, on-anti-CTLA4, pre-anti-PD-1, on-anti-PD-1 and prog-anti-PD-1. Immune scores were markedly increased in on-anti-CTLA4 (P = 0.078) and on-anti-PD-1 (P = 0.0002) responders (Fig. 7A), while stromal scores were decreased in on-anti-PD-1 therapy (P = 0.0013) responders (Fig. 7B). The HL patients showed higher responses than the LH patients with on-anti-CTLA4/PD-1 therapy (HH vs. HL vs. LH vs. LL: 100% vs. 100% vs. 0% vs. 0%, Fisher's test P = 0.0012, Fig. 7C). Notably, 41.9% patients experienced transition of TME subtype at the 5 biopsy timepoints (Fig. 7D). There were 8 patients with changes from the LH and LL types before anti-CTLA4 therapy to HH and HL types at later times, and 75% patients were responsive.

The immunotherapy dataset 2 (GSE91061) included 65 melanoma patients with anti-PD-1 therapy [24]. Similar to immunotherapy dataset 1, immune scores were significantly higher in responders with on-anti-PD-1 therapy (P = 0.0049, Fig. 7E), but stromal scores were not significantly lower (Fig. 7F). The LH patients were linked to low responses of on-anti-PD-1 therapy (HH vs. HL vs. LH vs. LL: 54.5% vs. 78.9% vs. 37.5% vs. 44.4%, Fisher's test P = 0.07, Fig. 7G). Moreover, 29% patients experienced transition of TME subtype before and during treatment (Fig. 7H). Furthermore, 66.7% patients, who changed to LH and LL types, were not responsive, while 66.7% patients, who changed to HH and HL types, were responsive.

Other immunotherapy datasets only included transcriptomic data before treatment. There was no statistical significance between TME subtype and immunotherapy response in these datasets (Fig. 7I–L).

### Comparison of ISTMEscore system with other studies

#### The overlap between our TME gene signatures and existing signatures

To test the robustness of this study, we compared our TME signatures (“Results”—“Feature extraction of TME signals and construction of ISTMEscore”) with existing signatures [44–49]. There were 30.6% and 31% overlaps of ISTMEscore signatures with ESTIMATE and MCPcounter, respectively (Additional file 15: Table S7).

#### Comparison with other algorithms for prediction of prognosis and ICI responses

Since ESTIMATE [8] and MCPcounter [9] algorithms also provided TME-related scores, it was necessary to compare these indicators with our ISTMEscore. We first calculated C-index for each score on prognosis prediction in all 9 datasets with prognostic annotation (Additional file 16: Table S8). The top 5 indicators of C-index were our stromal score (mean C-index: 0.587), our immune score (mean rank: 0.58), B lineage infiltration (mean C-index: 0.578), myeloid dendritic cell infiltration (mean C-index: 0.567), ESTIMATE immune score (mean C-index: 0.566). We next applied ROC analysis on ICI responses, and calculated area under receiver (AUC) of ROC in the 5 immunotherapy cohorts (Additional file 16: Table S8). The top 5 indicators of AUC were monocytic lineage infiltration (mean AUC: 0.73462), our immune score (mean AUC: 0.72636), myeloid dendritic cell infiltration (mean AUC: 0.72574), T cell infiltration (mean AUC: 0.72034) and our stromal score (mean AUC: 0.71854).

Moreover, we compared our scores with ESTIMATE scores in GSE9014 [50], which included 111 arrays of stroma through Laser Capture Microdissected from 53 breast cancer patients. Only our stromal scores were significantly associated with the histologic GRADE (Additional file 8: Figure S8A-D, P < 0.0001). Table of abbreviations was displayed in Additional file 17: Table S9.

## Discussion

In this study, we built the novel ISTMEscore system with unique workflow, and depicted the multi-dimensional landscape of different TME subtypes. Our TME subtypes could represent TME patterns, and were associated with clinical features and immunotherapy responses in LUAD, SKCM and HNSC. Additional analysis suggested that high collagen, matrix metalloproteinases, glycolysis and acid environment, VEGF signaling were the integral parts of stromal activation. Whether the stromal signals were involved in immune exhaustion remained to be further studied.

Although immunotherapy benefited LUAD, SKCM and HNSC patients, only a small proportion of patients had long-term survival [5]. Identification of potentially sensitive population for ICIs helped to decrease medical expenses and improve quality of life. Our studies found that the LH type with malignant TME and the LL type with desert-like TME had low ICI responses (Fig. 7). Interestingly, the TME subtypes determined by transcriptome before treatment did not demonstrate significant association with immunotherapy response. In the 3 immunotherapy cohorts with biopsies taken before treatment, TME subtype was not significantly associated with ICI response: HH vs. HL vs. LH vs. LL: 100% vs. 94.7% vs. 94.7% vs. 78.6%, Fisher's test P = 0.2 in GSE93157 (Fig. 7I) [25]; and HH vs. HL vs. LH vs. LL: 100% vs. 33.3% vs. 33.3% vs. 0%, Fisher’s test P = 0.26 in GSE67501 (Fig. 7J) [26]; and HH vs. HL vs. LH vs. LL: 50% vs. 52.9% vs. 13.3% vs. 40%, Fisher’s test P = 0.09 in GSE35640 (Fig. 7K) [27]. In GSE93157, there was a difference of progression-free survival only in subgroup comparisons of HL vs. LH (logrank-P = 0.056, Fig. 7L). Chen et al*.* also found positive implications for dynamic monitoring of the immune microenvironment [5]. The protein levels of CD3, CD4, CD8, PD-1, PD-L1 and LAG3 during the treatment could reflect the responses (all, P < 0.01) better than those before treatment. Moreover, Riaz et al*.* found that patients with TME of immune activation or “hot tumor” were associated with high CR/PR rates in the group receiving ICIs in advance, whereas TME immune infiltration was not linked to ICI responses in the patients without prior immunotherapy [24]. In stromal environment, the desmoplastic stroma was the physical barrier for tumor to resist immunotherapy and chemotherapy [51]. Kim et al*.* reported the single-cell sequencing analysis of the longitudinal samples from 20 triple-negative breast cancer patients during neoadjuvant chemotherapy [52]. They found that degradation of ECM and angiogenesis signals were upregulated in the chemoresistant tumors. In preclinical studies, cancer-associated fibroblasts were reported to compensate immunotherapy through crosstalk with myeloid-derived suppressor cells and CD8+ T cells [53, 54]. In this study, we found that the majority of patients, who switched to the LH and LL types during ICI therapy, were non-responders. Accordingly, longitudinal detection of TME-related indicators might be a better choice for patients with ICI treatment, which required studies with large sample size.

The HL patients had high immune activation, inhibitory angiogenesis and long OS, which was considered as balanced states of TME. As previous studies, there were synergistic effects between immune normalization and vascular normalization [55]. VEGF induced immunosuppressive cells such as myeloid suppressive cells, tumor-related macrophages and Tregs, which developed immune exhaustion. On the other hand, the infiltration and activation of intratumoral effector T cells promoted the remodeling and normalization of vessels. The immune and vascular normalization might explain the results of IMpower150 study, in which the first-line immunotherapy combined with anti-angiogenic drugs benefited non-squamous NSCLC patients [56]. The immune and vascular normalization seemed to correspond to the HL type in our study.

Compared with the existing methods (ESTIMATE and MCPcounter) [8, 9], our scores showed advantages on the prediction of prognosis and immunotherapy response. However, the improvement in predictive performance was not large. B lineage and myeloid dendritic cell infiltration scores of MCPcounter were also good predictors of prognosis (Additional file 16: Table S8). For prediction of ICI responses, monocytic lineage infiltration scores also had excellent performance.

The effects of TMB on prognosis were controversial. Some clinical studies revealed opposite prognostic effects of TMB in NSCLC patients without immunotherapy. In LACE-Bio-II study with 908 NSCLC patients [57], high TMB group (≥ 8 m/Mb) showed better OS, while the low TMB group (< 4 m/Mb) had worse prognosis (P = 0.016). However, another clinical study indicated that higher TMB (≥ 62 m/Mb) was correlated with worse OS in the 90 NSCLC patients (P = 0.0003) [58]. TMB may not be a very robust prognostic marker due to lack of consideration on the threshold, complex effects of mutation, as well as the gene mutations from immune or stromal cells.

There were some limitations in this study: All datasets were retrospective, requiring prospective clinical trials to further verify. Whether our scores worked in other tumors, especially tumors lacking immune cell infiltration, remained to be further investigated. In addition, the link between our ISMEscore system and ICI response was not strong. Our system needed to be applied cautiously for the prediction of immunotherapeutic efficacy.

## Conclusions

In conclusion, we proposed the novel ISTMEscore and TME classification. We comprehensively depicted TME classification associated with the cellular, molecular, TME communication networks, mutation, CNA burden and clinical features of LUAD, SKCM and HNSC. Our TME classification was an independent prognostic factor and associated with immunotherapy responses, which was superior to previous studies.

## Supplementary Information

**Additional file 1: Figure S1.** Illustration of identifying TME-related genes.

**Additional file 2: Figure S2.** The correlation between stromal score and T cell co-stimulatory/suppression molecules.

**Additional file 3: Figure S3.** The cellular infiltration patterns in the 6 GEO validation cohorts.

**Additional file 4: Figure S4.** Multivariate Cox regression and propensity score matching analysis. (A) Multivariate Cox regression of clinical characteristics in TCGA LUAD (n = 501). (B) Survival curve after PSM in TCGA LUAD (n = 501). (C) Multivariate Cox regression of clinical characteristics in TCGA SKCM (n = 352). (D) Survival curve after PSM in TCGA SKCM (n = 352). (E) Multivariate Cox regression of clinical characteristics in TCGA HNSC (n = 514). (F) Survival curve after PSM in TCGA HNSC (n = 514).

**Additional file 5: Figure S5.** Differential mutation among the TME subtypes in TCGA cohorts. (A) Differential mutation of the LH and HL in TCGA LUAD (n = 501). (B) Differential mutation of the HH and HL in TCGA LUAD (n = 501). (C) Differential mutation of the LH and HL in TCGA SKCM (n = 352). (D) Differential mutation of the HH and HL in TCGA SKCM (n = 352). (E) Differential mutation of the LH and HL in TCGA HNSC (n = 514). (F) Differential mutation of the HH and HL in TCGA HNSC (n = 514).

**Additional file 6: Figure S6.** Mutation states of traditional driving genes in the TME subtypes from TCGA cohorts. (A) Driving gene mutation of the HH in TCGA LUAD (n = 501). (B) Driving gene mutation of the HL in TCGA LUAD (n = 501). (C) Driving gene mutation of the LH in TCGA LUAD (n = 501). (D) Driving gene mutation of the HH in TCGA SKCM (n = 352). (E) Driving gene mutation of the HL in TCGA SKCM (n = 352). (F) Driving gene mutation of the LH in TCGA SKCM (n = 352). (G) Driving gene mutation of the HH in TCGA HNSC (n = 514). (H)Driving gene mutation of the HL in TCGA HNSC (n = 514). (I) Driving gene mutation of the LH in TCGA HNSC (n = 514).

**Additional file 7: Figure S7.** The protein changes caused by mutations. (A) TP53 mutation sites between the HL and LH patients in TCGA LUAD (n = 501). (B) TP53 mutation sites between the HL and LH patients in HNSC (n = 514).

**Additional file 8: Figure S8.** Our stromal score showed the significant association with histologic GRADE. Data set is GSE9014, including 111 arrays of stroma (via Laser Capture Microdissected) from 53 breast cancer patients. (A) Boxplot of our stromal score in different histological GRADE. (B) Boxplot of our immune score in different histological GRADE. (C) Boxplot of ESTIMATE stromal score in different histological GRADE. (D) Boxplot of ESTIMATE immune score in different histological GRADE.

**Additional file 9: Table S1.** The basic information of included 15 datasets.

**Additional file 10: Table S2.** The details of the 5 immunotherapy Cohorts.

**Additional file 11: Table S3.** The top 20 enrichment terms of the exemplar genes in immune cluster by NMF.

**Additional file 12: Table S4.** The top 20 enrichment terms of the exemplar genes in stromal cluster by NMF.

**Additional file 13: Table S5.** The top 20 enrichment terms of the exemplar genes in other 9 cluster by NMF.

**Additional file 14: Table S6.** Immune and stromal gene signatures.

**Additional file 15: Table S7.** Gene overlap between our gene signatures and existing signatures.

**Additional file 16: Table S8.** Comparison with ESTIMATE and MCPcounter algorithms for prognosis and immunotherapy response prediction.

**Additional file 17: Table S9.** Table of acronyms.

**Additional file 18.** Additional Bioinformatics Analysis.

## Data Availability

The datasets generated and analysed during the current study are available in the NCBI GEO repository, accession numbers: GSE11969, GSE68465, GSE68571, GSE37749, GSE50081, GSE65858, GSE54467, GSE9014, GSE7339, GSE67501, GSE35640, GSE91061, GSE93157. The immunotherapy data 1 is available in Chen et al. [5]: Table S6a. https://doi.org/10.1158/2159-8290.CD-15-1545. The TCGA data are available in GDC TCGA, cancer codes: LUAD, SKCM, HNSC. Our ISTMEscore package is available in the GitHub repository, Zeng, Zihang. "ISTMEscore" Github Package: https://github.com/ZengZihang/ISTMEscore.
