# Supplementary figures and images for: Immune and stromal scoring system associated with tumor microenvironment and prognosis: a gene-based multi-cancer analysis
Source: J Transl Med. 2021 Aug 3;19:330. doi: 10.1186/s12967-021-03002-1 (PMC8336334; doi:10.1186/s12967-021-03002-1)

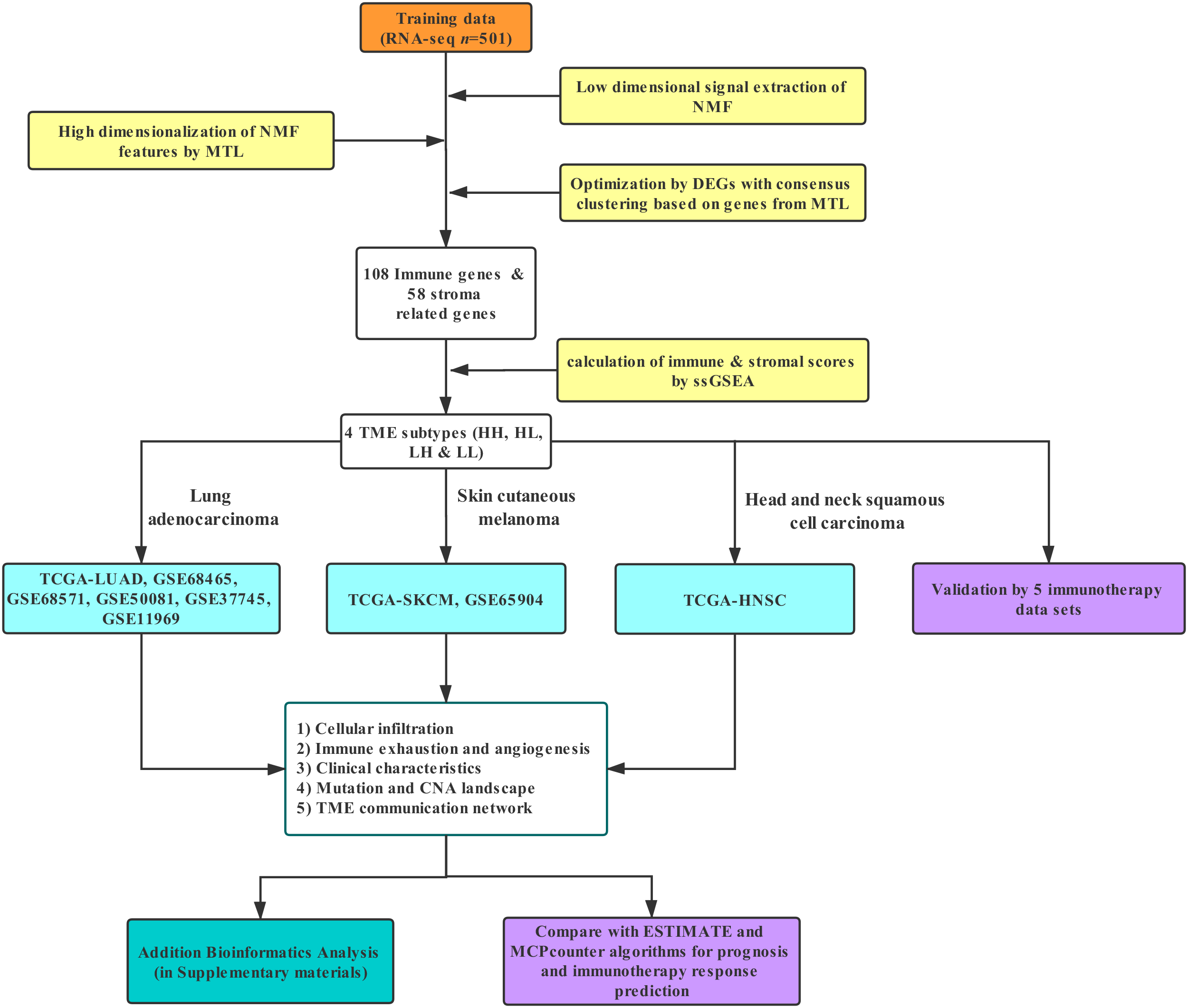

Supplement: Supplementary file 1 — Additional file 1: Figure S1. Illustration of identifying TME-related genes. [file 12967_2021_3002_MOESM1_ESM.tif]

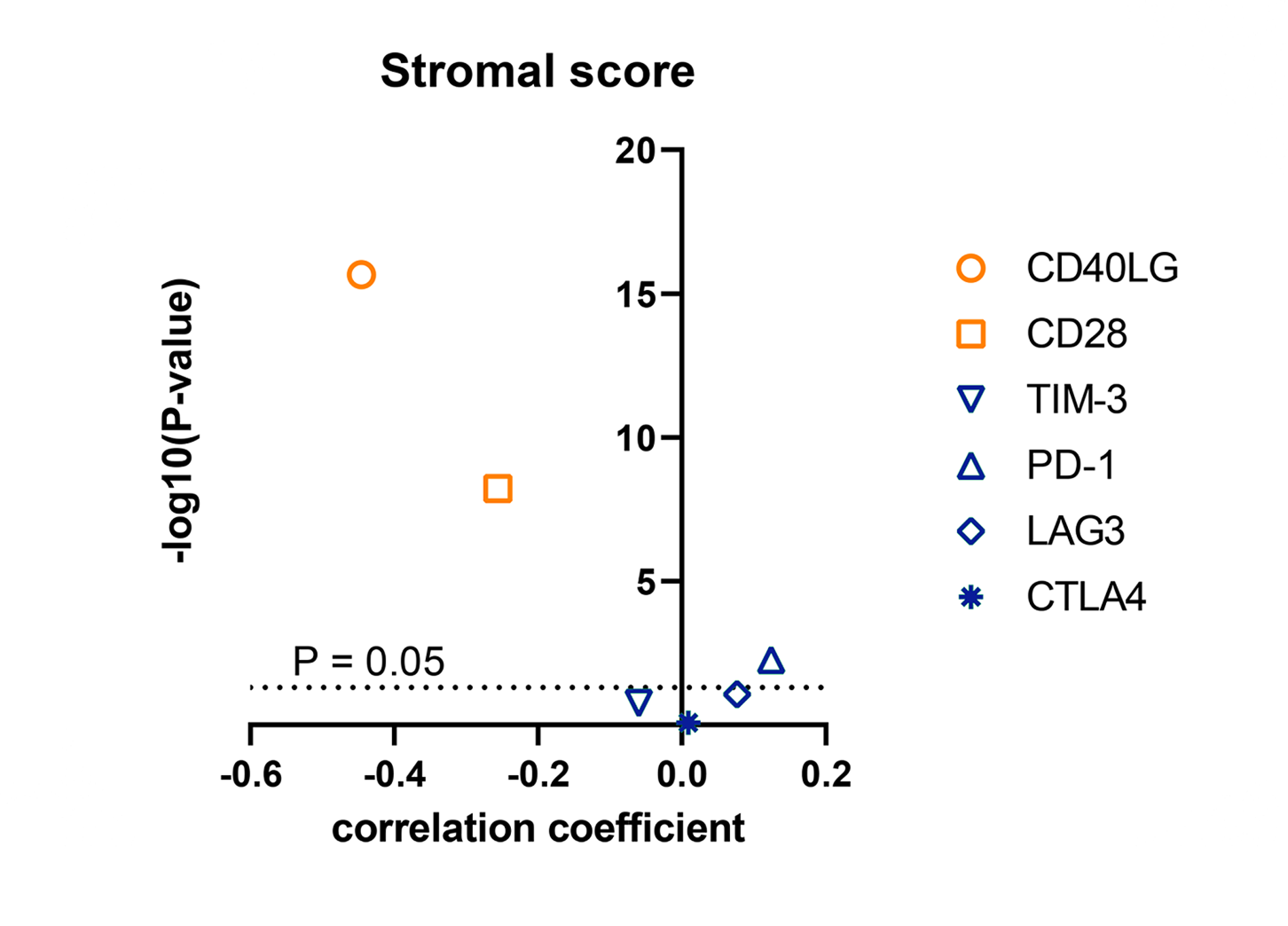

Supplement: Supplementary file 2 — Additional file 2: Figure S2. The correlation between stromal score and T cell co-stimulatory/suppression molecules. [file 12967_2021_3002_MOESM2_ESM.tif]

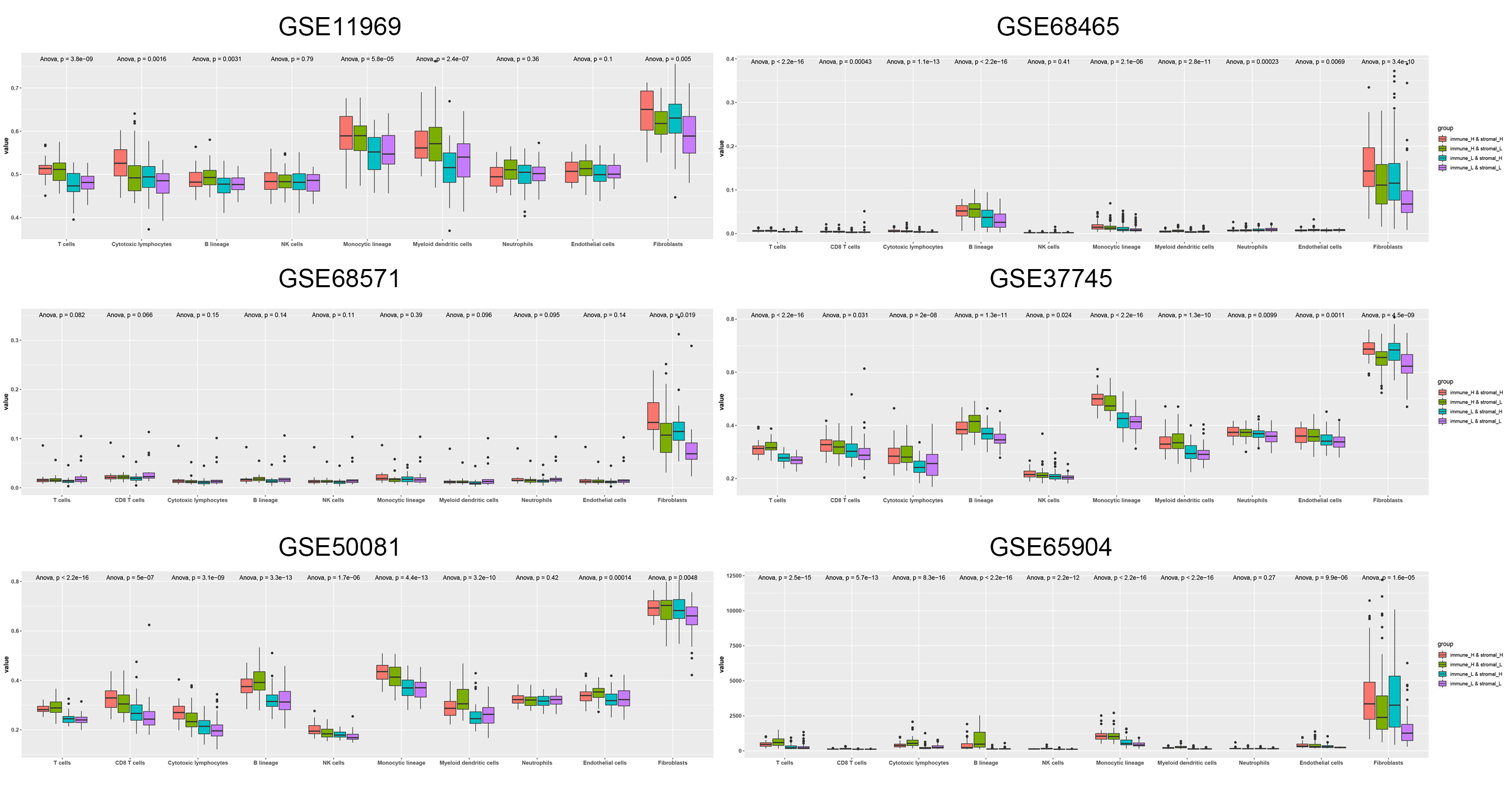

Supplement: Supplementary file 3 — Additional file 3: Figure S3. The cellular infiltration patterns in the 6 GEO validation cohorts. [file 12967_2021_3002_MOESM3_ESM.tif]

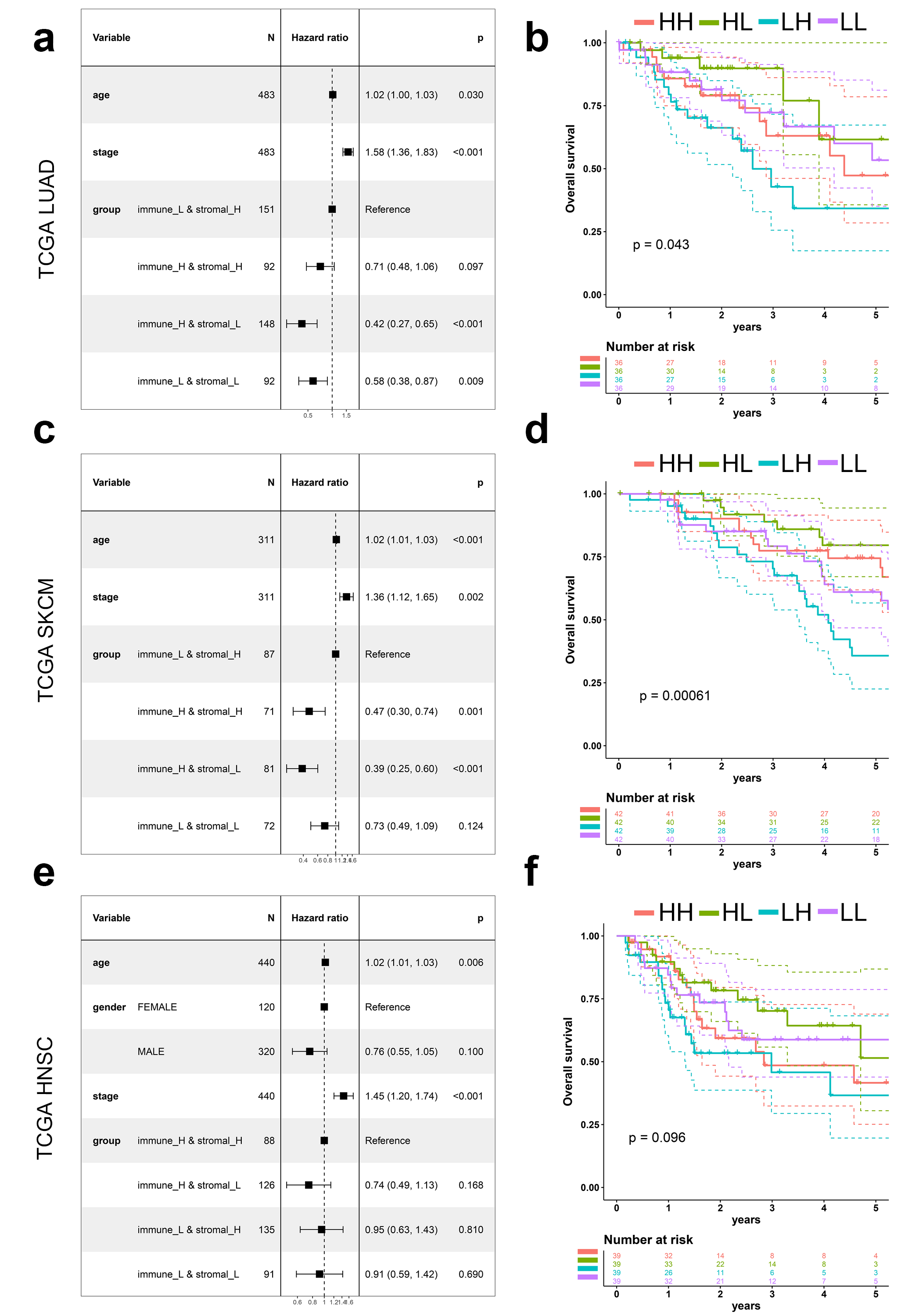

Supplement: Supplementary file 4 — Additional file 4: Figure S4. Multivariate Cox regression and propensity score matching analysis. (A) Multivariate Cox regression of clinical characteristics in TCGA LUAD (n = 501). (B) Survival curve after PSM in TCGA LUAD (n = 501). (C) Multivariate Cox regression of clinical characteristics in TCGA SKCM (n = 352). (D) Survival curve after PSM in TCGA SKCM (n = 352). (E) Multivariate Cox regression of clinical characteristics in TCGA HNSC (n = 514). (F) Survival curve after PSM in TCGA HNSC (n = 514). [file 12967_2021_3002_MOESM4_ESM.tif]

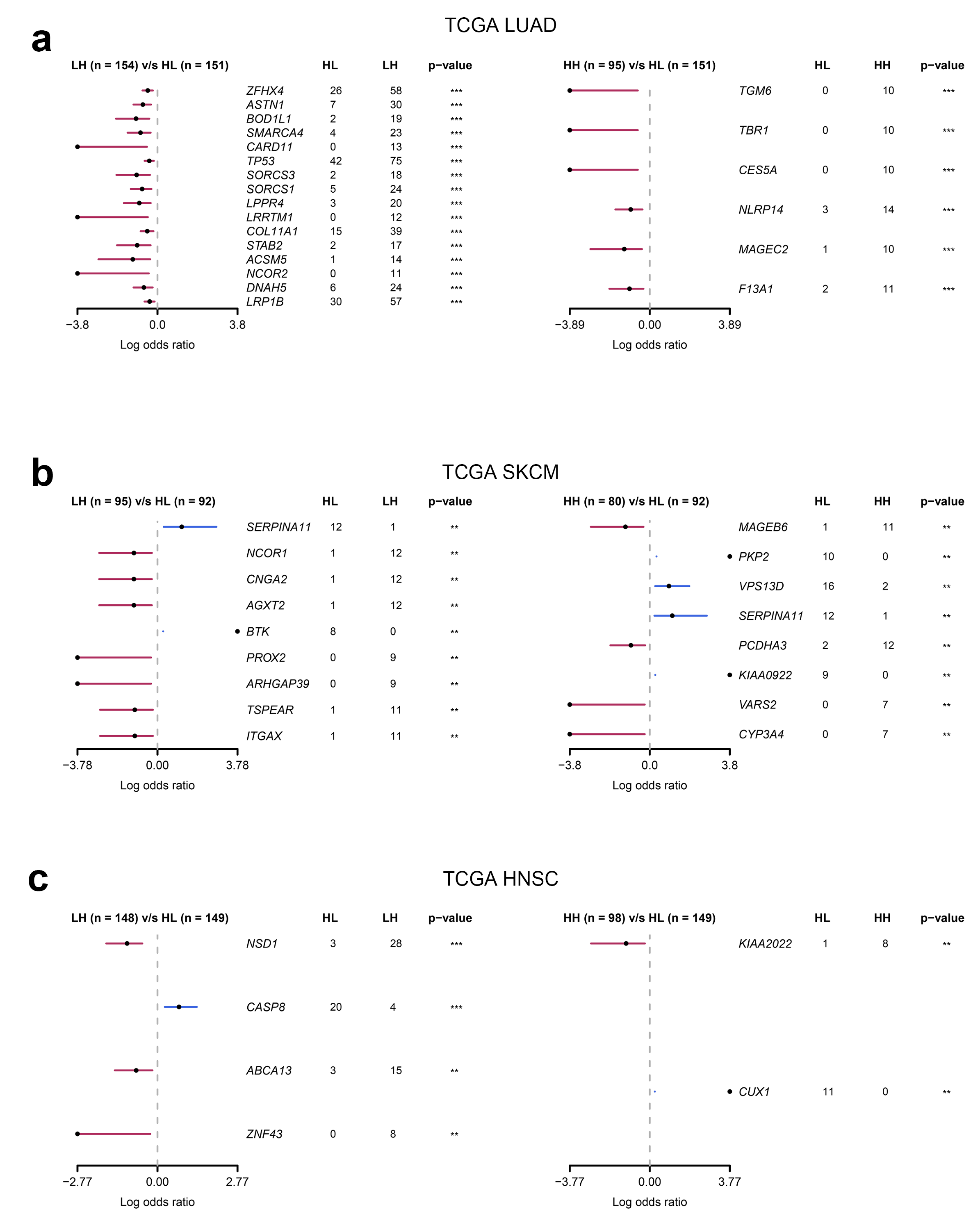

Supplement: Supplementary file 5 — Additional file 5: Figure S5. Differential mutation among the TME subtypes in TCGA cohorts. (A) Differential mutation of the LH and HL in TCGA LUAD (n = 501). (B) Differential mutation of the HH and HL in TCGA LUAD (n = 501). (C) Differential mutation of the LH and HL in TCGA SKCM (n = 352). (D) Differential mutation of the HH and HL in TCGA SKCM (n = 352). (E) Differential mutation of the LH and HL in TCGA HNSC (n = 514). (F) Differential mutation of the HH and HL in TCGA HNSC (n = 514). [file 12967_2021_3002_MOESM5_ESM.tif]

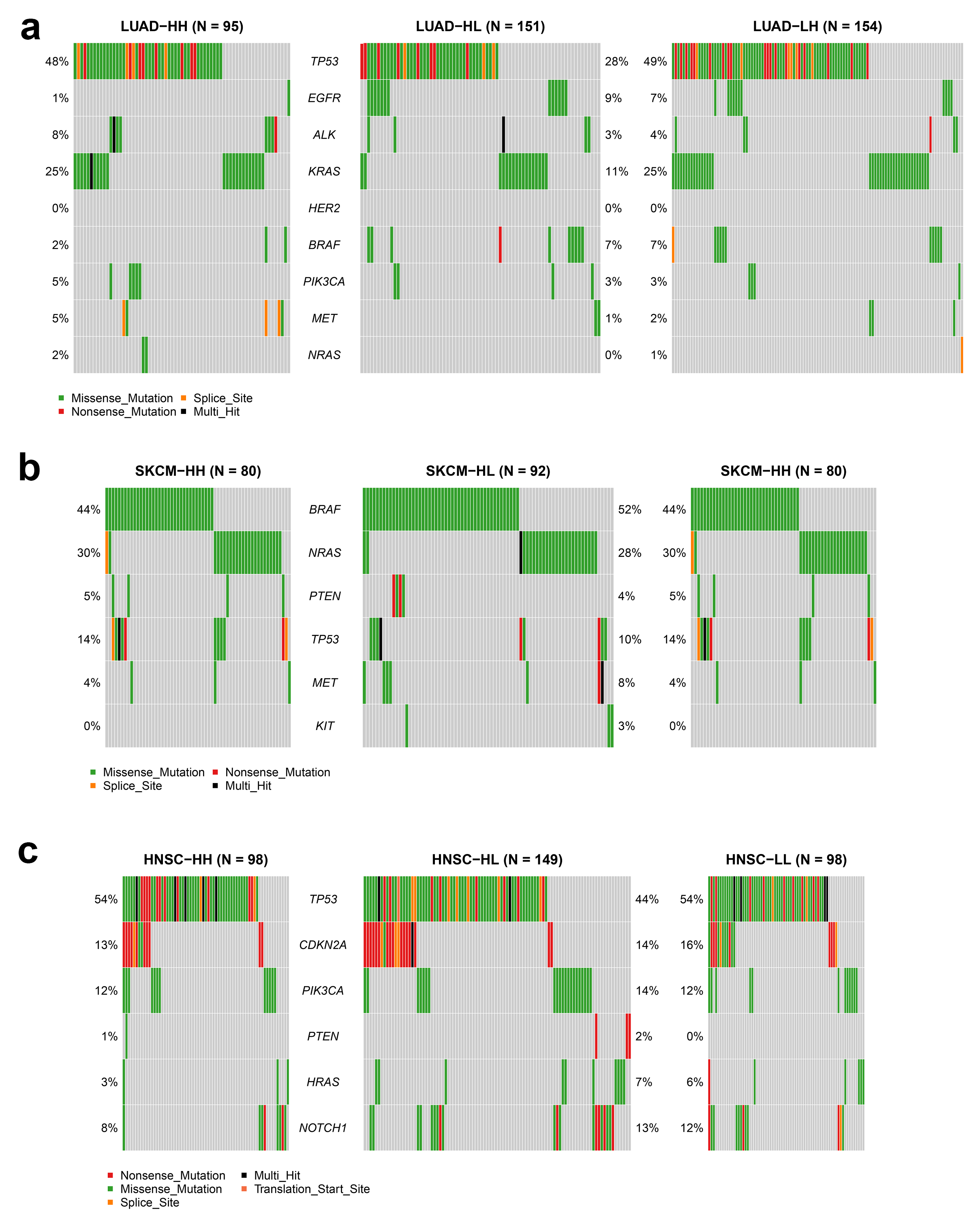

Supplement: Supplementary file 6 — Additional file 6: Figure S6. Mutation states of traditional driving genes in the TME subtypes from TCGA cohorts. (A) Driving gene mutation of the HH in TCGA LUAD (n = 501). (B) Driving gene mutation of the HL in TCGA LUAD (n = 501). (C) Driving gene mutation of the LH in TCGA LUAD (n = 501). (D) Driving gene mutation of the HH in TCGA SKCM (n = 352). (E) Driving gene mutation of the HL in TCGA SKCM (n = 352). (F) Driving gene mutation of the LH in TCGA SKCM (n = 352). (G) Driving gene mutation of the HH in TCGA HNSC (n = 514). (H)Driving gene mutation of the HL in TCGA HNSC (n = 514). (I) Driving gene mutation of the LH in TCGA HNSC (n = 514). [file 12967_2021_3002_MOESM6_ESM.tif]

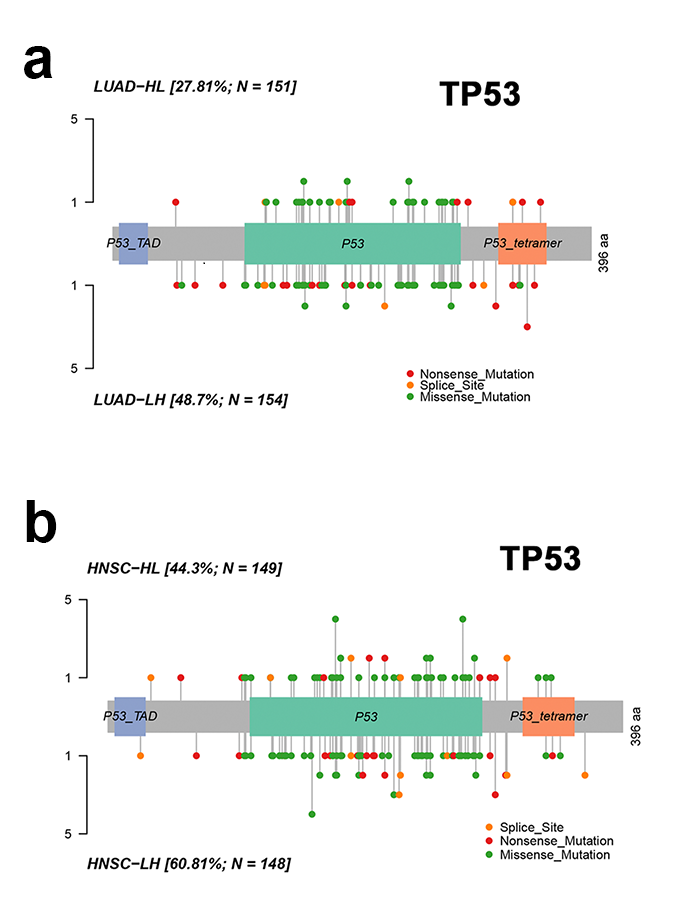

Supplement: Supplementary file 7 — Additional file 7: Figure S7. The protein changes caused by mutations. (A) TP53 mutation sites between the HL and LH patients in TCGA LUAD (n = 501). (B) TP53 mutation sites between the HL and LH patients in HNSC (n = 514). [file 12967_2021_3002_MOESM7_ESM.tif]

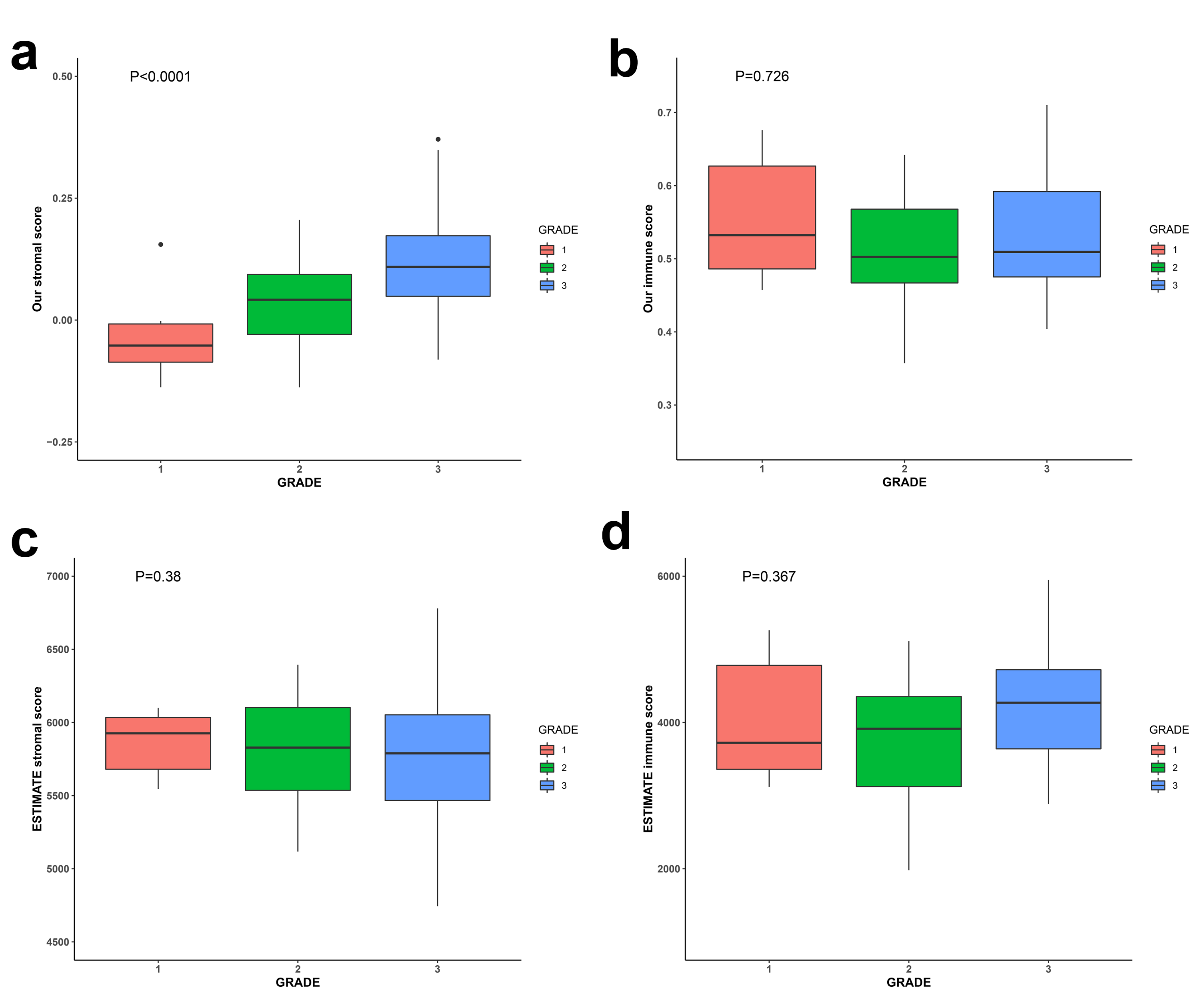

Supplement: Supplementary file 8 — Additional file 8: Figure S8. Our stromal score showed the significant association with histologic GRADE. Data set is GSE9014, including 111 arrays of stroma (via Laser Capture Microdissected) from 53 breast cancer patients. (A) Boxplot of our stromal score in different histological GRADE. (B) Boxplot of our immune score in different histological GRADE. (C) Boxplot of ESTIMATE stromal score in different histological GRADE. (D) Boxplot of ESTIMATE immune score in different histological GRADE. [file 12967_2021_3002_MOESM8_ESM.tif]
