# Supplementary material for: Immune and stromal scoring system associated with tumor microenvironment and prognosis: a gene-based multi-cancer analysis
Source: J Transl Med. 2021 Aug 3;19:330. doi: 10.1186/s12967-021-03002-1 (PMC8336334; doi:10.1186/s12967-021-03002-1)
Supplement: Supplementary file 9 — Additional file 9: Table S1. The basic information of included 15 datasets. [file 12967_2021_3002_MOESM9_ESM.pdf]

Datasets	N	Data Type	Histological Type	Reference
TCGA LUAD	501	Bulk RNA-seq, WES & clinical information	501 LUAD	Cancer Genome Atlas Research Network et al. Nat Genet 2013;45(10):1113-1120. PMID: 24071849
TCGA SKCM	352	Bulk RNA-seq, WES & clinical information	352 SKCM	Cancer Genome Atlas Research Network et al. Nat Genet 2013;45(10):1113-1120. PMID: 24071849
TCGA HNSC	514	Bulk RNA-seq, WES & clinical information	514 HNSC	Cancer Genome Atlas Research Network et al. Nat Genet 2013;45(10):1113-1120. PMID: 24071849
GSE11969	149	Bulk RNA-chip & clinical information	94 LUAD & 55 other NSCLC	Takeuchi T et al. J Clin Oncol 2006 Apr 10;24(11):1679-88. PMID: 16549822
GSE68465	442	Bulk RNA-chip & clinical information	442 LUAD	Shedden K et al. Nat Med 2008 Aug;14(8):822-7. PMID: 18641660
GSE68571	86	Bulk RNA-chip & clinical information	86 LUAD	Beer DG et al. Nat Med 2002 Aug;8(8):816-24. PMID: 12118244
GSE37745	196	Bulk RNA-chip & clinical information	106 LUAD & 90 other NSCLC	Botling J et al. Clin Cancer Res 2013 Jan 1;19(1):194-204. PMID: 23032747
GSE50081	172	Bulk RNA-chip & clinical information	127 LUAD & 45 other NSCLC	Der SD et al. J Thorac Oncol 2014 Jan;9(1):59-64. PMID: 24305008
GSE65904	214	Bulk RNA-chip & clinical information	214 melanoma	Jayawardana K et al. Int J Cancer 2015 Feb 15;136(4):863-74. PMID: 24975271
GSE9014	111	Bulk RNA-chip in tumor-associated stroma	111 breast cancer	Finak G et al. Nat Med 2008 May;14(5):518-27. PMID: 18438415
Immunotherapy Data Set 1 (Chen etal)	31	Immune Profiling by IHC & clinical information	31 melanoma	Chen P L et al. Cancer Discovery, 2016, 6(8):827-837. PMID: 27301722
Immunotherapy Data Set 2 (GSE91061)	65	Bulk RNA-seq & clinical information	65 melanoma	Riaz N et al. Cell 2017 Nov 2;171(4):934-949.e16. PMID: 29033130
Immunotherapy Data Set 3 (GSE93157)	65	NanoString nCounter & clinical information	35 NSCLC, 5 HNSC & 25 melanoma	Prat A et a. Cancer Res 2017 Jul 1;77(13):3540-3550. PMID: 28487385

Immunotherapy Data Set 4 (GSE67501)	11	NanoString nCounter & clinical information	11 renal cell carcinoma	Ascierto ML et al. Cancer Immunol Res 2016 Sep 2;4(9):726-33. PMID: 27491898
Immunotherapy Data Set 5 (GSE35640)	56	Bulk RNA-chip & clinical information	56 melanoma	Ulloa-Montoya F et al. J Clin Oncol 2013 Jul 1;31(19):2388-95. PMID: 23715562

Supplementary table1 The basic information of included 15 datasets. LUAD, lung adenocarcinoma; SKCM, skin cutaneous melanoma; HNSC, head and neck squamous cell carcinomal; NSCLC, non-small cell lung cancer.
