# Supplementary material for: Immune and stromal scoring system associated with tumor microenvironment and prognosis: a gene-based multi-cancer analysis
Source: J Transl Med. 2021 Aug 3;19:330. doi: 10.1186/s12967-021-03002-1 (PMC8336334; doi:10.1186/s12967-021-03002-1)
Supplement: Supplementary file 10 — Additional file 10: Table S2. The details of the 5 immunotherapy Cohorts. [file 12967_2021_3002_MOESM10_ESM.pdf]

Datasets	Sample details	Therapy	Biopsy time point	Clinical response
Immunotherapy Data Set 1 (Chen etal)	16 pre-anti-CTLA4 therapy cases, 5 on-anti-CTLA4 therapy cases, 15 pre-anti-PD-1 therapy cases and 10 on-anti-PD-1 therapy cases	anti-CTLA4 & anti-PD-1	4.4 months before therapy, 3.2 months on anti-CTLA4 therapy, 3 months before anti-PD-1 therapy, 1.4 months on anti-PD-1 therapy, and 4.4 months after restaging	≧ SD by RECIST (Response Evaluation Criteria in Solid Tumors)
Immunotherapy Data Set 2 (GSE91061)	51 pre-treatment cases and 57 on-treatment cases	anti-PD-1	1-7 days before therapy and 23-29 days on cycle 1 therapy	≧ SD by RECIST v1.1
Immunotherapy Data Set 3 (GSE93157)	65 pre-treatment cases	anti-PD-1	none	≧ SD by modified RECIST 1.1
Immunotherapy Data Set 4 (GSE67501)	11 pre-treatment cases	anti-PD-1	2-81 months before therapy	≧ SD by RECIST
Immunotherapy Data Set 5 (GSE35640)	56 pre-treatment cases	anti-MAGE-A3	none	≧ SD by RECIST v1.0

Supplementary table2 The details of the 5 immunotherapy Cohorts.
