# Supplementary material for: Immune and stromal scoring system associated with tumor microenvironment and prognosis: a gene-based multi-cancer analysis
Source: J Transl Med. 2021 Aug 3;19:330. doi: 10.1186/s12967-021-03002-1 (PMC8336334; doi:10.1186/s12967-021-03002-1)
Supplement: Supplementary file 11 — Additional file 11: Table S3. The top 20 enrichment terms of the exemplar genes in immune cluster by NMF. [file 12967_2021_3002_MOESM11_ESM.pdf]

ID	Description	GeneRatio	BgRatio	pvalue	p.adjust	qvalue
GO:0006959	humoral immune response	19/92	349/18493	6.99E-15	1.38E-11	1.05E-11
GO:0002697	regulation of immune effector process	19/92	439/18493	4.30E-13	4.24E-10	3.23E-10
GO:0042110	T cell activation	19/92	451/18493	6.93E-13	4.55E-10	3.47E-10
GO:0046651	lymphocyte proliferation	15/92	264/18493	3.44E-12	1.51E-09	1.15E-09
GO:0032943	mononuclear cell proliferation	15/92	266/18493	3.83E-12	1.51E-09	1.15E-09
GO:0070661	leukocyte proliferation	15/92	283/18493	9.31E-12	3.06E-09	2.33E-09
GO:0034341	response to interferon-gamma	13/92	198/18493	1.74E-11	4.82E-09	3.67E-09
GO:0002460	adaptive immune response based on somatic recombination of immune receptors built from immunoglobulin superfamily domains	16/92	354/18493	1.95E-11	4.82E-09	3.67E-09
GO:0050863	regulation of T cell activation	15/92	305/18493	2.70E-11	5.70E-09	4.34E-09
GO:0002478	antigen processing and presentation of exogenous peptide antigen	11/92	126/18493	3.18E-11	5.70E-09	4.34E-09
GO:0002920	regulation of humoral immune response	11/92	126/18493	3.18E-11	5.70E-09	4.34E-09
GO:0019886	antigen processing and presentation of exogenous peptide antigen via MHC class II	10/92	98/18493	5.50E-11	9.02E-09	6.87E-09
GO:0019884	antigen processing and presentation of exogenous antigen	11/92	134/18493	6.23E-11	9.02E-09	6.87E-09
GO:0019882	antigen processing and presentation	12/92	175/18493	6.64E-11	9.02E-09	6.87E-09
GO:0002495	antigen processing and presentation of peptide antigen via MHC class II	10/92	101/18493	7.46E-11	9.02E-09	6.87E-09
GO:0042098	T cell proliferation	12/92	178/18493	8.10E-11	9.02E-09	6.87E-09
GO:0071346	cellular response to interferon-gamma	12/92	178/18493	8.10E-11	9.02E-09	6.87E-09
GO:0002504	antigen processing and presentation of peptide or polysaccharide antigen via MHC class II	10/92	102/18493	8.23E-11	9.02E-09	6.87E-09
GO:0048002	antigen processing and presentation of peptide antigen	11/92	139/18493	9.28E-11	9.63E-09	7.33E-09
GO:0042129	regulation of T cell proliferation	11/92	153/18493	2.62E-10	2.58E-08	1.96E-08

Supplementary table3 The top 20 enrichment terms of the exemplar genes in immune cluster by NMF.
