# Supplementary material for: Immune and stromal scoring system associated with tumor microenvironment and prognosis: a gene-based multi-cancer analysis
Source: J Transl Med. 2021 Aug 3;19:330. doi: 10.1186/s12967-021-03002-1 (PMC8336334; doi:10.1186/s12967-021-03002-1)
Supplement: Supplementary file 12 — Additional file 12: Table S4. The top 20 enrichment terms of the exemplar genes in stromal cluster by NMF. [file 12967_2021_3002_MOESM12_ESM.pdf]

ID	Description	GeneRatio	BgRatio	pvalue	p.adjust	qvalue
GO:0030198	extracellular matrix organization	51/90	348/18493	5.76E-65	9.84E-62	7.30E-62
GO:0043062	extracellular structure organization	51/90	402/18493	1.38E-61	1.18E-58	8.77E-59
GO:0030199	collagen fibril organization	19/90	52/18493	1.01E-31	5.74E-29	4.26E-29
GO:0061448	connective tissue development	20/90	263/18493	1.16E-18	4.96E-16	3.68E-16
GO:0051216	cartilage development	17/90	206/18493	1.47E-16	5.02E-14	3.73E-14
GO:0048705	skeletal system morphogenesis	17/90	236/18493	1.45E-15	4.12E-13	3.06E-13
GO:0031589	cell-substrate adhesion	19/90	338/18493	2.53E-15	6.17E-13	4.58E-13
GO:0035987	endodermal cell differentiation	10/90	45/18493	1.24E-14	2.64E-12	1.96E-12
GO:0002062	chondrocyte differentiation	13/90	117/18493	1.44E-14	2.72E-12	2.02E-12
GO:0001706	endoderm formation	10/90	50/18493	3.90E-14	6.66E-12	4.94E-12
GO:0001503	ossification	18/90	380/18493	2.81E-13	4.36E-11	3.24E-11
GO:0002063	chondrocyte development	9/90	45/18493	7.80E-13	1.11E-10	8.23E-11
GO:0060348	bone development	14/90	208/18493	1.42E-12	1.87E-10	1.39E-10
GO:0007492	endoderm development	10/90	74/18493	2.48E-12	3.03E-10	2.25E-10
GO:0007178	transmembrane receptor protein serine/threonine kinase signaling pathway	16/90	340/18493	7.51E-12	8.55E-10	6.34E-10
GO:0001704	formation of primary germ layer	11/90	118/18493	1.21E-11	1.29E-09	9.57E-10
GO:0071559	response to transforming growth factor beta	14/90	249/18493	1.63E-11	1.63E-09	1.21E-09
GO:0071560	cellular response to transforming growth factor beta stimulus	13/90	242/18493	1.61E-10	1.53E-08	1.13E-08
GO:0010810	regulation of cell-substrate adhesion	12/90	209/18493	4.00E-10	3.60E-08	2.67E-08
GO:0007160	cell-matrix adhesion	12/90	222/18493	8.00E-10	6.83E-08	5.06E-08

Supplementary table4 The top 20 enrichment terms of the exemplar genes in stromal cluster by NMF.
