# Supplementary material for: Immune and stromal scoring system associated with tumor microenvironment and prognosis: a gene-based multi-cancer analysis
Source: J Transl Med. 2021 Aug 3;19:330. doi: 10.1186/s12967-021-03002-1 (PMC8336334; doi:10.1186/s12967-021-03002-1)
Supplement: Supplementary file 13 — Additional file 13: Table S5. The top 20 enrichment terms of the exemplar genes in other 9 cluster by NMF. [file 12967_2021_3002_MOESM13_ESM.pdf]

Cluster	ID	Description	GeneRatio	BgRatio	pvalue	p.adjust	qvalue
1	GO:0030195	negative regulation of blood coagulation	5/87	52/18493	4.48E-06	0.002939288	0.002604505
1	GO:1900047	negative regulation of hemostasis	5/87	52/18493	4.48E-06	0.002939288	0.002604505
1	GO:0050819	negative regulation of coagulation	5/87	56/18493	6.49E-06	0.002939288	0.002604505
1	GO:0061045	negative regulation of wound healing	5/87	72/18493	2.24E-05	0.004944213	0.004381069
1	GO:0030193	regulation of blood coagulation	5/87	77/18493	3.11E-05	0.004944213	0.004381069
1	GO:1900046	regulation of hemostasis	5/87	77/18493	3.11E-05	0.004944213	0.004381069
1	GO:0003002	regionalization	9/87	341/18493	3.21E-05	0.004944213	0.004381069
1	GO:0061041	regulation of wound healing	6/87	134/18493	4.04E-05	0.004944213	0.004381069
1	GO:0007586	digestion	6/87	135/18493	4.21E-05	0.004944213	0.004381069
1	GO:0050818	regulation of coagulation	5/87	82/18493	4.21E-05	0.004944213	0.004381069
1	GO:0034116	positive regulation of heterotypic cell-cell adhesion	3/87	15/18493	4.39E-05	0.004944213	0.004381069
1	GO:0010038	response to metal ion	9/87	358/18493	4.69E-05	0.004944213	0.004381069
1	GO:1903035	negative regulation of response to wounding	5/87	84/18493	4.73E-05	0.004944213	0.004381069
1	GO:0009952	anterior/posterior pattern specification	7/87	211/18493	6.15E-05	0.005969257	0.005289362
1	GO:0022600	digestive system process	5/87	97/18493	9.41E-05	0.00852684	0.007555637

1	GO:1903034	regulation of response to wounding	6/87	160/18493	0.000108178	0.008649663	0.007664471
1	GO:0030277	maintenance of gastrointestinal epithelium	3/87	20/18493	0.0001082	0.008649663	0.007664471
1	GO:0031639	plasminogen activation	3/87	24/18493	0.000189507	0.014307794	0.012678143
1	GO:0007389	pattern specification process	9/87	434/18493	0.000202159	0.014459689	0.012812737
1	GO:0034114	regulation of heterotypic cell-cell adhesion	3/87	25/18493	0.000214618	0.014583309	0.012922277
2	GO:0030168	platelet activation	9/85	152/18493	3.26E-08	7.04E-05	5.65E-05
2	GO:0034330	cell junction organization	10/85	287/18493	7.78E-07	0.000790271	0.000634407
2	GO:0034109	homotypic cell-cell adhesion	6/85	76/18493	1.33E-06	0.000790271	0.000634407
2	GO:0034329	cell junction assembly	9/85	238/18493	1.47E-06	0.000790271	0.000634407
2	GO:0050878	regulation of body fluid levels	12/85	493/18493	2.54E-06	0.001094302	0.000878475
2	GO:0007044	cell-substrate junction assembly	6/85	95/18493	4.94E-06	0.001776666	0.001426258
2	GO:0070527	platelet aggregation	5/85	57/18493	6.32E-06	0.001947512	0.001563408
2	GO:0045070	positive regulation of viral genome replication	4/85	36/18493	2.19E-05	0.005106185	0.004099102
2	GO:0007596	blood coagulation	9/85	334/18493	2.26E-05	0.005106185	0.004099102
2	GO:0007599	hemostasis	9/85	339/18493	2.54E-05	0.005106185	0.004099102
2	GO:0050817	coagulation	9/85	340/18493	2.60E-05	0.005106185	0.004099102

2	GO:0050772	positive regulation of axonogenesis	5/85	81/18493	3.55E-05	0.006376471	0.005118852
2	GO:0051653	spindle localization	4/85	42/18493	4.07E-05	0.006756534	0.005423955
2	GO:0048524	positive regulation of viral process	5/85	86/18493	4.74E-05	0.007296721	0.005857602
2	GO:0010770	positive regulation of cell morphogenesis involved in differentiation	6/85	147/18493	5.94E-05	0.008545374	0.006859986
2	GO:0001764	neuron migration	6/85	149/18493	6.41E-05	0.008636824	0.006933399
2	GO:0061564	axon development	10/85	493/18493	8.65E-05	0.01097253	0.008808438
2	GO:0007229	integrin-mediated signaling pathway	5/85	99/18493	9.28E-05	0.010974865	0.008810313
2	GO:0007517	muscle organ development	9/85	403/18493	9.68E-05	0.010974865	0.008810313
2	GO:0043902	positive regulation of multi-organism process	6/85	162/18493	0.00010176	0.010974865	0.008810313
3	GO:0006614	SRP-dependent cotranslational protein targeting to membrane	50/95	105/18493	1.01E-91	1.82E-88	1.55E-88
3	GO:0006613	cotranslational protein targeting to membrane	50/95	109/18493	1.22E-90	1.10E-87	9.38E-88
3	GO:0000184	nuclear-transcribed mRNA catabolic process, nonsense-mediated decay	51/95	120/18493	2.15E-90	1.29E-87	1.10E-87
3	GO:0045047	protein targeting to ER	50/95	117/18493	1.26E-88	5.66E-86	4.81E-86
3	GO:0072599	establishment of protein localization to endoplasmic reticulum	50/95	121/18493	1.09E-87	3.92E-85	3.33E-85
3	GO:0070972	protein localization to endoplasmic reticulum	51/95	146/18493	5.90E-85	1.77E-82	1.51E-82
3	GO:0006413	translational initiation	54/95	194/18493	4.01E-84	1.03E-81	8.77E-82

3	GO:0000956	nuclear-transcribed mRNA catabolic process	51/95	207/18493	6.96E-76	1.57E-73	1.33E-73
3	GO:0006612	protein targeting to membrane	50/95	198/18493	7.35E-75	1.47E-72	1.25E-72
3	GO:0006402	mRNA catabolic process	55/95	307/18493	4.66E-74	8.40E-72	7.14E-72
3	GO:0006401	RNA catabolic process	55/95	340/18493	2.02E-71	3.32E-69	2.82E-69
3	GO:0090150	establishment of protein localization to membrane	53/95	317/18493	2.59E-69	3.88E-67	3.30E-67
3	GO:0006605	protein targeting	54/95	424/18493	5.41E-64	7.50E-62	6.38E-62
3	GO:0042254	ribosome biogenesis	26/95	282/18493	1.07E-25	1.37E-23	1.17E-23
3	GO:0022613	ribonucleoprotein complex biogenesis	30/95	485/18493	1.47E-24	1.76E-22	1.50E-22
3	GO:0002181	cytoplasmic translation	18/95	93/18493	6.07E-24	6.84E-22	5.81E-22
3	GO:0006364	rRNA processing	18/95	205/18493	1.58E-17	1.68E-15	1.42E-15
3	GO:0016072	rRNA metabolic process	18/95	240/18493	2.63E-16	2.64E-14	2.24E-14
3	GO:0042274	ribosomal small subunit biogenesis	12/95	63/18493	3.55E-16	3.36E-14	2.86E-14
3	GO:0042273	ribosomal large subunit biogenesis	12/95	73/18493	2.34E-15	2.11E-13	1.80E-13
4	GO:0035082	axoneme assembly	8/42	61/18493	9.41E-13	2.23E-10	1.95E-10
4	GO:0003341	cilium movement	8/42	63/18493	1.23E-12	2.23E-10	1.95E-10
4	GO:0007018	microtubule-based movement	11/42	269/18493	1.44E-11	1.74E-09	1.53E-09

4	GO:0001578	microtubule bundle formation	8/42	89/18493	2.15E-11	1.95E-09	1.71E-09
4	GO:0070286	axonemal dynein complex assembly	6/42	31/18493	6.67E-11	4.83E-09	4.23E-09
4	GO:0036158	outer dynein arm assembly	4/42	17/18493	5.35E-08	3.23E-06	2.83E-06
4	GO:0060271	cilium assembly	9/42	366/18493	1.06E-07	5.47E-06	4.79E-06
4	GO:0044782	cilium organization	9/42	382/18493	1.52E-07	6.88E-06	6.03E-06
4	GO:0001539	cilium or flagellum-dependent cell motility	4/42	34/18493	1.01E-06	3.67E-05	3.21E-05
4	GO:0060285	cilium-dependent cell motility	4/42	34/18493	1.01E-06	3.67E-05	3.21E-05
4	GO:0030317	flagellated sperm motility	5/42	89/18493	1.70E-06	5.14E-05	4.50E-05
4	GO:0097722	sperm motility	5/42	89/18493	1.70E-06	5.14E-05	4.50E-05
4	GO:0007286	spermatid development	4/42	146/18493	0.000330503	0.009203251	0.008055186
4	GO:0048515	spermatid differentiation	4/42	152/18493	0.000385108	0.009957803	0.008715611
4	GO:0036159	inner dynein arm assembly	2/42	14/18493	0.000450368	0.01086889	0.009513044
4	GO:0003352	regulation of cilium movement	2/42	15/18493	0.000518908	0.011740286	0.010275737
4	GO:0060294	cilium movement involved in cell motility	2/42	19/18493	0.000840224	0.017891823	0.015659897
4	GO:0060632	regulation of microtubule-based movement	2/42	26/18493	0.00158091	0.03179386	0.027827717
4	GO:0007368	determination of left/right symmetry	3/42	116/18493	0.002310129	0.044014039	0.038523483

4	GO:0007281	germ cell development	4/42	255/18493	0.002619209	0.046984838	0.041123688
7	GO:0008544	epidermis development	16/93	458/18493	1.02E-09	1.54E-06	1.41E-06
7	GO:0031581	hemidesmosome assembly	5/93	12/18493	2.22E-09	1.67E-06	1.53E-06
7	GO:0070268	cornification	8/93	112/18493	9.34E-08	4.69E-05	4.30E-05
7	GO:0043588	skin development	13/93	412/18493	1.42E-07	5.37E-05	4.92E-05
7	GO:0030277	maintenance of gastrointestinal epithelium	4/93	20/18493	2.73E-06	0.000822841	0.000754072
7	GO:0010669	epithelial structure maintenance	4/93	27/18493	9.63E-06	0.002417768	0.002215703
7	GO:0016266	O-glycan processing	5/93	61/18493	1.37E-05	0.002958951	0.002711657
7	GO:0031424	keratinization	8/93	224/18493	1.72E-05	0.003240814	0.002969963
7	GO:0042303	molting cycle	6/93	113/18493	2.25E-05	0.003395884	0.003112074
7	GO:0042633	hair cycle	6/93	113/18493	2.25E-05	0.003395884	0.003112074
7	GO:0007586	digestion	6/93	135/18493	6.13E-05	0.007921076	0.007259072
7	GO:0030198	extracellular matrix organization	9/93	348/18493	6.40E-05	0.007921076	0.007259072
7	GO:0009913	epidermal cell differentiation	9/93	351/18493	6.83E-05	0.007921076	0.007259072
7	GO:0007044	cell-substrate junction assembly	5/93	95/18493	0.000117023	0.012410186	0.011373006
7	GO:0022600	digestive system process	5/93	97/18493	0.000129138	0.012410186	0.011373006

7	GO:0030216	keratinocyte differentiation	8/93	299/18493	0.00013176	0.012410186	0.011373006
7	GO:0006493	protein O-linked glycosylation	5/93	102/18493	0.00016364	0.014506164	0.013293813
7	GO:0043062	extracellular structure organization	9/93	402/18493	0.000190463	0.01572122	0.014407321
7	GO:0034329	cell junction assembly	7/93	238/18493	0.00019821	0.01572122	0.014407321
7	GO:0060706	cell differentiation involved in embryonic placenta development	3/93	26/18493	0.000294363	0.021874578	0.020046413
8	GO:0043129	surfactant homeostasis	5/90	11/18493	1.10E-09	2.58E-06	2.15E-06
8	GO:0048875	chemical homeostasis within a tissue	5/90	13/18493	3.04E-09	3.56E-06	2.98E-06
8	GO:0001894	tissue homeostasis	10/90	221/18493	1.20E-07	9.14E-05	7.64E-05
8	GO:0030260	entry into host cell	8/90	133/18493	2.74E-07	9.14E-05	7.64E-05
8	GO:0044409	entry into host	8/90	133/18493	2.74E-07	9.14E-05	7.64E-05
8	GO:0051806	entry into cell of other organism involved in symbiotic interaction	8/90	133/18493	2.74E-07	9.14E-05	7.64E-05
8	GO:0051828	entry into other organism involved in symbiotic interaction	8/90	133/18493	2.74E-07	9.14E-05	7.64E-05
8	GO:0019058	viral life cycle	10/90	289/18493	1.41E-06	0.000413326	0.000345422
8	GO:0048871	multicellular organismal homeostasis	12/90	471/18493	2.93E-06	0.000762394	0.000637144
8	GO:0060249	anatomical structure homeostasis	11/90	425/18493	6.76E-06	0.001582037	0.001322132
8	GO:0051701	interaction with host	8/90	209/18493	8.16E-06	0.001734354	0.001449425

8	GO:1903902	positive regulation of viral life cycle	5/90	60/18493	1.08E-05	0.002103603	0.001758012
8	GO:0050900	leukocyte migration	11/90	478/18493	2.03E-05	0.003647077	0.003047916
8	GO:0001666	response to hypoxia	9/90	314/18493	2.21E-05	0.003696905	0.003089559
8	GO:0043062	extracellular structure organization	10/90	402/18493	2.56E-05	0.00398946	0.00333405
8	GO:0046718	viral entry into host cell	6/90	121/18493	2.75E-05	0.00398946	0.00333405
8	GO:0036293	response to decreased oxygen levels	9/90	325/18493	2.90E-05	0.00398946	0.00333405
8	GO:0070482	response to oxygen levels	9/90	349/18493	5.05E-05	0.006556823	0.005479634
8	GO:0045056	transcytosis	3/90	16/18493	5.96E-05	0.007287217	0.006090035
8	GO:0048524	positive regulation of viral process	5/90	86/18493	6.23E-05	0.007287217	0.006090035
9	GO:0016999	antibiotic metabolic process	5/77	152/18493	0.000431063	0.268632674	0.252423054
9	GO:0015850	organic hydroxy compound transport	6/77	255/18493	0.000678157	0.268632674	0.252423054
9	GO:0043129	surfactant homeostasis	2/77	11/18493	0.000918555	0.268632674	0.252423054
9	GO:0045104	intermediate filament cytoskeleton organization	3/77	49/18493	0.00111419	0.268632674	0.252423054
9	GO:0045103	intermediate filament-based process	3/77	50/18493	0.001181777	0.268632674	0.252423054
9	GO:0048875	chemical homeostasis within a tissue	2/77	13/18493	0.001295662	0.268632674	0.252423054
9	GO:0006805	xenobiotic metabolic process	4/77	117/18493	0.001443057	0.268632674	0.252423054

9	GO:0001755	neural crest cell migration	3/77	55/18493	0.00155842	0.268632674	0.252423054
9	GO:0042448	progesterone metabolic process	2/77	16/18493	0.001977258	0.301815856	0.283603923
9	GO:1902644	tertiary alcohol metabolic process	2/77	17/18493	0.002234859	0.301815856	0.283603923
9	GO:0007586	digestion	4/77	135/18493	0.002434744	0.301815856	0.283603923
9	GO:0018904	ether metabolic process	2/77	19/18493	0.0027949	0.301815856	0.283603923
9	GO:0016137	glycoside metabolic process	2/77	20/18493	0.003097091	0.301815856	0.283603923
9	GO:0006898	receptor-mediated endocytosis	6/77	346/18493	0.003193019	0.301815856	0.283603923
9	GO:0030198	extracellular matrix organization	6/77	348/18493	0.003284708	0.301815856	0.283603923
9	GO:0006081	cellular aldehyde metabolic process	3/77	73/18493	0.003501852	0.301815856	0.283603923
9	GO:0034308	primary alcohol metabolic process	3/77	76/18493	0.003923011	0.318225446	0.299023338
9	GO:0014032	neural crest cell development	3/77	78/18493	0.004220193	0.318794386	0.299557947
9	GO:0015721	bile acid and bile salt transport	2/77	25/18493	0.004824778	0.318794386	0.299557947
9	GO:0014031	mesenchymal cell development	3/77	82/18493	0.004854737	0.318794386	0.299557947
10	GO:0006402	mRNA catabolic process	14/95	307/18493	5.49E-10	1.05E-06	7.93E-07
10	GO:0006401	RNA catabolic process	14/95	340/18493	2.06E-09	1.97E-06	1.49E-06
10	GO:0060249	anatomical structure homeostasis	15/95	425/18493	4.25E-09	2.71E-06	2.04E-06

10	GO:0090150	establishment of protein localization to membrane	13/95	317/18493	8.54E-09	3.55E-06	2.68E-06
10	GO:0000184	nuclear-transcribed mRNA catabolic process, nonsense-mediated decay	9/95	120/18493	1.11E-08	3.55E-06	2.68E-06
10	GO:0031647	regulation of protein stability	12/95	265/18493	1.11E-08	3.55E-06	2.68E-06
10	GO:0006457	protein folding	11/95	218/18493	1.56E-08	3.85E-06	2.90E-06
10	GO:0050821	protein stabilization	10/95	169/18493	1.61E-08	3.85E-06	2.90E-06
10	GO:0045841	negative regulation of mitotic metaphase/anaphase transition	6/95	36/18493	2.69E-08	5.72E-06	4.32E-06
10	GO:1902100	negative regulation of metaphase/anaphase transition of cell cycle	6/95	37/18493	3.20E-08	6.12E-06	4.62E-06
10	GO:0000819	sister chromatid segregation	10/95	185/18493	3.81E-08	6.62E-06	5.00E-06
10	GO:2000816	negative regulation of mitotic sister chromatid separation	6/95	39/18493	4.46E-08	7.10E-06	5.36E-06
10	GO:1905819	negative regulation of chromosome separation	6/95	40/18493	5.22E-08	7.41E-06	5.59E-06
10	GO:2001251	negative regulation of chromosome organization	9/95	144/18493	5.45E-08	7.41E-06	5.59E-06
10	GO:0006413	translational initiation	10/95	194/18493	5.97E-08	7.41E-06	5.59E-06
10	GO:1901990	regulation of mitotic cell cycle phase transition	13/95	375/18493	6.20E-08	7.41E-06	5.59E-06
10	GO:0006614	SRP-dependent cotranslational protein targeting to membrane	8/95	105/18493	6.66E-08	7.49E-06	5.66E-06
10	GO:0033048	negative regulation of mitotic sister chromatid segregation	6/95	42/18493	7.08E-08	7.52E-06	5.67E-06
10	GO:0006613	cotranslational protein targeting to membrane	8/95	109/18493	8.93E-08	8.98E-06	6.78E-06

10	GO:0033046	negative regulation of sister chromatid segregation	6/95	44/18493	9.45E-08	9.03E-06	6.82E-06
11	GO:0048002	antigen processing and presentation of peptide antigen	17/90	139/18493	1.68E-19	1.91E-16	1.43E-16
11	GO:0060333	interferon-gamma-mediated signaling pathway	15/90	90/18493	2.05E-19	1.91E-16	1.43E-16
11	GO:0019882	antigen processing and presentation	18/90	175/18493	3.20E-19	1.98E-16	1.49E-16
11	GO:0002478	antigen processing and presentation of exogenous peptide antigen	16/90	126/18493	1.14E-18	5.29E-16	3.97E-16
11	GO:0019884	antigen processing and presentation of exogenous antigen	16/90	134/18493	3.14E-18	1.17E-15	8.77E-16
11	GO:0034341	response to interferon-gamma	17/90	198/18493	7.52E-17	2.33E-14	1.75E-14
11	GO:0071346	cellular response to interferon-gamma	16/90	178/18493	3.17E-16	8.43E-14	6.32E-14
11	GO:0002495	antigen processing and presentation of peptide antigen via MHC class II	12/90	101/18493	6.93E-14	1.61E-11	1.21E-11
11	GO:0002504	antigen processing and presentation of peptide or polysaccharide antigen via MHC class II	12/90	102/18493	7.82E-14	1.62E-11	1.21E-11
11	GO:0019886	antigen processing and presentation of exogenous peptide antigen via MHC class II	11/90	98/18493	1.53E-12	2.85E-10	2.14E-10
11	GO:0019883	antigen processing and presentation of endogenous antigen	6/90	21/18493	5.74E-10	9.72E-08	7.28E-08
11	GO:0002483	antigen processing and presentation of endogenous peptide antigen	5/90	14/18493	4.72E-09	7.32E-07	5.48E-07
11	GO:0042590	antigen processing and presentation of exogenous peptide antigen via MHC class I	6/90	30/18493	6.06E-09	8.69E-07	6.51E-07
11	GO:0002475	antigen processing and presentation via MHC class Ib	5/90	16/18493	1.02E-08	1.36E-06	1.02E-06
11	GO:0002474	antigen processing and presentation of peptide antigen via MHC class I	6/90	46/18493	8.99E-08	1.12E-05	8.36E-06

11	GO:0006959	humoral immune response	12/90	349/18493	1.24E-07	1.44E-05	1.08E-05
11	GO:0002460	adaptive immune response based on somatic recombination of immune receptors built from immunoglobulin superfamily domains	12/90	354/18493	1.44E-07	1.45E-05	1.09E-05
11	GO:0001916	positive regulation of T cell mediated cytotoxicity	5/90	26/18493	1.48E-07	1.45E-05	1.09E-05
11	GO:0002479	antigen processing and presentation of exogenous peptide antigen via MHC class I, TAP-dependent	5/90	26/18493	1.48E-07	1.45E-05	1.09E-05
11	GO:0002697	regulation of immune effector process	13/90	439/18493	1.99E-07	1.85E-05	1.39E-05

Supplementary table5 The top 20 enrichment terms of the exemplar genes in other 9 cluster by NMF.
