# Supplementary material for: Immune and stromal scoring system associated with tumor microenvironment and prognosis: a gene-based multi-cancer analysis
Source: J Transl Med. 2021 Aug 3;19:330. doi: 10.1186/s12967-021-03002-1 (PMC8336334; doi:10.1186/s12967-021-03002-1)
Supplement: Supplementary file 14 — Additional file 14: Table S6. Immune and stromal gene signatures. [file 12967_2021_3002_MOESM14_ESM.pdf]

| Gene ID | Category |
|---|---|
| CD40LG | Immune-related |
| THEMIS | Immune-related |
| PYHIN1 | Immune-related |
| TRAT1 | Immune-related |
| FCRL1 | Immune-related |
| SPIB | Immune-related |
| GPR174 | Immune-related |
| SH2D1A | Immune-related |
| CCR4 | Immune-related |
| ITK | Immune-related |
| FCRL3 | Immune-related |
| C5orf20 | Immune-related |
| FIGF | Immune-related |
| UBASH3A | Immune-related |
| MS4A1 | Immune-related |
| CD300LG | Immune-related |
| SAMD3 | Immune-related |
| ADH1B | Immune-related |
| GZMK | Immune-related |
| CHRDL1 | Immune-related |
| ABI3BP | Immune-related |
| FCER2 | Immune-related |
| TIFAB | Immune-related |
| P2RY12 | Immune-related |
| CLEC10A | Immune-related |
| RSPO2 | Immune-related |
| PCDH15 | Immune-related |
| HLA-DOA | Immune-related |
| PLA2G2D | Immune-related |
| CLEC17A | Immune-related |
| CD3G | Immune-related |
| CCL19 | Immune-related |
| PTPRC | Immune-related |
| C17orf87 | Immune-related |
| GRIA1 | Immune-related |
| CD8A | Immune-related |
| PRG4 | Immune-related |
| P2RY13 | Immune-related |
| SFTPC | Immune-related |
| HLA-DPB1 | Immune-related |
| AADAC | Immune-related |
| EOMES | Immune-related |
| AOAH | Immune-related |
| CD1E | Immune-related |
| CCR2 | Immune-related |
| CCL5 | Immune-related |

| | |
|---|---|
| GFRA1 | Immune-related |
| TFEC | Immune-related |
| CLDN18 | Immune-related |
| FGL2 | Immune-related |
| C4orf7 | Immune-related |
| CD1B | Immune-related |
| GZMA | Immune-related |
| HLA-DPA1 | Immune-related |
| SCARA5 | Immune-related |
| PLEK | Immune-related |
| ZNF683 | Immune-related |
| CD19 | Immune-related |
| HLA-DRA | Immune-related |
| CD84 | Immune-related |
| PIK3CG | Immune-related |
| NCKAP1L | Immune-related |
| CR1 | Immune-related |
| WIF1 | Immune-related |
| CLEC12A | Immune-related |
| KIAA0408 | Immune-related |
| PIGR | Immune-related |
| CXCL13 | Immune-related |
| CD74 | Immune-related |
| TLR8 | Immune-related |
| CHIT1 | Immune-related |
| IL7R | Immune-related |
| HLA-DRB1 | Immune-related |
| HLA-DQA1 | Immune-related |
| COL6A6 | Immune-related |
| LYZ | Immune-related |
| SFTPA2 | Immune-related |
| CYBB | Immune-related |
| COL29A1 | Immune-related |
| LCP1 | Immune-related |
| IGJ | Immune-related |
| UBD | Immune-related |
| HLA-DQA2 | Immune-related |
| CXCL9 | Immune-related |
| PPYR1 | Immune-related |
| IFNG | Immune-related |
| MRC1 | Immune-related |
| FAM26F | Immune-related |
| B2M | Immune-related |
| CD1A | Immune-related |
| HLA-B | Immune-related |
| GBP5 | Immune-related |
| ADAMDEC1 | Immune-related |

| | |
|---|---|
| LAPTM5 | Immune-related |
| C1QB | Immune-related |
| ITGB2 | Immune-related |
| C1QA | Immune-related |
| SUCNR1 | Immune-related |
| MARCO | Immune-related |
| HLA-C | Immune-related |
| VSIG4 | Immune-related |
| F13A1 | Immune-related |
| CXCL10 | Immune-related |
| C1QC | Immune-related |
| HLA-A | Immune-related |
| CD163 | Immune-related |
| FCGR3A | Immune-related |
| TMSL3 | Immune-related |
| MDK | Stromal-related |
| RPL8 | Stromal-related |
| S100P | Stromal-related |
| FTH1 | Stromal-related |
| GPR87 | Stromal-related |
| UBC | Stromal-related |
| CLDN3 | Stromal-related |
| FGL1 | Stromal-related |
| TMSB10 | Stromal-related |
| HSPB1 | Stromal-related |
| ACTB | Stromal-related |
| ITPKA | Stromal-related |
| KRT19 | Stromal-related |
| S100A2 | Stromal-related |
| EEF1A2 | Stromal-related |
| PABPC1 | Stromal-related |
| RPLP0 | Stromal-related |
| HSP90AB1 | Stromal-related |
| NMU | Stromal-related |
| KRT8 | Stromal-related |
| KRT7 | Stromal-related |
| EPCAM | Stromal-related |
| YWHAZ | Stromal-related |
| MYH9 | Stromal-related |
| ACTG1 | Stromal-related |
| NPW | Stromal-related |
| MMP13 | Stromal-related |
| TUBA1B | Stromal-related |
| ERRFI1 | Stromal-related |
| DSP | Stromal-related |
| KRT18 | Stromal-related |
| P4HB | Stromal-related |

| | |
|---|---|
| ENO1 | Stromal-related |
| PKM2 | Stromal-related |
| RHOV | Stromal-related |
| ALDOA | Stromal-related |
| JUP | Stromal-related |
| LDHA | Stromal-related |
| HMGA1 | Stromal-related |
| HSPA1A | Stromal-related |
| MIF | Stromal-related |
| TUBB | Stromal-related |
| HMGB3 | Stromal-related |
| CYP24A1 | Stromal-related |
| TPI1 | Stromal-related |
| FAM83A | Stromal-related |
| RECQL4 | Stromal-related |
| FN1 | Stromal-related |
| VEGFA | Stromal-related |
| GAPDH | Stromal-related |
| TK1 | Stromal-related |
| TUBB3 | Stromal-related |
| UBE2C | Stromal-related |
| MYBL2 | Stromal-related |
| COL3A1 | Stromal-related |
| COL1A1 | Stromal-related |
| COL1A2 | Stromal-related |
| COL11A1 | Stromal-related |

**Supplementary table6** Immune and stromal gene signatures.
