# Supplementary material for: Immune and stromal scoring system associated with tumor microenvironment and prognosis: a gene-based multi-cancer analysis
Source: J Transl Med. 2021 Aug 3;19:330. doi: 10.1186/s12967-021-03002-1 (PMC8336334; doi:10.1186/s12967-021-03002-1)
Supplement: Supplementary file 15 — Additional file 15: Table S7. Gene overlap between our gene signatures and existing signatures. [file 12967_2021_3002_MOESM15_ESM.pdf]

Our signatures	Other signatures	Number of overlaps	Overlap genes	Study title	PMID
Stromal	Hypoxia-related signatures	9 (15.5%)	MIF, LDHA, VEGFA, TPI1, ALDOA, P4HB, GAPDH, ACTB, ACTG1	MALDI-Mass Spectrometric Imaging Revealing Hypoxia-Driven Lipids and Proteins in a Breast Tumor Model	25993305
Stromal	Differential expression genes in Hep-2 cells induced by fibroblast conditioned medium and fibroblasts induced by Hep-2 cell conditioned medium	9 (15.5%)	ENO1, YWHAZ, ACTB, ACTG1, GAPDH, RPLP0, FN1, COL1A1, P4HB	Genomics and Proteomics Approaches to the Study of Cancer-Stroma Interactions	20441585
Stromal	Collagen fibril organizing genes	7 (12.1%)	COL3A1, COL1A2, COL1A1, FN1, ACTB, ACTG1, GAPDH	A Subset of Myofibroblastic Cancer-Associated Fibroblasts Regulate Collagen Fiber Elongation, Which Is Prognostic in Multiple Cancers	26716418
Immune	Immune gene signatures	20 (18.5%)	CD84, CD74, TRAT1, CD19, CD163, CYBB, CCL5, CXCL9, GZMA, GZMK, CD3G, IGJ, FCGR3A, HLA-B, HLA-C, HLA-DPB1, HLA-DPA1, HLA-DRB1, HLA-DQA1, HLA-DRA	Conservation of Immune Gene Signatures in Solid Tumors and Prognostic Implications	27871313
Immune	Immune score signatures	15 (13.9%)	HLA-DOA, HLA-DQA2, HLA-DPB1, HLA-A, HLA-B, HLA-C, HLA-DPA1, HLA-DRB1, HLA-DRA, PTPRC, IFNG, GZMA, CCL5, CXCL10, CXCL9	Genomic and Immune Heterogeneity Are Associated With Differential Responses to Therapy in Melanoma	28819565
Immune	Genes are associated with immune cytolytic activity	11 (10.2%)	C1QC, C1QB, CXCL10, CXCL13, CXCL9, UBD, PLA2G2D, B2M, HLA-A, GZMK, GZMA	The Expression and Prognostic Impact of Immune Cytolytic Activity-Related Markers in Human Malignancies: A Comprehensive Meta-analysis	29515971

Supplementary table7 Gene overlap between our gene signatures and existing signatures.
