# Supplementary material for: Immune and stromal scoring system associated with tumor microenvironment and prognosis: a gene-based multi-cancer analysis
Source: J Transl Med. 2021 Aug 3;19:330. doi: 10.1186/s12967-021-03002-1 (PMC8336334; doi:10.1186/s12967-021-03002-1)
Supplement: Supplementary file 16 — Additional file 16: Table S8. Comparison with ESTIMATE and MCPcounter algorithms for prognosis and immunotherapy response prediction. [file 12967_2021_3002_MOESM16_ESM.pdf]

	Data set	Term	ISTMEscore		ESTIMATE			MCPCounter									
			immune score	stromal score	immune score	stromal score	ESTIMATE score	Tcell	CD8 +Tcell	Cytotoxic lymphocytes	NKcell	B lineage	Monocytic lineage	Myeloid dendritic cells	Neutrophils	Endothelial cells	Fibroblasts
Overall survival	TCGA LUAD	C-index (se)	0.578 (se = 0.022)	0.624 (se = 0.024)	0.578 (se = 0.023)	0.541 (se = 0.024)	0.568 (se = 0.023)	0.595 (se = 0.023)	0.549 (se = 0.024)	0.512 (se = 0.025)	0.533 (se = 0.026)	0.598 (se = 0.024)	0.508 (se = 0.025)	0.594 (se = 0.023)	0.538 (se = 0.025)	0.53 (se = 0.024)	0.535 (se = 0.024)
		Rank of C-index	5	1	5	9	7	3	8	14	12	2	15	4	10	13	11
	TCGA SKCM	C-index (se)	0.61 (se = 0.024)	0.578 (se = 0.023)	0.601 (se = 0.024)	0.544 (se = 0.024)	0.585 (se = 0.024)	0.572 (se = 0.024)	0.611 (se = 0.024)	0.59 (se = 0.023)	0.62 (se = 0.023)	0.556 (se = 0.024)	0.592 (se = 0.025)	0.602 (se = 0.023)	0.568 (se = 0.023)	0.527 (se = 0.024)	0.54 (se = 0.024)
		Rank of C-index	3	9	5	13	8	10	2	7	1	12	6	4	11	15	14
	TCAG HNSC	C-index (se)	0.542 (se = 0.021)	0.542 (se = 0.022)	0.542 (se = 0.021)	0.504 (se = 0.022)	0.528 (se = 0.022)	0.591 (se = 0.021)	0.551 (se = 0.021)	0.558 (se = 0.02)	0.555 (se = 0.022)	0.598 (se = 0.021)	0.502 (se = 0.022)	0.553 (se = 0.021)	0.521 (se = 0.023)	0.521 (se = 0.022)	0.517 (se = 0.022)
		Rank of C-index	7	7	7	14	10	2	6	3	4	1	15	5	11	11	13
	GSE 11969	C-index (se)	0.594 (se = 0.033)	0.613 (se = 0.033)	0.573 (se = 0.035)	0.545 (se = 0.035)	0.565 (se = 0.034)	0.561 (se = 0.034)	NA	0.561 (se = 0.034)	0.538 (se = 0.035)	0.574 (se = 0.035)	0.505 (se = 0.037)	0.56 (se = 0.033)	0.562 (se = 0.033)	0.57 (se = 0.034)	0.516 (se = 0.034)
		Rank of C-index	2	1	4	11	6	8		8	12	3	14	10	7	5	13
	GSE 68465	C-index (se)	0.532 (se = 0.021)	0.578 (se = 0.019)	0.522 (se = 0.021)	0.524 (se = 0.021)	0.524 (se = 0.021)	0.528 (se = 0.021)	0.518 (se = 0.021)	0.561 (se = 0.02)	0.55 (se = 0.02)	0.543 (se = 0.02)	0.564 (se = 0.021)	0.538 (se = 0.021)	0.51 (se = 0.021)	0.506 (se = 0.021)	0.518 (se = 0.02)
		Rank of C-index	7	1	11	9	9	8	12	3	4	5	2	6	14	15	12
	GSE 68571	C-index (se)	0.69 (se = 0.055)	0.667 (se = 0.059)	0.642 (se = 0.055)	0.599 (se = 0.058)	0.625 (se = 0.058)	0.539 (se = 0.07)	0.545 (se = 0.07)	0.506 (se = 0.068)	0.502 (se = 0.07)	0.577 (se = 0.071)	0.613 (se = 0.068)	0.523 (se = 0.072)	0.532 (se = 0.068)	0.501 (se = 0.066)	0.523 (se = 0.056)
		Rank of C-index	1	2	3	6	4	9	8	13	14	7	5	11	10	15	11
	GSE 37745	C-index (se)	0.531 (se = 0.027)	0.505 (se = 0.026)	0.511 (se = 0.027)	0.52 (se = 0.027)	0.513 (se = 0.028)	0.543 (se = 0.026)	0.561 (se = 0.027)	0.505 (se = 0.026)	0.536 (se = 0.026)	0.573 (se = 0.027)	0.53 (se = 0.026)	0.561 (se = 0.025)	0.524 (se = 0.024)	0.534 (se = 0.026)	0.505 (se = 0.025)
		Rank of C-index	7	13	12	10	11	4	2	13	5	1	8	2	9	6	13
	GSE 5008	C-index (se)	0.535 (se =	0.645 (se =	0.509 (se =	0.508 (se =	0.512 (se =	0.516 (se =	0.541 (se =	0.514 (se =	0.532 (se =	0.561 (se =	0.553 (se =	0.567 (se =	0.523 (se =	0.577 (se =	0.542 (se =

	**1**		0.038)	0.033)	0.039)	0.036)	0.039)	0.037)	0.036)	0.035)	0.036)	0.038)	0.038)	0.035)	0.037)	0.036)	0.037)
		Rank of C-index	8	1	14	15	13	11	7	12	9	4	5	3	10	2	6
	**GSE65904**	C-index (se)	0.612 (se = 0.031)	0.532 (se = 0.03)	0.614 (se = 0.03)	0.506 (se = 0.034)	0.561 (se = 0.032)	0.6 (se = 0.032)	0.578 (se = 0.028)	0.598 (se = 0.03)	0.611 (se = 0.029)	0.622 (se = 0.029)	0.559 (se = 0.035)	0.603 (se = 0.03)	0.54 (se = 0.031)	0.548 (se = 0.031)	0.553 (se = 0.032)
		Rank of C-index	3	14	2	15	9	6	8	7	4	1	10	5	13	12	11
	**Prognosis**	Mean C-index	0.5804444444	0.5871111111	0.565777778	0.532333333	0.553444444	0.560555556	0.55675	0.545	0.553	0.578	0.547333333	0.566777778	0.535333333	0.534888889	0.527666667
		Rank of mean C-index	2	1	5	14	8	6	7	11	9	3	10	4	13	12	15
Response to immunotherapy	**Immunotherapy Data Set 1**	Response pre-treatment AUC	0.5469	0.5586	0.5469	0.6406	0.5703	0.5312	0.4766	0.5391	0.4941	0.5117	0.5039	0.707	0.5703	0.6055	
		Response on-treatment AUC	0.9815	0.9074	0.9815	0.963	1	0.963	0.9444	0.963	0.6667	0.8889	1	0.8704	0.7037	0.9074	NA
		Rank of on-treatment AUC	3	9	3	5	1	5	8	5	14	11	1	12	13	9	
	**Immunotherapy Data Set 2 (GSE91061)**	Response pre-treatment AUC	0.602	0.5268	0.5468	0.5385	0.5719	0.6555	0.5711	0.5167	0.4699	0.5418	0.4967	0.5585	0.5435	0.6171	0.5385
		Response on-treatment AUC	0.7006	0.5897	0.6916	0.6568	0.6968	0.7574	0.6839	0.6968	0.6632	0.7548	0.6684	0.7239	0.5794	0.5871	0.5781
		Rank of on-treatment AUC	4	12	7	11	5	2	8	5	10	1	9	3	14	13	15
	**Immunotherapy Data**	Response pre-treatment AUC	0.5433	0.7467	0.5633	0.5533	0.53	0.5933	0.5967	0.7467	0.5767	0.5267	0.54	0.57	0.5933	0.6567	0.5233

**Set 3 (GSE93157)**	Rank of AUC	11	1	9	10	13	5	4	1	7	15	12	8	5	3	14
**Immunotherapy Data Set 4 (GSE67501)**	Response pre-treatment AUC	0.75	0.75	0.5357	0.6071	0.5	0.5714	0.6429	0.6429	0.6071	0.5714	0.6786	0.8214	0.6786	0.6071	0.6786
	Rank of AUC	2	2	14	9	15	12	7	7	9	12	4	1	4	9	4
**Immunotherapy Data Set 5 (GSE35640)**	Response pre-treatment AUC	0.6564	0.5989	0.635	0.6457	0.6337	0.7166	0.5388	0.5281	0.6925	0.5922	0.7861	0.643	0.5227	0.516	0.512
	Rank of AUC	4	9	7	5	8	2	11	12	3	10	1	6	13	14	15
**Immunotherapy response**	Mean AUC	0.72636	0.71854	0.68142	0.68518	0.6721	0.72034	0.68134	0.7155	0.64124	0.6668	0.73462	0.72574	0.61554	0.65486	0.573
	Rank of mean AUC	2	5	8	7	10	4	9	6	13	11	1	3	14	12	15

Supplementary table8 Comparison with ESTIMATE and MCPcounter algorithms for prognosis and immunotherapy response prediction.
