# Supplementary material for: Immune and stromal scoring system associated with tumor microenvironment and prognosis: a gene-based multi-cancer analysis
Source: J Transl Med. 2021 Aug 3;19:330. doi: 10.1186/s12967-021-03002-1 (PMC8336334; doi:10.1186/s12967-021-03002-1)
Supplement: Supplementary file 17 — Additional file 17: Table S9. Table of acronyms. [file 12967_2021_3002_MOESM17_ESM.pdf]

Acronym	Full name
TCGA	The Cancer Genome Atlas
GEO	Gene Expression Omnibus
LUAD	lung adenocarcinoma
SKCM	skin cutaneous melanoma
HNSC	head and neck squamous cell carcinoma
TME	tumor microenvironment
RMA	robust multi-array average
SD	stable disease
RNA-seq	RNA-sequencing
RECIST	Response Evaluation Criteria in Solid Tumors
TMB	Tumor mutation burden
CNA	Copy number alteration
NMF	Non-negative matrix factorization
MTL	Multitask learning
DGE	different gene expression analysis
GSEA	Gene set enrichment analysis
ssGSEA	Single-sample gene set enrichment analysis
CRLCN	Cells-receptors-ligands communication networks
MOCDE	Molecular Complex Detection
ESTIMATE	Estimation of STromal and Immune cells in MAlignant Tumours using Expression data
PSM	propensity score matching
ORA	over-representation analysis
GO	Gene Ontology
ROC	receiver operating characteristic
FDR	false discovery rate
LDF	low dimensional features
CNACB	CNA count burden

Supplementary table9 Table of acronyms.
