# Supplementary material for: Immune and stromal scoring system associated with tumor microenvironment and prognosis: a gene-based multi-cancer analysis
Source: J Transl Med. 2021 Aug 3;19:330. doi: 10.1186/s12967-021-03002-1 (PMC8336334; doi:10.1186/s12967-021-03002-1)
Supplement: Supplementary file 18 — Additional file 18. Additional Bioinformatics Analysis. [file 12967_2021_3002_MOESM18_ESM.docx]

**Additional Bioinformatics Analysis**

# Methods

## Trajectory analysis

Trajectory analysis was performed by monocle package of R [[1](#_bookmark0)].

## K-Nearest Neighbor (KNN) Based on Mahalanobis Distance

We used the ratio of IFNG, ITGB2, TLR8 to VEGFA as input variable for KNN model and only 1% characterized patients with top VEGFAhigh ITGB2low TLR8low IFNGlow or VEGFAlow ITGB2high TLR8high IFNGhigh were considered as training samples. To eliminate the influence of collinearity among variables, we adopted Mahalanobis instead of Euclidean distance in the KNN.

## Lasso regression

Lasso regression contributed to avoid over fitting, which was implemented by R glmnet package [[2](#_bookmark1)].

## Clustering of time-series

Gene clustering of time-series data was identified by R Mfuzz package [[3](#_bookmark2)].

## Weighted gene co-expression network analysis (WGCNA)

WGCNA was an effective method to recognize gene expression patterns by R WGCNA package [[4](#_bookmark3)].

## Immunohistochemistry (IHC)

The IHC of ACTB/ACTG1 was collected from HPA database ([www.proteinatlas.org/)](http://www.proteinatlas.org/)) .

# Results

## 2.1 Modules of Stromal Related Genes Based on WGCNA

The 58 stromal related genes were divided into the 4 modules via WGCNA analysis based on TCGA LUAD, SKCM and HNSC cohorts (**Figure S1A**). The eigenvalues of each module were positively correlated with stromal scores (**Figure S1B-D**). The characteristics of each module were as follows.

## 2.1.1 ECM-HGF/cMET-EMT/MMPs Module

The 6 genes of module 1 were involved in the extracellular matrix (ECM) compound and matrix metalloproteinases (MMP), containing COL1A1, COL1A2, COL3A1, COL11A1, FN1 and MMP13.

The HH and LH patients had significantly higher expression levels of above 6 genes in LUAD and SKCM (P < 0.0001, **Figure S2A**), expect HNSC. We then performed a combined analysis across the 3 tumors. All the 6 genes were significantly associated with higher pathological stages (**Figure S2B)**, and worse survival (**Figure S2C**), expect FN1.

As previous studies, ECM transmitted signals to cells via integrin-mediated pathway, which can activate HGF/cMET-PTK2 signaling pathway and MMPs secretion [[5](#_bookmark4)]. In this study, we found COL1A1, COL1A2, COL3A1 and FN1 had significant co-expression relationship with HGF (P < 0.05, **Figure S2D**). Beyond that, COL1A1, COL1A2 and COL3A1 were significantly correlated with PTK2 ( P < 0.05, **Figure S2E**). At the level of copy number alteration (CNA), we found that HGF expression was reduced in patients with COL1A1 deletions (**Figure S3A**). Moreover, the patients with amplification of COL1A1, COL1A2 and COL11A1 showed higher c-MET.

The HGF/c-Met signaling pathway played an adverse role in prognosis by inducing EMT [[6](#_bookmark5)].We next analyzed the expression of EMT markers in patients with different TME subtypes. As expected, the LH and HH patients showed higher N-cadherin, Snail1, MMP-9, Twist and lower E-cadherin, Vinmentin and ZO-1, which suggested the occurrence of EMT (**Figure S3B**). Moreover, the amplification of module 1 genes improved the expression of Snail1 and Twist in CNA level (**Figure S3C**).

Patients were divided into three groups: high (all 6 gene expressions were high), low (all 6 gene expressions were low) and medium (others). As expected, the GSEA showed higher cell adhesion mediated by integrin, MET activates PTK2 and MMPs signal enriched in the high group (**Figure S4A**) which was linked to worse survival (**Figure S4B**).

**2.1.2 Glycolysis-acidmicroenvironment-AKT/mTOR Module**

The 6 genes of module 2 were involved in the glycolytic enzymes, containing ENO1, ALDOA, LDHA, TPI1, PKM2 and GAPDH (**Figure S5**). Due to the Warburg effect, malignant tumors tend to be glycolytic, even if oxygen is sufficient [[7](#_bookmark6)]. Previous shown that glycolysis of tumors was induced by HIF1A [[8](#_bookmark7)]. We found that there was a co-expression relationship between LDHA/PKM2 and HIF1A (**Figure S6A**). High expression of these enzymes had adverse effects on prognosis; and these enzymes was increased in advanced tumors (**Figure S6B**). Similar to module 1, we also divided patients into three groups: high, medium and low based on both above 6 gene expression, which showed significantly prognostic differences (**Figure S6C**).

Enhanced glycolysis promotes the formation of acidic microenvironment [[9](#_bookmark8)]. We used a new data GSE101988 with culture medium of different PH (6.3 and 7.4, **Figure S7A**) to further analysis. Similar to previous studies, GSEA and time-course analysis showed acidic microenvironment induces P38MAPK cascade, AKT1-E17K, mTOR signals (**Figure S7B & C**) which were the important cancerous pathways.

## 2.1.3 VEGF-P38MAPK-HSPBP1/ACTG1/ACTB Module

HSPBP1 and actin-related genes (ACTG1 and ACTB) also were included in module 2. Previous studies showed that VEGF-P38MAPK pathway can activate HSPBP1 and actin (ACTG1 and ACTB) to promote endothelial cell migration [[10](#_bookmark9)]. Considering that VEGFA was also one of the stroma related gene in this study, VEGF and downstream P38MAPK might be involved in signal activation.

In this study, we demonstrated RNA expression of ACTG1/ACTB in TCGA LUAD and ACTB in TCGA SKCM were higher than normal tissue (**Figure S8A**). Moreover, ACTG1 and ACTB were also higher in tumor tissues based on IHC from HPA database (**Figure S8B**); and ACTG1/ACTB had adverse effects on prognosis (**Figure S8C)**.

Previous studies showed that P38MAPK specific inhibitors (SB203580) inhibited cell migration by 80% [[10](#_bookmark9)]. We next used the data GSE50591 [11] including samples with/without SB203580 treatment for 1, 4, 24, 48 hours. The clustering of time-series data was used to identify the pattern of gene changing with time via R Mfuzz (**Figure S9A**). We found that ACTG1/ACTB genes were included in gene cluster 1, which showed higher P38MAPK cascade, actin assembly, Epithelium migration, response to TGF-β and EGF via enrichment analysis (**Figure S9B**). After using SB203580, the expression of ACTG1/ACTB and HSPBP1 decreased (**Figure S9C**). Moreover, mutation of P38MAPK and its downstream (MAPKAPK2 and MAPKAPK3) were linked to low expression of HSPBP1 (**Figure S9D & E**).

## 2.1.4 HMGA1 & HMGB3-immunoregulation Module

We found that high mobility proteins (HMGA1 and HMGB3) were mainly concentrated in module 3. The HMGA1 and HMGB1 were known regulator of inflammatory and immune response [[12](#_bookmark10), [13](#_bookmark11)]. Through BLAST analysis, we found that all transcripts of HMGB3 and HMGB1 had 72% homology (**Figure S10**). Next, we studied the effects of HGMA1 and HGMB3 on immunity. HGMB3 and HGMA1 have negative effects on the infiltration of immune cells, including B cells, Macrophage, Neutrophil and Dendritic cells (**Figure S11**). In the CNA analysis, the immune cell infiltration of diploid HMGB3 and HGMA1 was higher than that of deletion or amplification, except Dendritic cells infiltration (**Figure S12**). Module 4 was mainly enriched in keratin, which was an important marker of tumors rather than microenvironment cells, therefore we didn't performed further analysis for module 4.

## 2.2 VEGFA, ITGB2, IFNG and TLR8 were Key Markers of Our 4 TME Subtypes

The above WGCNA analysis revealed the stroma related gene patterns (**Figure S13A**). Through the protein interaction network via STRING database [14], we identified the hub genes connecting the immune and stromal subnetworks (**Figure S13B**). To further identify core regulatory genes, we performed Lasso liner regression to fit immune and stromal scores in TCGA LUAD, SKCM and HNSC cohorts (**Figure S14**). Only VEGFA, ITGB2, IFNG and TLR8 still had coefficients in both immune (**Figure S13C**) and stromal (**Figure S13D**) Lasso models.

To further analyze the relationship between dynamic marker gene (VEGFA, ITGB2, IFNG and TLR8) changes and TME subtypes, we next performed unsupervised trajectory analysis in TCGA cohorts. Interestingly, the HL patients were located at one end of the pseudotime, while the LH patients were located at opposite ends (**Figure S15A**). We also noticed the HH and LL concentrated in the middle of the path. With the increase of pseudotime, VEGFA were on the up-regulation which contrasted with ITGB2, IFNG and TLR8 (**Figure S15B**), suggesting the characteristic of HL was VEGFAlow ITGB2high IFNGhigh TLRhigh which was contrary to the LH patients.

Next, we tried to divide the patient into the three patterns via K-nearest neighbor (KNN) model (KNNH: VEGFAlow ITGB2high IFNGhigh TLRhigh; KNNL: VEGFAhigh ITGB2low IFNGlow TLRlow and KNNM: intermediate states). The survival curve showed distinction of different TME marker patterns (**Figure S15C**). The KNNH, KNNL represented the HL and LH, respectively (**Figure S16**). The KNNH patients also had significantly favorable prognosis than KNNL in validation datasets (all, P < 0.05, **Figure S17**) [15,16].

In terms of the expression of immune related molecules, we found higher CD40LG and lower TIM-3, Treg markers (Foxp3, CD25) in the KNNH (**Figure S18**). However, the KNNH patients were linked to higher PD-1, which may be the induction of IFNG. In addition, KNNH also showed low VEGFA.

# 3 Figures


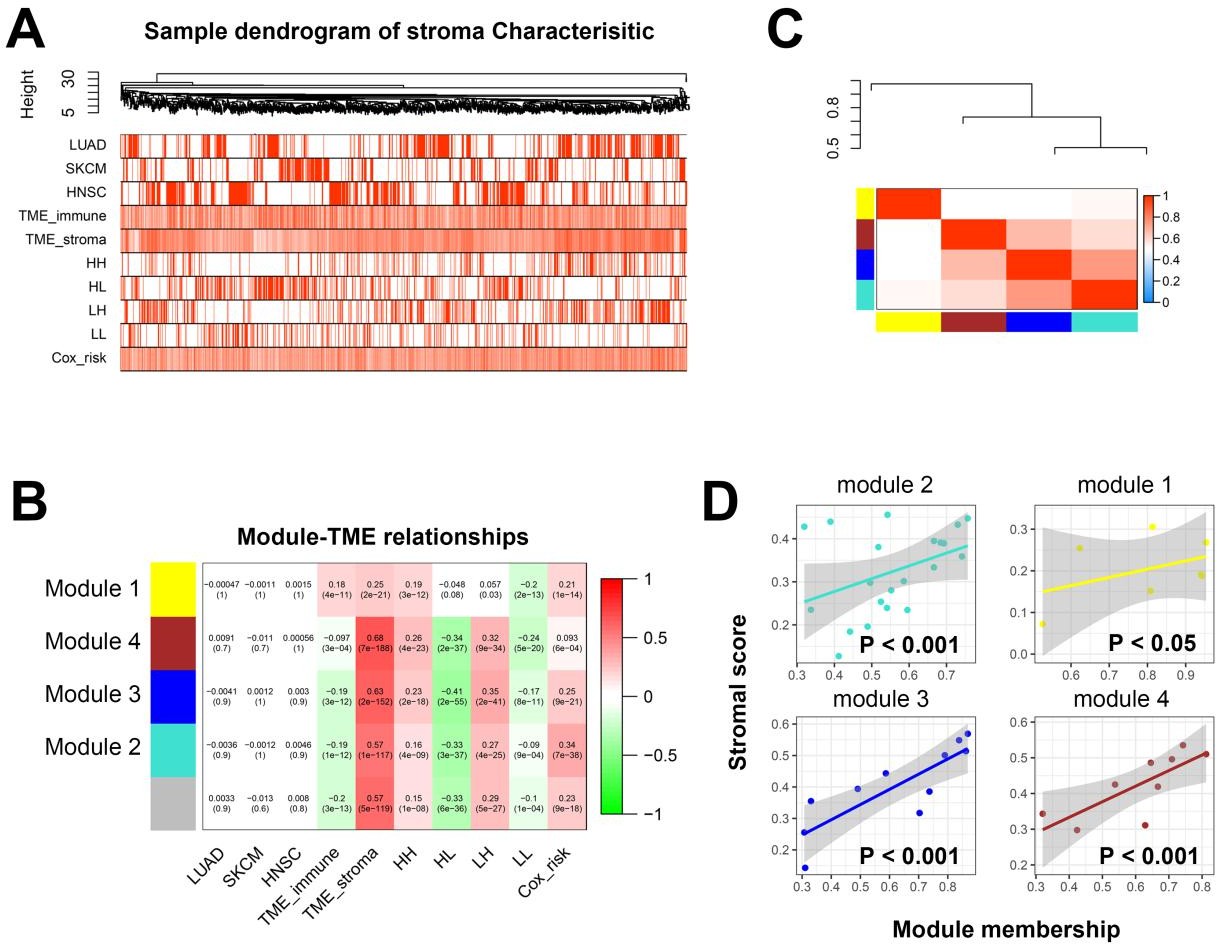


**Figure S1. The WGCNA analysis of stromal related genes in TCGA LUAD, SKCM and HNSC cohorts. (A)** The sample cluster tree of LUAD, SKCM and HNSC; **(B)** The relationship between module eigenvalues and phenotypes; **(C)** The clustering of modules; **(D)** The correlation between module eigenvalues and stromal score. LUAD, lung adenocarcinoma; SKCM, skin cutaneous melanoma; HNSC, head and neck squamous cell carcinoma; TCGA, The Cancer Genome Atlas.


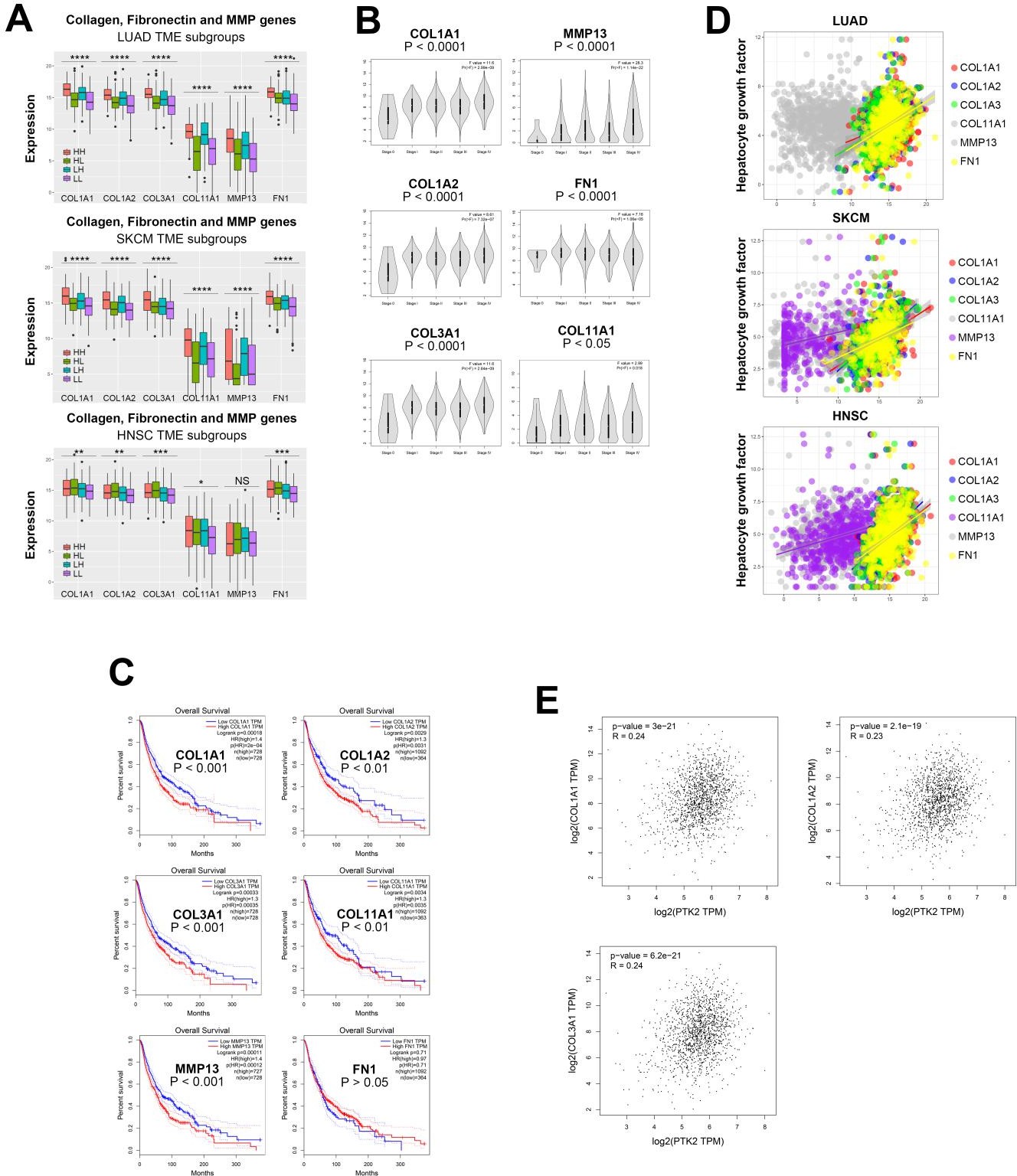


**Figure S2. The comprehensive analysis of module 1 genes from TCGA cohorts. (A)** The boxplot of module 1 genes in the 4 subtypes; **(B)** The relationship between module 1 genes and TNM stages; **(C)** The Kaplan-Meier survival plot of module 1 genes; **(D)** The correlation between module 1 genes and HGF; **(E)** The correlation between COL1A1/COL1A2/COL3A1 and PTK2. LUAD, lung adenocarcinoma; SKCM, skin cutaneous melanoma; HNSC, head and neck squamous cell carcinoma; TCGA, The Cancer Genome Atlas.


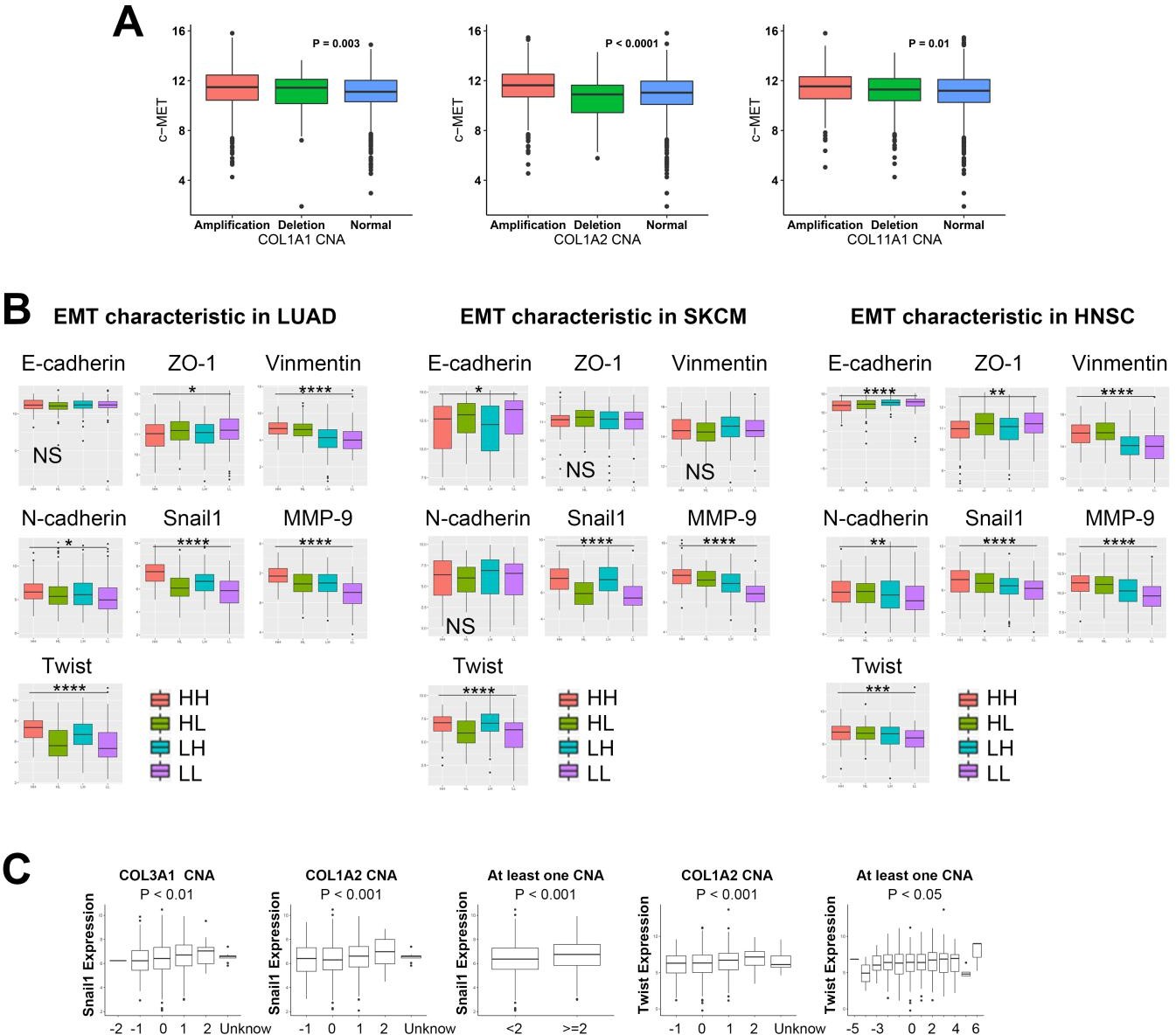


**Figure S3. The EMT characteristic in module 1 from TCGA cohorts. (A)** The relationship between CNA of COL1A1, COL1A2, COL11A1 and c-MET expression; **(B)** The EMT characteristic of the 4 subtypes; **(C)** The relationship between CNA of COL1A1, COL1A2, COL11A1 and EMT marker expression. CNA, copy number alteration; TCGA, The Cancer Genome Atlas.


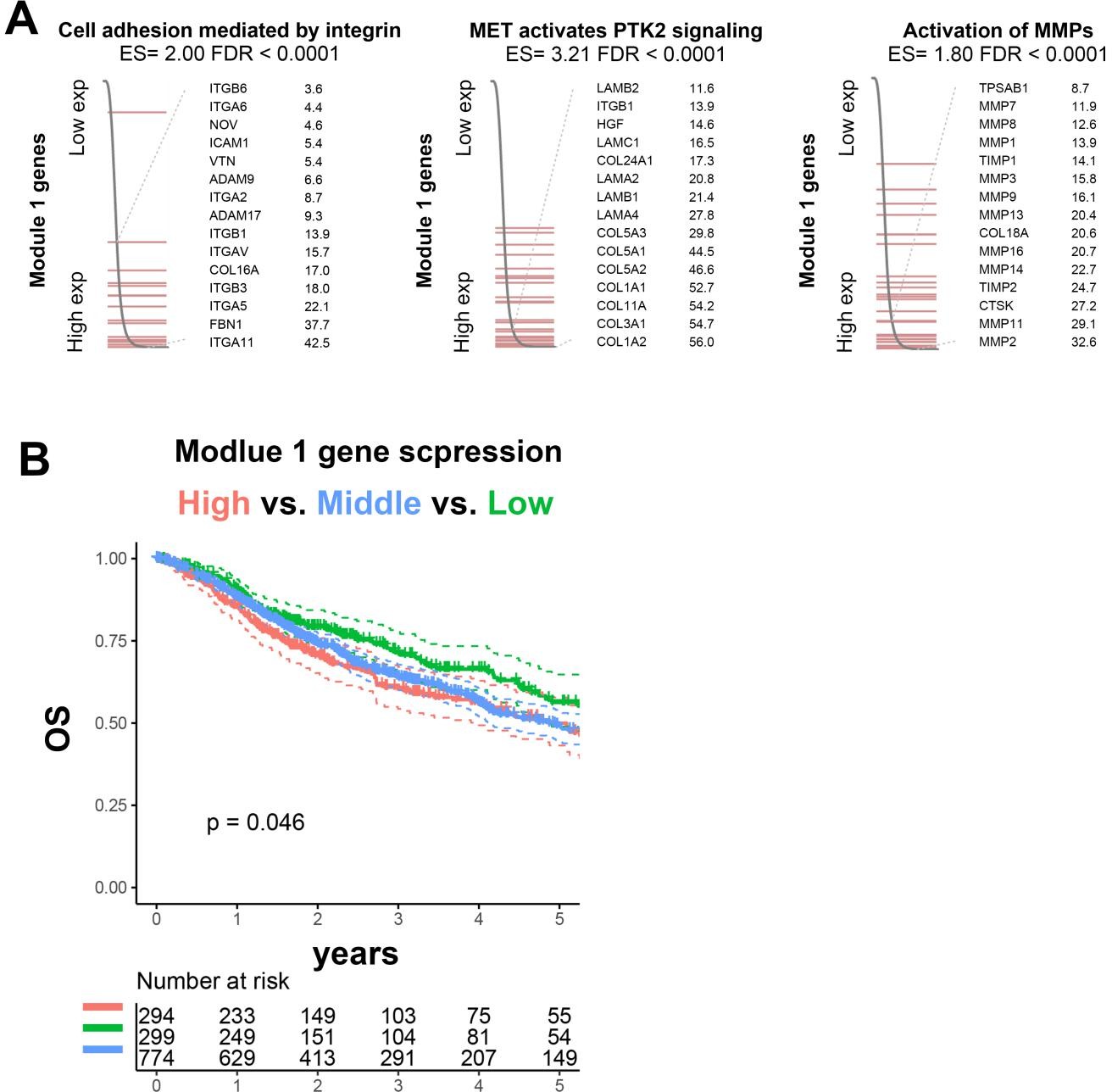


**Figure S4. The COL1A1high COL1A2high COL3A1high COL11A1high FN1high patients showed worse survival in TCGA cohorts. (A)** The GSEA of high-low COL1A1/COL1A2/COL3A1/COL11A1/FN1 expression; **(B)** The OS of high-low COL1A1/COL1A2/COL3A1/COL11A1/FN1 expression. GSEA, gene set enrichment analysis; TCGA, The Cancer Genome Atlas.


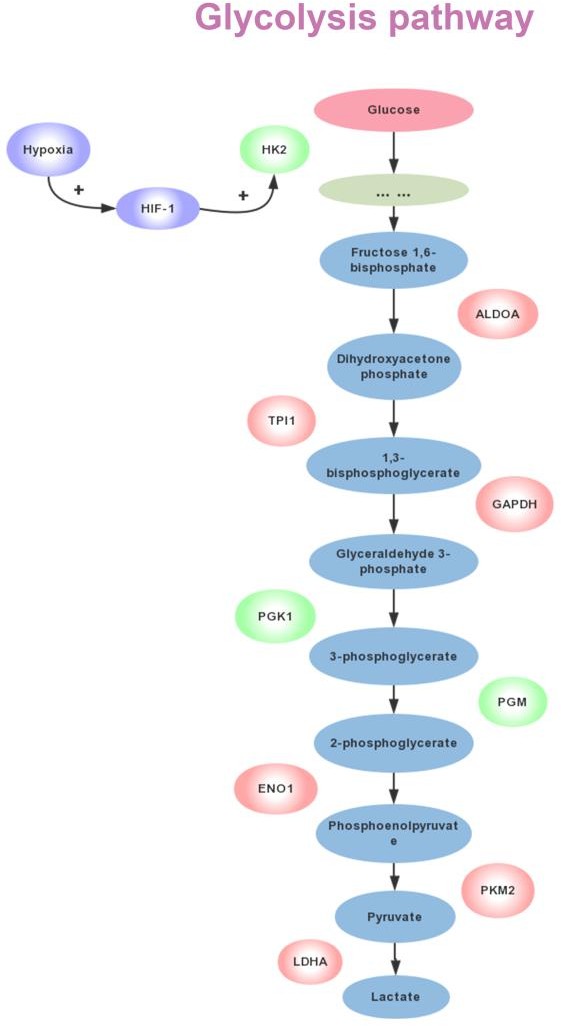


**Figure S5. Glycolysis pathway sketch map.** Red was our stromal-related genes.


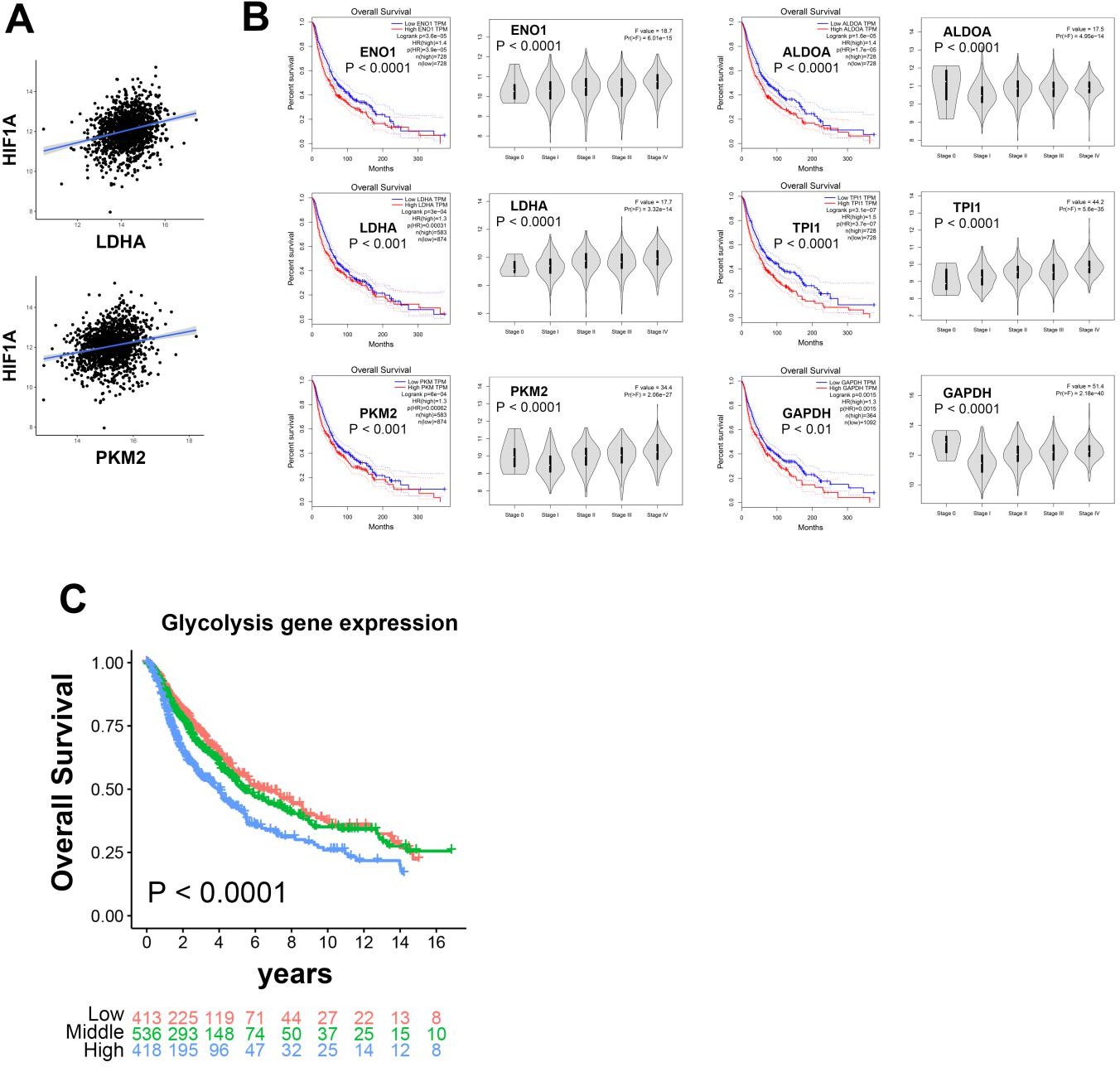


## Figure S6. The characteristic of glycolysis-related genes in module 2 from TCGA LUAD, SKCM and HNSC cohorts.

**(A)** The LDHA and PKM2 were correlated with HIF1A; **(B)** The relationship between module 2 genes and TNM stages or survival; **(C)** The OS of different ENO1/ALDOA/LDHA/TPI1/PKM2/GAPDH expression; TCGA, The Cancer Genome Atlas.


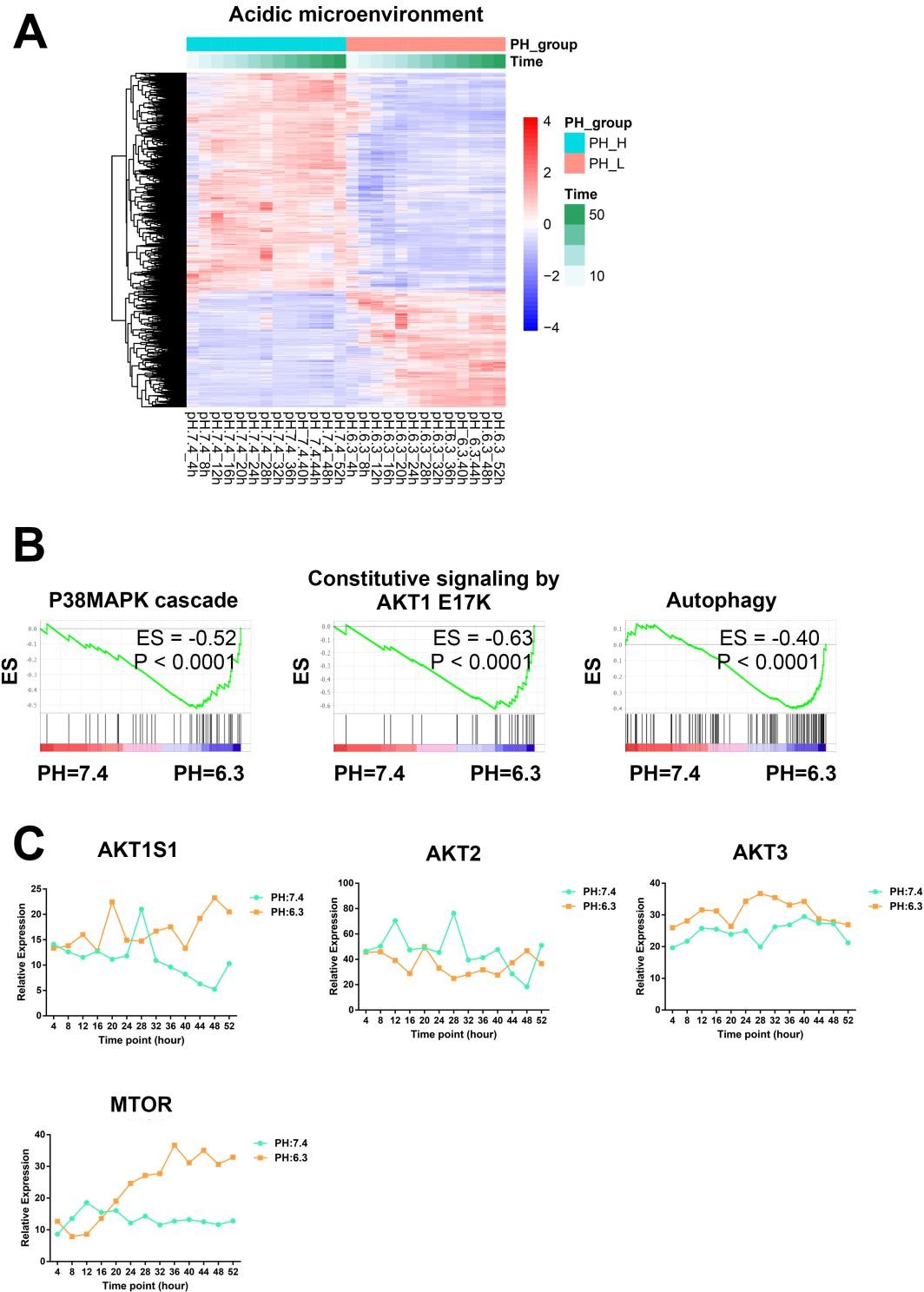


**Figure S7. Acidic microenvironment induced AKT/mTOR signal. (A)** The heatmap of GSE101988; **(B)** The GSEA of different PH; **(C)** The expression of AKT1S1/AKT2/AKT3/mTOR in different time points. GSEA, gene set enrichment analysis.


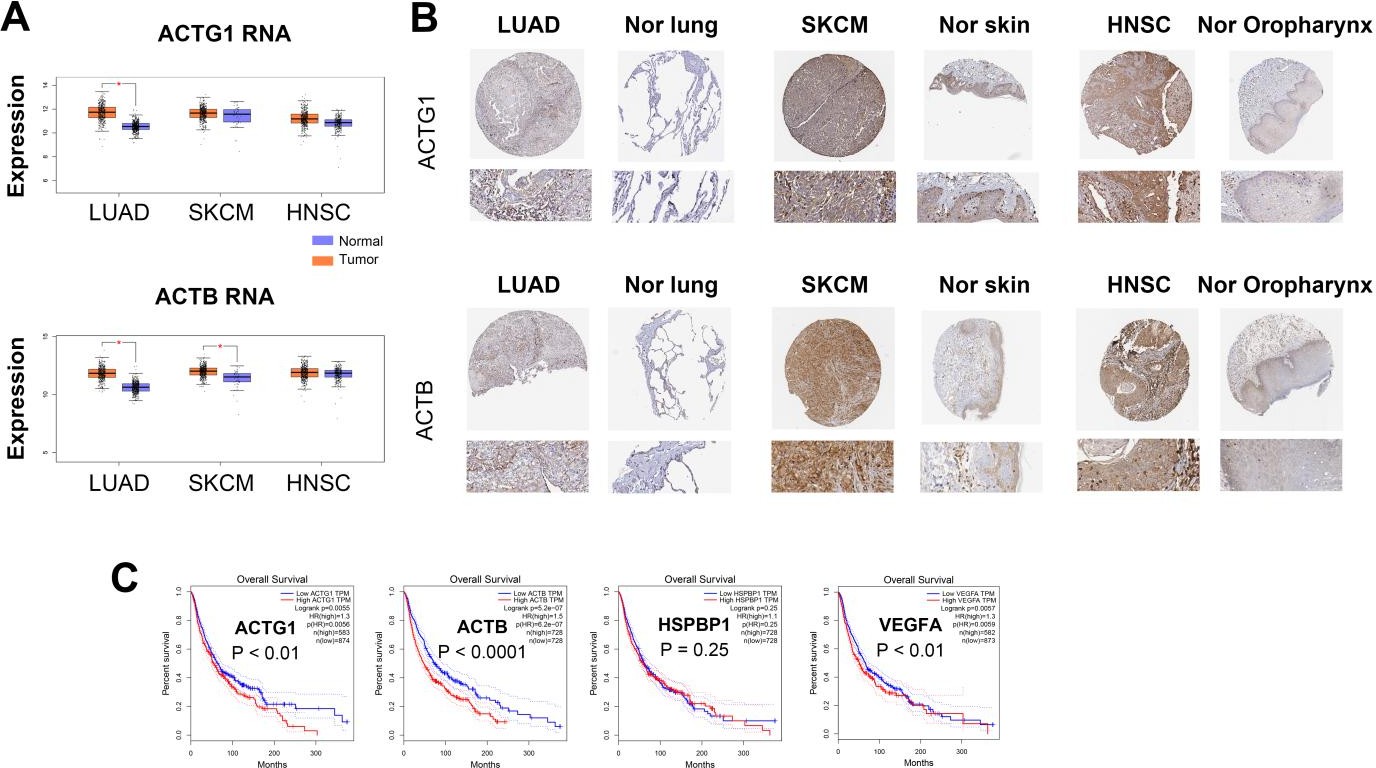


**Figure S8. Preliminary analysis of ACTG1, ACTB, HSPBP1 and VEGFA. (A)** The RNA expression of ACTG1 and ACTB in normal and tumor tissues; **(B)** The IHC of ACTG1 and ACTB in normal and tumor tissues from HPA database; **(C)** The survival curves of ACTG1, ACTB, HSPBP1 and VEGFA expression. LUAD, lung adenocarcinoma; SKCM, skin cutaneous melanoma; HNSC, head and neck squamous cell carcinoma; TCGA, The Cancer Genome Atlas.


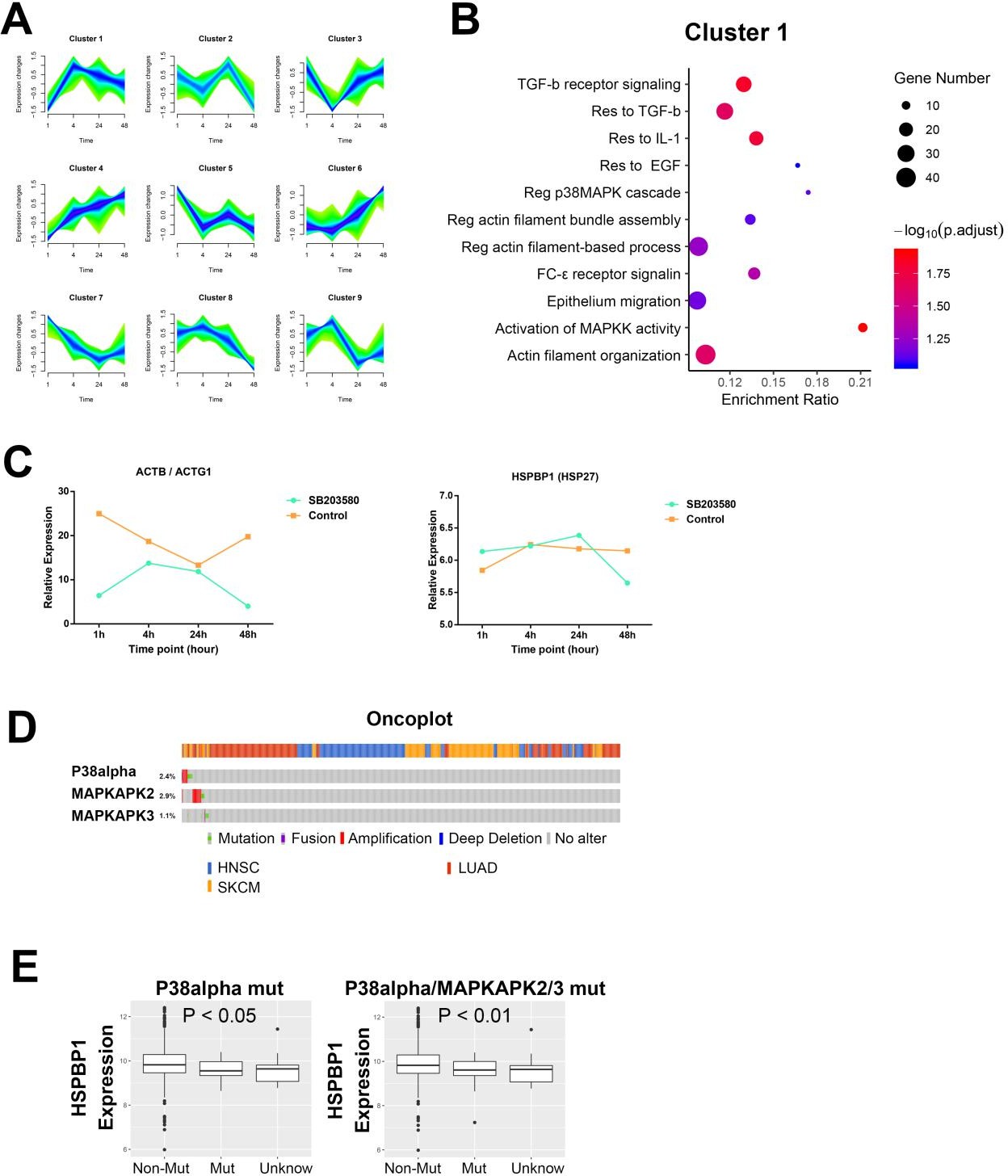


## Figure S9. Comprehensive analysis of VEGF-P38MAPK-HSPBP1/ACTG1/ACTB pathway.

**(A)** The gene clusters by time-course analysis; **(B)** The enrichment analysis of cluster 1 (containing ACTG1 and ACTB); **(C)** The expression of ACTG1, ACTB and VEGFA with or without P38MAPK specific inhibitors (SB203580); **(D)** The mutation and CNA of P38MAPK, MAPKAPK2 and MAPKAPK3; **(E)** Mutation of P38MAPK and its downstream (MAPKAPK2 and MAPKAPK3) were linked to low expression of HSPBP1. CNA, copy number alteration.


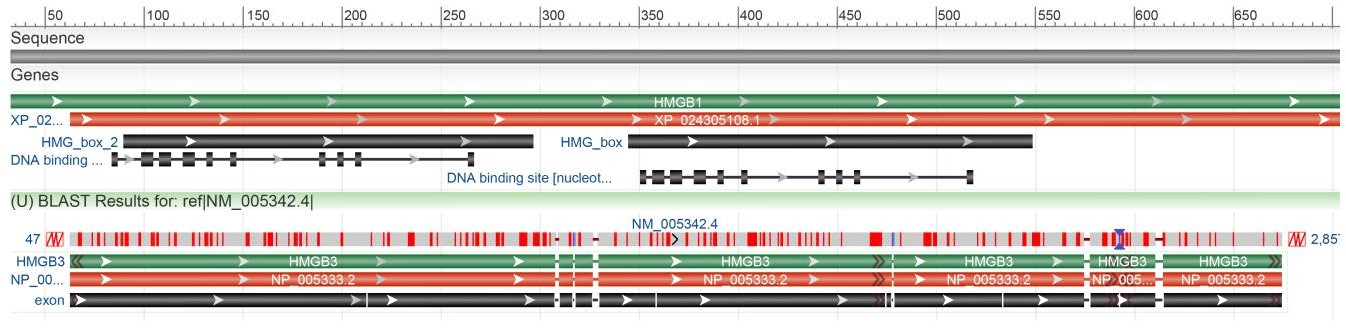


## Figure S10. The BLAST analysis between HMGB3 and HMGB1.


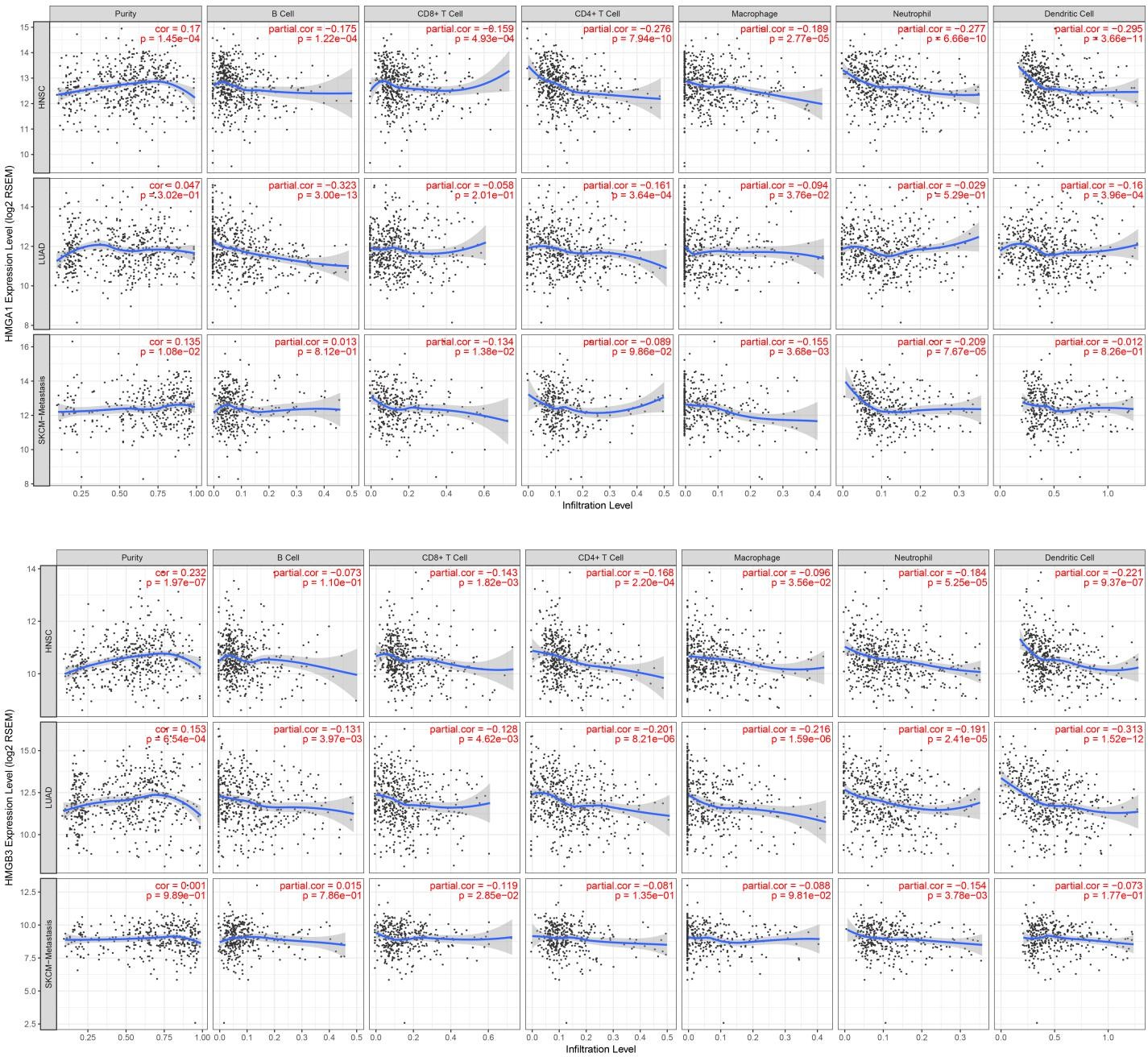


**Figure S11. HGMB3 and HGMA1 have negative effects on the infiltration of immune cells in TCGA cohorts**. TCGA, The Cancer Genome Atlas.


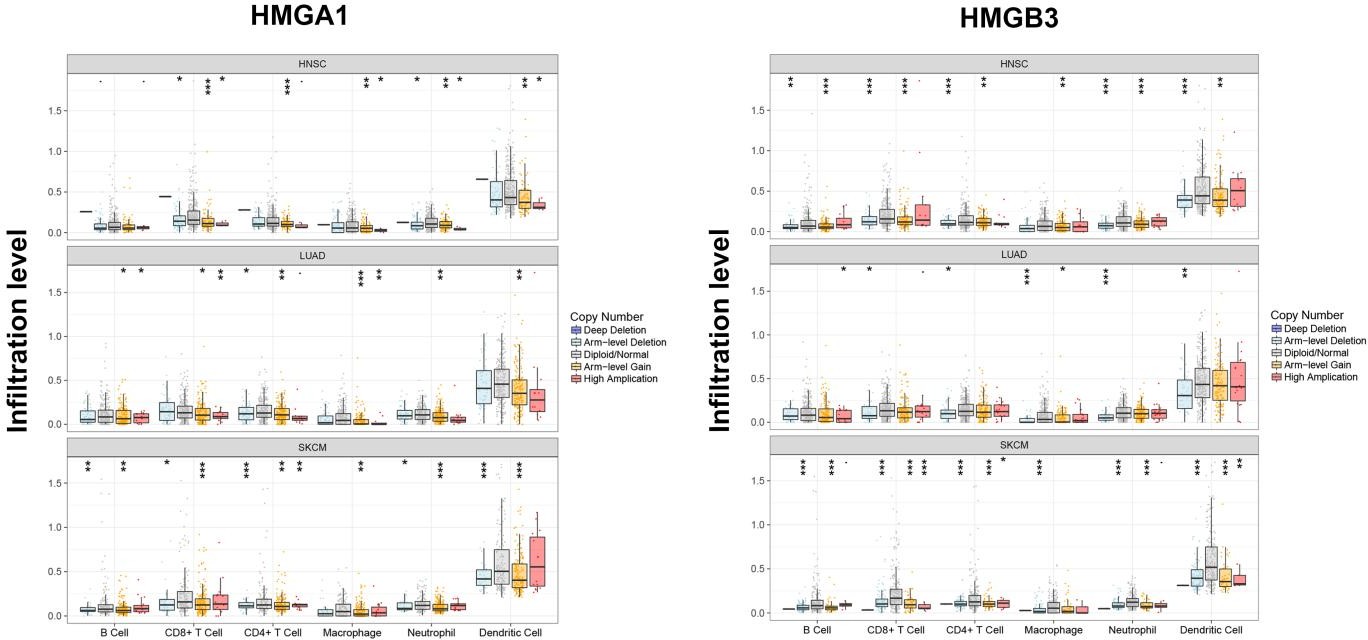


**Figure S12. The relationship between infiltration of microenvironment cells and CNA of HMGB3 and HMGA1 in TCGA cohorts.** CNA, copy number alteration; TCGA, The Cancer Genome Atlas.


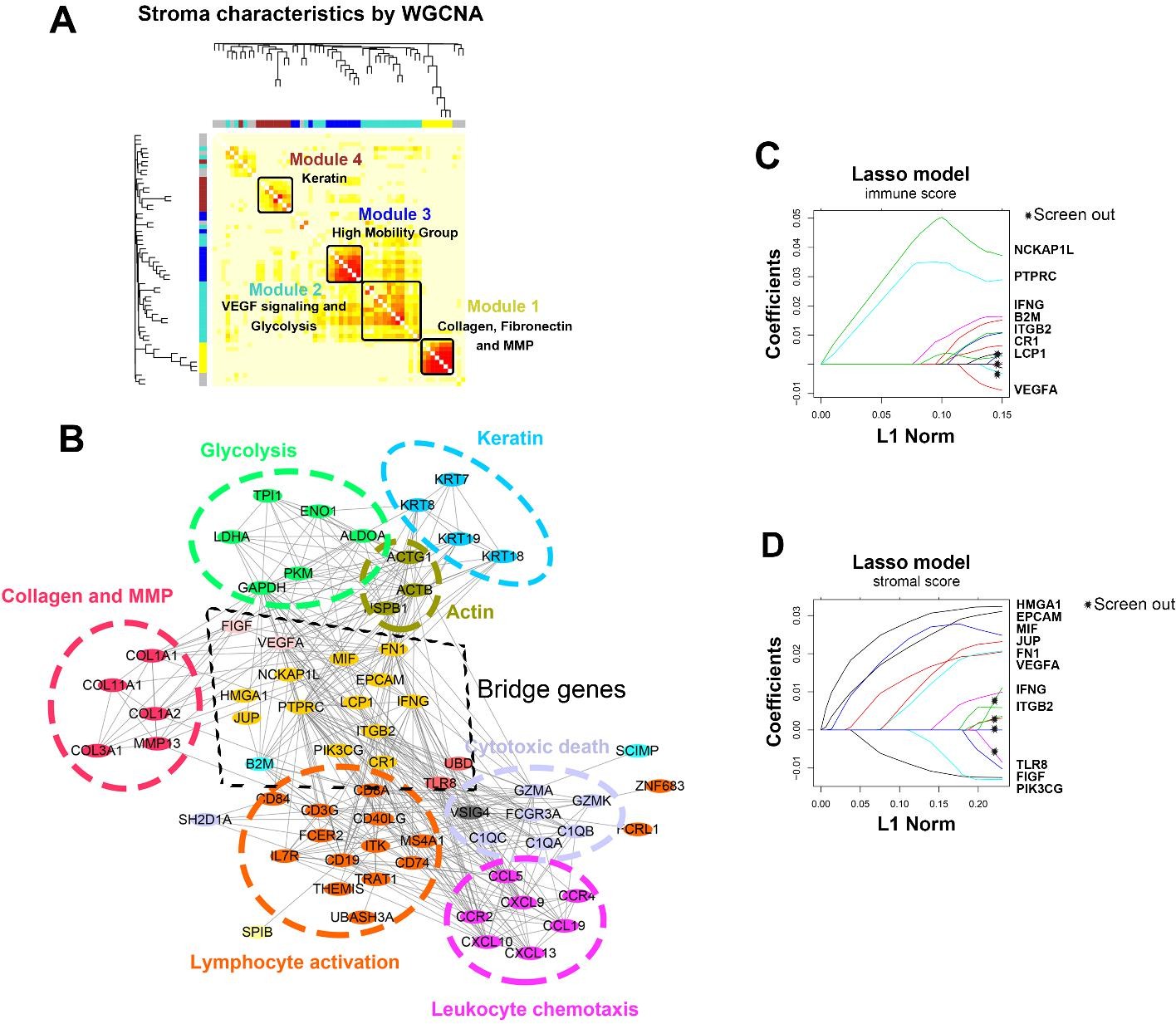


**Figure S13. The Core pathways of stroma microenvironment based on systems biology. (A)** The stroma-related gene patterns by WGCNA in TCGA cohorts; **(B)** the protein interaction network of TME-related genes; **(C)** The Lasso regression model of immune score; **(D)** The Lasso regression model of stromal score.


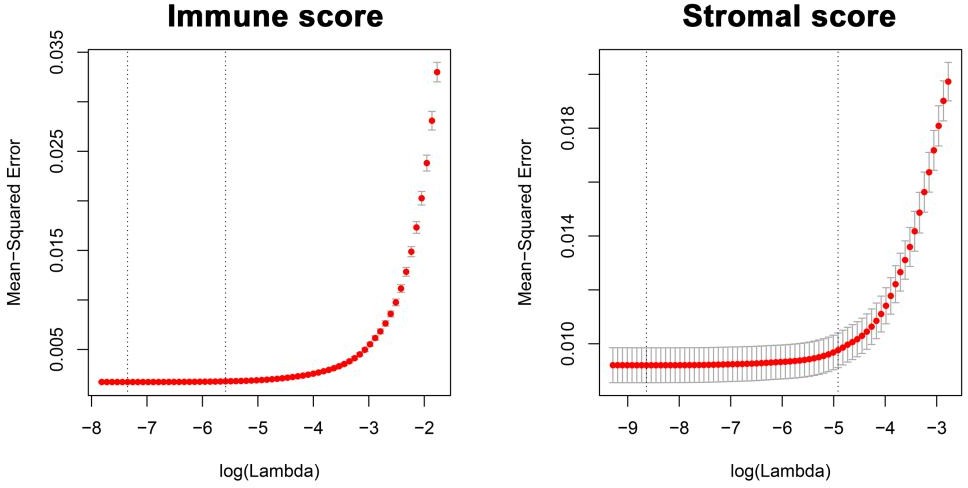


## Figure S14. The selection of parameter lambda in Lasso liner regression model.


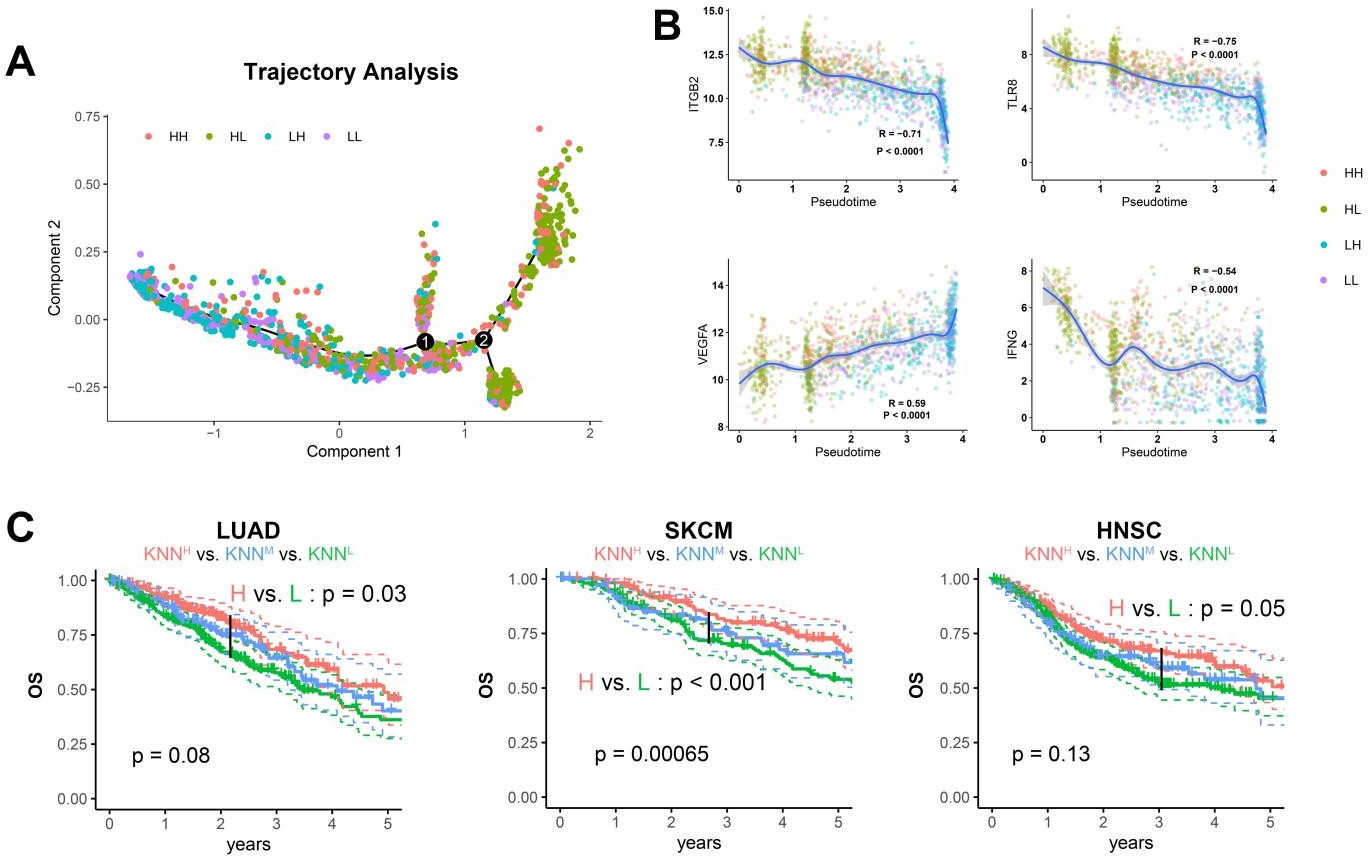


**Figure S15. The VEGFA, ITGB2, IFNG and TLR8 were TME markers. (A)** The Trajectory analysis identified the TME markers (VEGFA, ITGB2, IFNG and TLR8) in TCGA LUAD (n = 501), TCGA SKCM (n = 352), TCGA HNSC (n = 514); **(B)** The correlation between markers and pseudotime; **(C)** The survival curve of TME marker patterns (KNNH: VEGFAlow ITGB2high IFNGhigh TLRhigh; KNNL: VEGFAhigh ITGB2low IFNGlow TLRlow and KNNM: intermediate state). TME, tumor microenvironment; LUAD, lung adenocarcinoma; SKCM, skin cutaneous melanoma; HNSC, head and neck squamous cell carcinoma; TCGA, The Cancer

Genome Atlas.


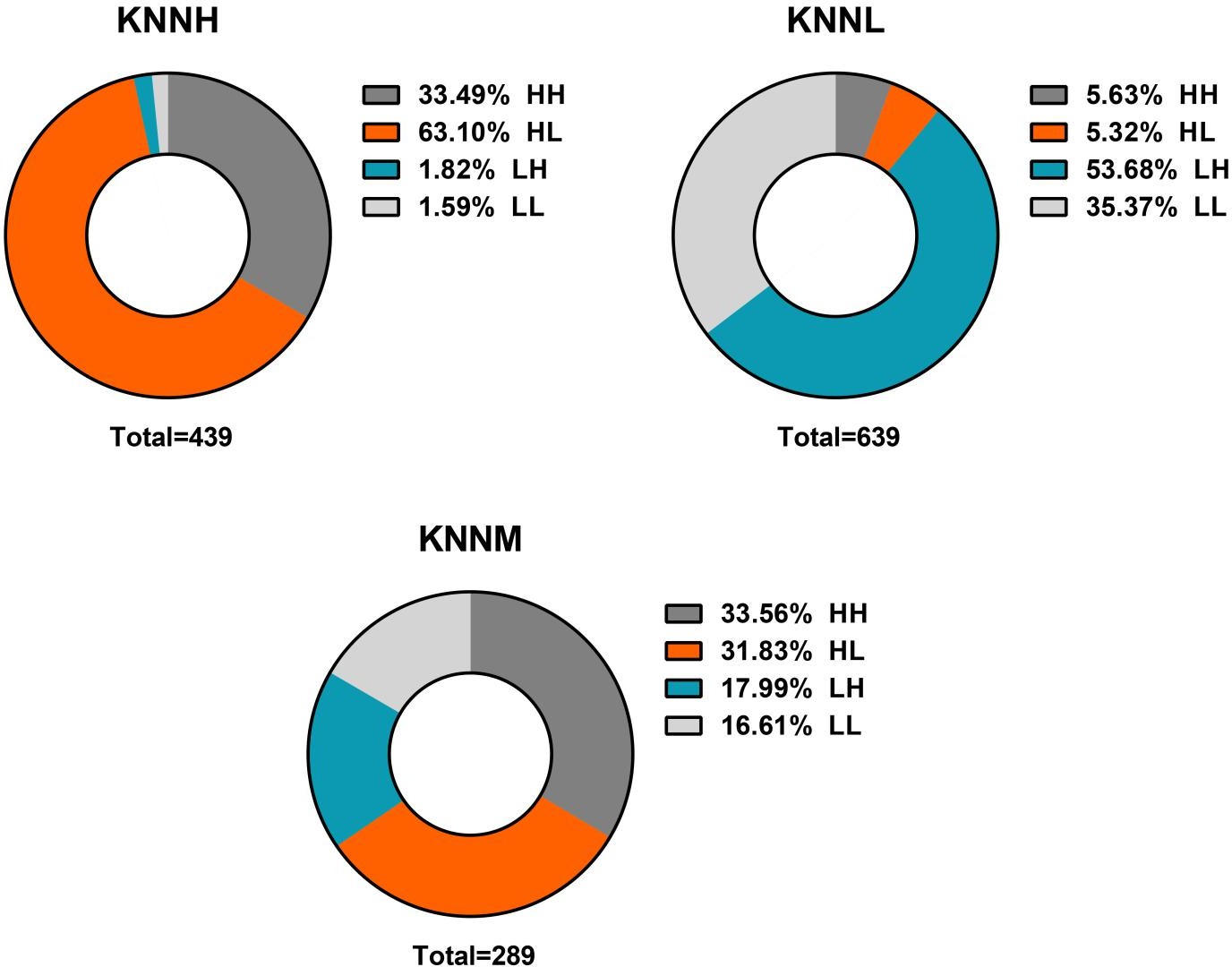


## Figure S16. The TME subtypes proportion in KNN group from TCGA cohorts. KNN,

k-nearest neighbor model; KNNH, VEGFAlow ITGB2high IFNGhigh TLRhigh; KNNL: VEGFAhigh ITGB2low IFNGlow TLRlow; KNNM, intermediate state; HH, immunehigh stromalhigh; HL, immunehigh stromallow; LH, immunelow stromalhigh; LL, immunelow stromallow; TCGA, The Cancer Genome Atlas.


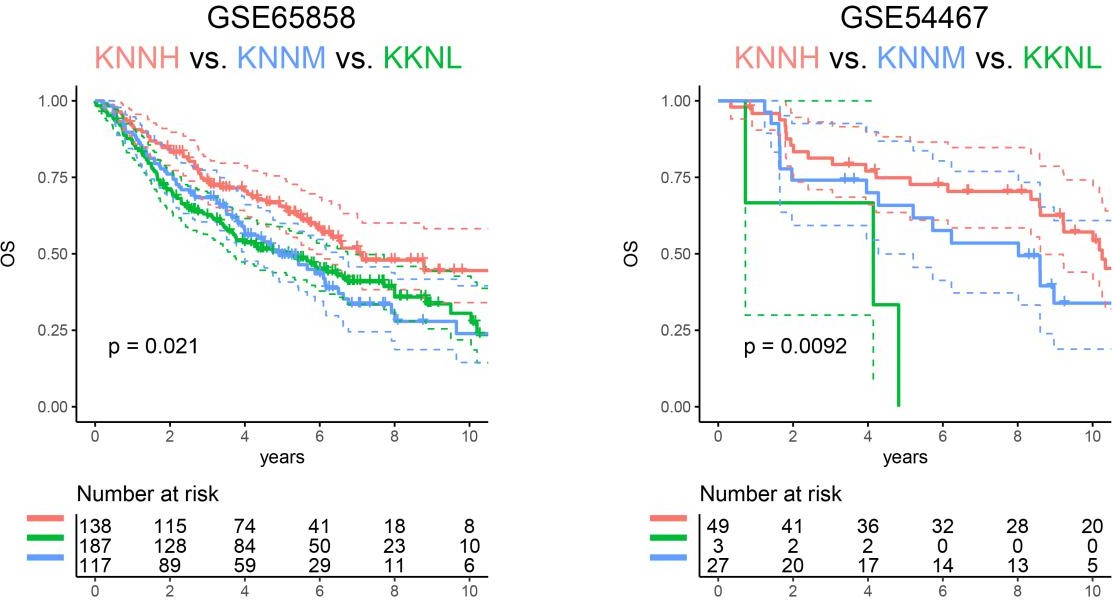


**Figure S17. The Kaplan-Meier survival plot of KNN groups in validation cohorts.** KNNH, VEGFAlow ITGB2high IFNGhigh TLRhigh; KNNL: VEGFAhigh ITGB2low IFNGlow TLRlow; KNNM, intermediate state.


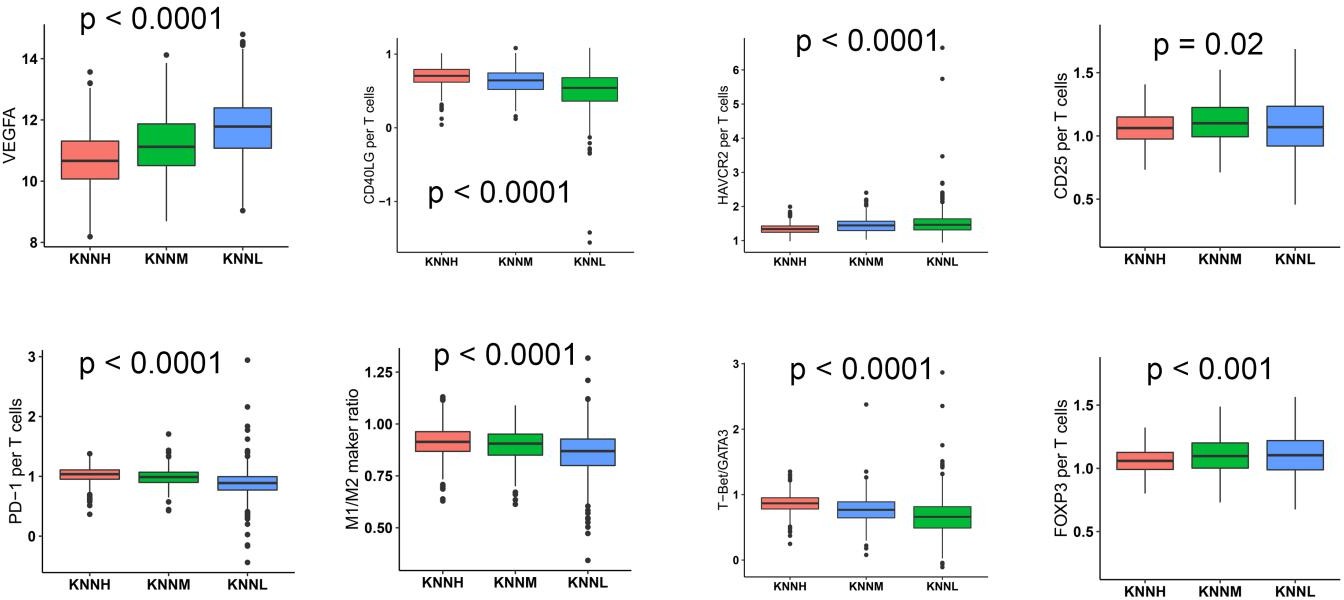


**Figure S18. The boxplot of immune- and stromal- related molecules in KNN groups from TCGA cohorts.** KNNH, VEGFAlow ITGB2high IFNGhigh TLRhigh; KNNL: VEGFAhigh ITGB2low IFNGlow TLRlow; KNNM, intermediate state.

# 4 References

1. Trapnell C, Cacchiarelli D, Grimsby J, Pokharel P, Li S, Morse M, et al. The dynamics and regulators of cell fate decisions are revealed by pseudotemporal ordering of single cells. Nature biotechnology. 2014;32(4):381-6.
2. Friedman J, Hastie T, Tibshirani R. Regularization Paths for Generalized Linear Models via Coordinate Descent. Journal of statistical software. 2010;33(1):1-22.
3. Kumar L, Futschik M. Kumar L, Futschik E.. Mfuzz: a software package for soft clustering of microarray data. Bioinformation 2: 5-7. Bioinformation. 2007;2:5-7.
4. Langfelder P, Horvath S. WGCNA: an R package for weighted correlation network analysis. BMC bioinformatics. 2008;9:559.
5. Beviglia L, Kramer RH. HGF induces FAK activation and integrin-mediated adhesion in MTLn3 breast carcinoma cells. International journal of cancer. 1999;83(5):640-9.
6. Jiao D, Wang J, Lu W, Tang X, Chen J, Mou H, et al. Curcumin inhibited HGF-induced EMT and angiogenesis through regulating c-Met dependent PI3K/Akt/mTOR signaling pathways in lung cancer. Molecular therapy oncolytics. 2016;3:16018.
7. Vander Heiden MG, Cantley LC, Thompson CB. Understanding the Warburg effect: the metabolic requirements of cell proliferation. Science (New York, NY). 2009;324(5930):1029-33.
8. Blum R, Jacob-Hirsch J, Amariglio N, Rechavi G, Kloog Y. Ras inhibition in glioblastoma down-regulates hypoxia-inducible factor-1alpha, causing glycolysis shutdown and cell death. Cancer research. 2005;65(3):999-1006.
9. Bohme I, Bosserhoff AK. Acidic tumor microenvironment in human melanoma. Pigment cell & melanoma research. 2016;29(5):508-23.
10. Rousseau S, Houle F, Huot J. Integrating the VEGF signals leading to actin-based motility in vascular endothelial cells. Trends in cardiovascular medicine. 2000;10(8):321-7.
11. Gazel A , Nijhawan R I , Walsh R , et al. Transcriptional profiling defines the roles of ERK and p38 kinases in epidermal keratinocytes. Journal of Cellular Physiology, 2010, 215(2):292-308.
12. Schuldenfrei A, Belton A, Kowalski J, Talbot CC, Jr., Di Cello F, Poh W, et al. HMGA1 drives stem cell, inflammatory pathway, and cell cycle progression genes during lymphoid tumorigenesis. BMC genomics. 2011;12:549.
13. Fiuza C, Bustin M, Talwar S, Tropea M, Gerstenberger E, Shelhamer JH, et al. Inflammation-promoting activity of HMGB1 on human microvascular endothelial cells. Blood. 2003;101(7):2652-60.
14. Damian S , Andrea F , Michael K , et al. The STRING database in 2011: functional interaction networks of proteins, globally integrated and scored. Nuclc Acids Research, 2011, 39(Database issue):561-8.
15. Wichmann G, Rosolowski M, Krohn K, et al. The role of HPV RNA transcription, immune response-related gene expression and disruptive TP53 mutations in diagnostic and prognostic profiling of head and neck cancer. Int J Cancer. 2015;137(12):2846-2857.
16. Jayawardana K, Schramm SJ, Haydu L, et al. Determination of prognosis in metastatic melanoma through integration of clinico-pathologic, mutation, mRNA, microRNA, and protein information. Int J Cancer. 2015;136(4):863-874.
